# Fluorogenic Sydnones for Bioorthogonal Labeling of DNA

**DOI:** 10.1002/chem.71314

**Published:** 2026-06-23

**Authors:** Kerstin Müller, Hans‐Achim Wagenknecht

**Affiliations:** ^1^ Institut of Organic Chemistry Karlsruhe Institute of Technology (KIT) Karlsruhe Germany

**Keywords:** cyanine dye, DNA, fluorescence, RNA, synthesis

## Abstract

Five cyanine‐styryl dyes were synthetically conjugated to sydnones combining fluorogenicity with bioorthogonal reactivity. The synthesis was based on synthetic modules and building blocks using Heck couplings as central conjugation steps. The novel cyanine‐styryl dyes show significant Stokes shifts to well separate excitation and emission for fluorescent cell imaging. Reaction kinetics and fluorogenicity of the strain‐promoted sydnone‐alkyne cycloaddition (SPSAC) with 7‐deazadenosine, 7‐deaza‐2’‐deoxyadenosine and DNA modified by bicyclo[6.1.0]non‐4‐yne (BCN) were investigated by spectroscopic means. The determined second‐order rate constants k_2_ with nucleoside and 2‐deoxynucleoside were found in a range from k_2_ = 0.4±0.05 M^−1^s^−1^ to 7.0±0.3 M^−1^s^−1^. The reaction is accompanied by fluorescence turn‐on up to 8. SPSAC with BCN‐modified DNA showed significantly enlarged fluorescence turn‐on of up to 17 and significant acceleration of reaction kinetics up to k_2_ = 2,300 M^−1^s^−1^ due to the DNA template effect. The bioorthogonality of fluorogenic SPSAC was evidenced by fluorescence labeling of HeLa cells transfected by BCN‐modified DNA. The fluorescence of one SPSAC product showed a red‐shift of 123 nm which is a potentially useful for metabolic labeling of RNA and DNA because it helps to distinguish the labeling of the modified nucleosides from the labeling of modified DNA by the fluorescence color as readout inside cells.

## Graphical abstract

Conjugates of sydnones with cyanine‐styryl dyes combine bioorthogonal reactivity with fluorogenicity. The reactivity with bicyclo[6.1.0]non‐4‐yne (BCN)‐modified DNA showed second‐order rate constants of up to k_2_ = 2,300 M^−1^s^−1^ and fluorescence turn‐on by one magnitude of order. The sydnone‐dyes are cell permeable and can undergo reactions with BCN‐modified DNA in the HeLa cells.

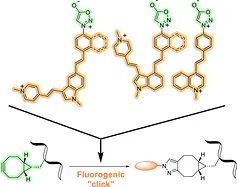

## 1 Introduction

Over the past decade, bioorthogonal click reactions have emerged as cornerstone technology for the study of proteins, glycans and nucleic acids in cells and organisms [1, 2, 3, 4, 5, 6, 7, 8, 9, 10]. There are continuous efforts constructing new bioorthogonal chemistry, those without the need for catalysis are preferred for labeling in biological cells to avoid cytotoxic side effects of metal ions [11]. Sydnones as highly stable mesoionic compounds were discovered by Early and Mackney in 1935 [12]. They are masked 1,3‐dipoles and have a significant potential for bioorthogonal labeling due to their abiotic structure and their high stability under physiological conditions [13, 14, 15, 16, 17]. Other attractive features are their synthetic modularity for tunable reactivity, and potential photo‐activation for spatial and temporal control of their chemical reactivity [18]. The bioorthogonality is delivered by the reaction of sydnones with cyclooctynes and bicyclo[6.1.0]non‐4‐yne (BCN), called strain‐promoted sydnone‐alkyne cycloaddition (SPSAC) [14, 15, 19, 20]. An additional advantage is the extrusion of CO_2_ making the reaction entropically favored and finally irreversible. This has been demonstrated for labeling of proteins and complex glycans [14, 21]. Guianvarc'h et al. have applied SPSAC chemistry for fluorescent labeling of DNA [22], Munier, Knerr et al. for DNA conjugation [23], and we evidenced bioorthogonality of SPSAC by experiments *in cellulo* [24]. In the latter studies, sydnones were attached as covalent modification to DNA.

Bioorthogonal labeling of nucleic acids in general and metabolic labeling of DNA and RNA in living cells or even live animals requires fluorogenicity by a significant fluorescence turn‐on because excess dye cannot be washed away. Tetrazine‐modified dyes undergo inverse electron demand Diels–Alder (IEDDA) reactions with 5‐vinyl‐2’‐deoxyuridine in metabolically labeled DNA [25]. Fluorogenicity was achieved either by “probes for imaging nucleosidic alkene groups” (PINK) [26] or by conjugates with nucleic‐acid sensitive cyanine‐styryl dyes [27, 28]. Cyclopropenes allow to combine fluorogenic IEDDA reactivity [29] with photoclick labeling [30]. BCN and trans‐cyclooctene are significantly faster reactive moieties that can also be used with fluorogenic tetrazines [31]. With respect to sydnones as bioorthogonally reactive moieties, their most attractive feature for chemical biology and fluorescent imaging is their fluorogenicity enabling visualization of biomolecules [13, 32, 33]. Friscourt et al. applied the quenching effect of sydnones on coumarin and restored their fluorescence by reaction with BCN by two orders of magnitudes [32]. The resulting pyrazole cycloadducts were found to be highly fluorescent with good quantum yields and large Stokes shifts. Taran et al. developed iminosydnones as fluorogenic probes for no‐wash labeling of the cyclooctyne‐modified myoprotein in cells [13, 33]. The use of sydnones for fluorogenic and bioorthogonal labeling of DNA *in cellulo*, however, was not yet achieved. This task requires photostable and more importantly nucleic acid‐sensitive dyes. Cyanine‐styryl dyes fulfill both requirements [34, 35, 36, 37]. Non‐covalent binding of this type of dyes to nucleic acids additionally increases their fluorogenicity and accelerates their reactivity by the template effect [37]. We call this concept two‐factor fluorogenicity and evaluated it in Diels‐Alder reactions with inverse electron demand with tetrazine‐cyanine‐styryl conjugates [27, 28]. Herein, we extend this two‐factor fluorogenicity concept to sydnone‐conjugates **1–5** of cyanine‐styryl dyes as fluorogenic probes for bioorthogonal labeling of DNA (Figure 1). We evaluate the reaction kinetics and the fluorescence turn‐on by their reaction with a BCN‐modified nucleoside, 2’‐deoxynucleoside and DNA, and proof bioorthogonality by experiments *in cellulo*.

**FIGURE 1:**
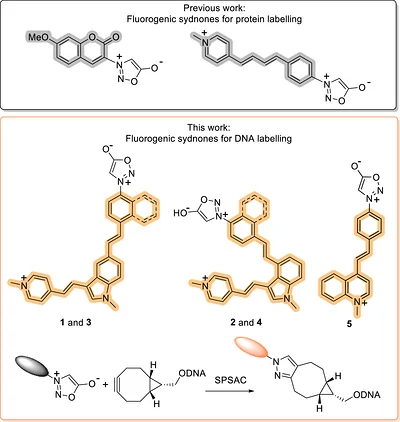
Fluorogenic sydnones for protein labeling by SPSAC from previous work [13, 32, 33] and fluorogenic sydnones **1**–**5** for DNA labeling in this work.

## 2 Results and Discussion

### 2.1 Synthesis of Sydnone‐Cyanine‐Styryl Conjugates **1–5**

The five conjugates **1**–**5** of sydnones with cyanine styryl dyes were synthesized following a modular approach using a Heck coupling as central step (Scheme 1). Accordingly, synthesis of the first two sydnones **1** and **2** was straightforward because it combines three literature‐known building blocks **6**–**8**. Sydnone building block **6** was prepared following the published procedure by Taran et al. [33], the brominated cyanine‐building blocks **7** [28] and **8** [27] by our own published procedures. Their Pd‐catalyzed couplings gave sydnone **1** in 49% yield and **2** in 45% yield, respectively.

**SCHEME 1:**
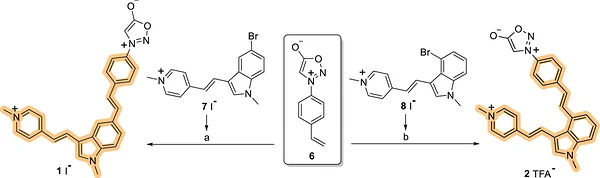
Synthesis of sydnone‐cyanine‐styryl conjugates **1** and **2** from published building blocks **6** [33], **7** [28], and **8** [27]: (a) Pd_2_(dba)_3_, QPhos, Et_3_N, DMF, 80°C, 18 h, 49%; (b) Pd_2_(dba)_3_, QPhos, Et_3_N, DMF, 80°C, 18 h, 45%.

Synthesis of the conjugates **2**–**5** was based on the novel and central sydnone building block **14** extending the aromatic core by naphthalene compared to the previously applied building block **6** (Scheme 2). Its synthesis starts with commercially available 1‐naphthylamine (**9**). Following a published procedure [38], it was iodinated by NIS in DMSO to **10** in a yield of 50%. Naphthylamine **10** [38] was alkylated at its nitrogen by ethyl bromoacetate to ester **11** in a yield of 20%. The ethyl group at the ester function of naphthylamide **11** was then cleaved by aqueous LiOH to give the carboxylic acid **12** in a yield of 95%. With the latter as starting compound, sydnone building block **14** can be built in a two‐step reaction. Firstly, ring closure forms sydnone **13** in a yield of 90% which secondly was coupled by a Stille reaction with tetravinyltin using Pd(PPh_3_)_2_Cl_2_ as catalyst. The building block **14** was obtained in a yield of 72%. The two cyanine styryl building blocks **7** [28] and **8** [27] were finally coupled to the sydnone **14** using a Heck reaction. The target compounds **3** and **4** were obtained in yields of 60% and 45%, respectively. The third sydnone‐dye conjugate **5** was synthesized using the formylated sydnone **15** [33] as building block. It is available in one step from sydnone **6** that was used for the synthesis of sydnones **1** and **2** [33]. Chinolinium **17** is available also in one step from chinoline **16** by methylation [27]. Heck coupling of **15** and **17** finally forms sydnone **5** in 50% yield.

**SCHEME 2:**
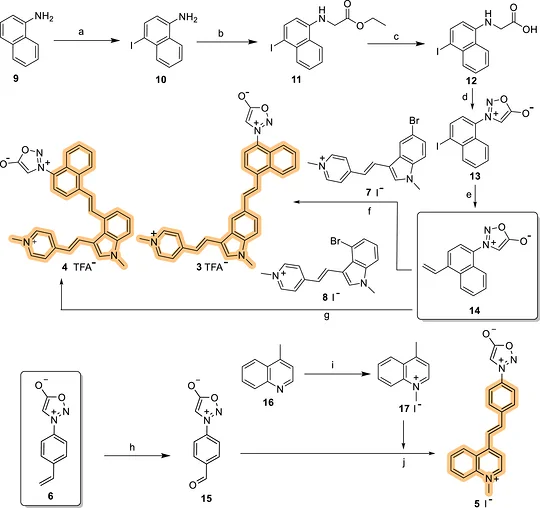
Synthesis of the sydnone‐dye conjugates **3**–**5**: (a) N‐iodosuccinimide, DMSO, r.t., 72 h, 50%; (b) 1) K_2_CO_3_, ethylbromoacetate, DMF, r.t., 16 h, 2) Et_2_NH, DMF, r.t., 1 h, 20%; (c) LiOH, THF:H_2_O = 1:1 (v/v), 0°C, 0.5 h, r.t., 1 h, 95%; (d) 1) tBuONO, THF, r.t., 0.5 h, 2) trifluoroacetic anhydride, THF, r.t., 3 h, 90%; (e) (CH_2_ = CH_2_)_4_Sn, Pd(PPh_3_)_2_Cl_2_, DMF, r.t., 16 h, 72%; (f) Pd_2_(dba)_3_, QPhos, Et_3_N, DMF, 80°C, 18 h, 60%; (g) Pd_2_(dba)_3_, QPhos, Et_3_N, DMF, 80°C, 18 h, 45%; (h) 1) *N*‐methylmorpholine‐*N*‐oxide, OsO_4_, acetone:H_2_O, r.t., 4 h, 2) NaIO_4_, acetone:water, r.t., 6 h, 48%; (i) MeI, acetone, reflux, 2 h, 93%; (j) piperidine, MeOH, 75°C, 24 h, 50%.

BCN is one of the commonly used click partner for bioorthogonal labeling *in vitro* and *in vivo* because it combines ring strain with sufficient stability in biological environments. To investigate the potential of sydnone‐modified dyes **1**–**5** for BCN‐based nucleic acid labeling in vitro, we need a BCN‐modified nucleoside and 2’‐deoxynucleoside as reactive RNA and DNA monomers. In previous studies with tetrazine‐dye conjugates, BCN‐modified 7‐deaza‐2’‐deoxyadenosine **23** showed the highest fluorescence turn‐on effect and fastest reaction kinetics compared to structurally similar BCN‐modified 2’‐deoxyuridines [28]. For the application in RNA, BCN‐modified 7‐deaza‐adenosin **18** was synthesized in a few steps (Scheme 3). 7‐deaza‐7‐iodo‐adenosine (**19**) was synthesized as precursor following the procedure developed by Spitale et al. [39]. Propargyl amine (**20**) was converted into the trifluoroacetamide‐protected linker **21** [40] and attached to iodinated nucleoside **19** in a yield of 70% using a Sonogashira reaction. The synthetic protocol was adapted from the published thymidine [40]. The propargyl linker of nucleoside **22** was deprotected using a concentrated aq. NH_4_OH solution and reacted further without purification. The activated BCN‐NHS ester in DMF was coupled to BCN‐modified **18** and isolated in a yield of 80%. The corresponding BCN‐modified **23** was synthesized according to literature [28].

**SCHEME 3:**
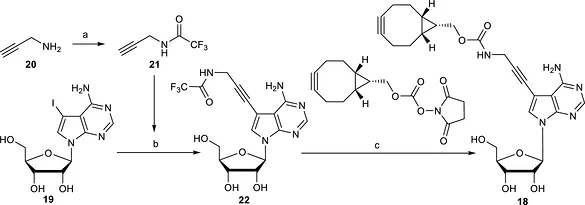
Synthesis of BCN‐modified 7‐deazaadenosine **18**: (a) ethyltrifluoroacetate, MeOH, r.t., 24 h, 72%; (b) Pd(PPh_3_)_4_, CuI, Et_3_N, DMF, r.t., 24 h, 70%; (c) 1) conc. aq. NH_4_OH, r.t., 16 h, 2) BCN‐carbamate‐NHS ester, Et_3_N, DMF, r.t., 16 h, 80%.

### 2.2 Kinetics and Fluorescent Readout of SPSAC *In Vitro*

Product formation and kinetics of SPSAC were studied in vitro using **18** (BCN‐A) and **23** (BCN‐dA) as reactive RNA and DNA monomers, respectively, with sydnone‐dye conjugates **1**–**5**. The progression of SPSAC was characterized by time‐dependent observation of fluorescence readout to obtain information on the reaction progress and to derive second‐order rate constants k_2_ (Figure 2). We evaluated this approach by HPLC analysis of tetrazine‐modified cyanine‐styryl dyes for Diels‐Alder reactions with inverse electron demand [28]. Additionally, we support it by time‐dependent UV/Vis spectroscopy, representatively performed with sydnones **1** and **2** (Figures S37,S38). Following their reaction with **23** elucidated isosbestic points at 293 nm, 344 nm and 435 nm (with **1**) and 344 nm and 491 nm (with **2**), indicating clean conversion of dyes **1** and **2** to products **25** and **27**, respectively, in one step without intermediates. The fluorescence intensity increase was determined by the turn‐on value tov which is the ratio I/I_0_ by the fluorescence intensity I_0_ before and I after completed reaction. Since the changes of fluorescence intensities are solely the result of SPSAC (Figures S39–S49), the exponential fitting of the integrated intensities could be used to derive second‐order rate constants k_2_ (Table 1). All reaction products **24**–**33** were additionally identified by LC‐ESI mass spectrometry (Figures S50–S59). The reactivity of sydnone‐dye conjugates **1**–**5** was studied using nucleoside **18** and 2’‐deoxynucleoside **23**. The rate constants k_2_ are in a range from 0.4±0.05 M^−1^s^−1^ to 7.0±0.3 M^−1^s^−1^, therefore differ by just one magnitude of order. From comparing reactions of **18** with that of **23** it becomes obvious that the 2'‐hydroxyl group of **18** slows down the reactivity with all sydnone dyes **1**–**5**, but overall the differences are smaller than one magnitude of order (factor 1.3 to 4.2). No significant fluorescence turn‐on was observed for the SPSAC of sydnones **1** and **2** with **18** and **23**, tov values do not exceed 2, their products **24**–**27** fluoresce at *λ* = 547 nm and *λ* = 549 nm (Figures S39,S40). Both dyes have therefore no potential for fluorogenic labeling of nucleic acids. This becomes also evident when directly compared with the structurally very similar tetrazine‐dye conjugates previously published (**1** corresponds to tetrazine #3a [28] and **2** corresponds to tetrazine #1 [27]). Their IEDDA reactions with **23** showed tov of 32 and 10, respectively, and rate constants of k_2_ = 137 M^−1^s^−1^ and 500 M^−1^s^−1^, respectively. Sydnones **3** and **4** have an enlarged dye part by the naphthalene that increases the tov values to 3‐4, their products **28**–**31** fluoresce at *λ* = 543 nm and *λ* = 568 nm. The red‐shift of fluorescence observed for the products **30** and **31** can probably be attributed to the special molecular geometry of dye **4** providing the structural prerequisite for stacking interactions between the styryl dye and 7‐deazaadenine. The structurally different and smaller sydnone **5** reacts to products **32** and **33** that show hyposchromically shifted fluorescence at *λ* = 444 nm, smaller Stokes shifts, and most importantly, significant fluorescence turn on. The reaction of **5** with nucleosides **18** and **23** give turn‐on values of 7 and 8, respectively. Interestingly, both products show a broad fluorescence side band at *λ* = 575 nm and therefore a dual fluorescence. In conclusion, only sydnone‐dye conjugates **3**–**5** will be further investigated for labeling of nucleic acids.

**FIGURE 2:**
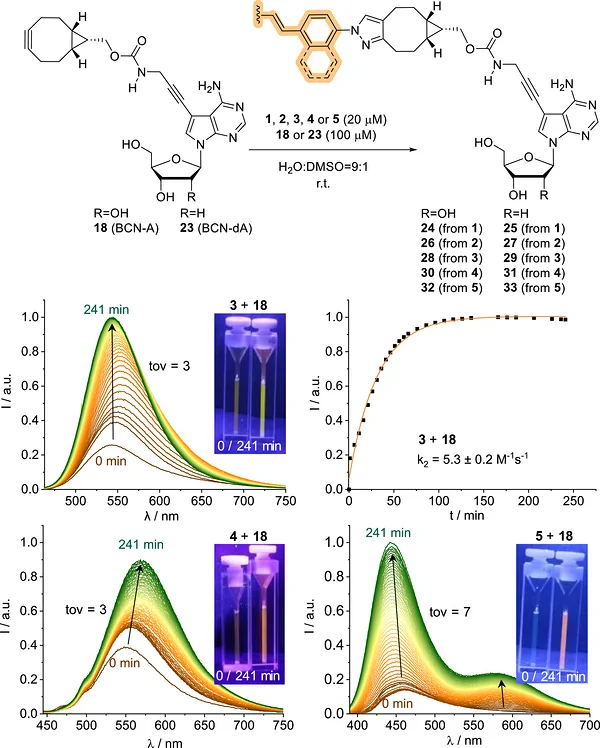
Top: SPSAC of nucleoside **18** and 2’‐deoxynucleoside **23** with sydnone‐dye conjugates **3**–**5**; middle: fluorescence turn‐on (left) and derived kinetics (right) of the reaction of nucleoside **18** with sydnone‐dye conjugate **3**; bottom: fluorescence turn‐on of the reaction of nucleoside **18** with sydnone‐dye conjugate **4** (left) and **5** (right): 20 µM sydnone‐dye conjugate **3**–**5**, 100 µM **18** in H_2_O:DMSO = 99:1 at r.t., *λ*_exc_ = 448 nm (**3**), 424 nm (**4**), 372 nm (**5**). Images (insets) of the cuvettes before and after SPSAC with **3**, **4**, and **5**, excitation by a UV handhelp lamp (254 nm). For sydnone‐conjugates **1** and **2** see Figures S39, S40.

**TABLE 1:** Rate constants k_2_ and fluorescence turn‐on values tov of the SPSACs of nucleoside **18**, 2’‐deoxynucleoside **23**, and **DNA‐BCN** with the sydnone‐dye conjugates **1**–**5**, and optical properties (fluorescence *λ*_max_, Stokes shift Δ*λ* and fluorescence quantum yields *Φ*_F_) of the reaction products **24**–**33** and **DNA‐1**, **DNA‐2**, **DNA‐3**.

Sydnone	BCN	Product	*λ*_max_ (nm)	Δ*λ*	tov	k_2_ (M^−1^s^−1^)
**1**	**18**	**24**	547^a^	118	1	2.2 ± 0.1
	**23**	**25**	547^a^	118	1	2.7 ± 0.1
**2**	**18**	**26**	549^b^	125	1	1.2 ± 0.1
	**23**	**27**	549^b^	125	2	3.6 ± 0.8
**3**	**18**	**28**	543^c^	95	3	5.3 ± 0.2
	**23**	**29**	543^c^	95	4	7.0 ± 0.3
	**DNA‐BCN**	**DNA‐3**	534^c^	86	11	1,500
**4**	**18**	**30**	568^d^	144	3	0.6 ± 0.03
	**23**	**31**	568^d^	144	4	2.5 ± 0.2
	**DNA‐BCN**	**DNA‐4**	564^d^	140	17	2,300
**5**	**18**	**32**	444^e^	72	7	0.4 ± 0.05
	**23**	**33**	444^e^	72	8	1.3 ± 0.1
	**DNA‐BCN**	**DNA‐5**	567 [3]	195	15	100

^a^ λ_exc_ = 437 nm.

^b^ λ_exc_ = 424 nm.

^c^ λ_exc_ = 448 nm.

^d^ λ_exc_ = 424 nm.

^e^ λ_exc_ = 372 nm.

Additional to the monomers, nucleoside **18** and 2’‐deoxynucleoside **23**, we wanted to explore the SPSAC of sydnone‐dye conjugates **3**–**5** using a BCN‐modified oligonucleotide (Figure 3 and Table 1). Sydnones **1** and **2** were omitted in these experiments because they have not shown any fluorescence turn‐on in the previous experiments with the nucleosides. We synthesized **DNA‐BCN** by the published postsynthetic procedure [28] with commercially available BCN‐carbamate‐NHS ester. The reaction products after fluorescent modification by SPSAC, **DNA‐3**, **DNA‐4** and **DNA‐5**, were identified by LC‐ESI mass spectrometry (Figures S60–S62). Over the reaction time, there is a significant fluorescence turn‐on observable with tov values of 11 for **DNA‐3**, and 17 for **DNA‐4**, which is more than magnitude of order fluorescence contrast. Exponential fitting of these fluorescence changes elucidated second order rate constants of k_2_ = 1,500 M^−1^s^−1^ for sydnone‐dye **3** and k_2_ = 2,300 M^−1^s^−1^ for sydnone‐dye **4**. Compared to the reaction with monomers **18** and **23**, we observed with **DNA‐BCN** a significant acceleration of the reaction by factors 215 (with **3**) and 920 (with **4**). To explain this observation, we assume a DNA‐templated pre‐coordination of the sydnone‐dyes **3** and **4**, due to the curved dye structure and the electrostatic attraction of the positively charged dyes by the negatively charged phosphodiester backbone of DNA. This template effect on the cycloaddition has been previously observed with structurally similar tetrazine‐dye conjugates for IEDDA reactions to a significantly higher extent [27, 28]. For those reactions with **DNA‐BCN**, tov values of 115–170 and an acceleration to k_2_ = 63,000‐284,000 M^−1^s^−1^ were obtained. In comparison, sydnone‐dye **5** shows a time‐dependent fluorescence increase over the reaction period with a second‐order rate constant of k_2_ = 100 M^−1^s^−1^, an acceleration by a factor of only 77 compared to the reaction between **6** and **23**. Obviously, the DNA template effect is smaller due to the smaller size and the missing curvature of this dye. Also, the fluorescence turn‐on increased only by tov = 7 for **DNA‐5**. However, the comparison of the fluorescence of product **33** (of **5** with **23**), a significant fluorescence red‐shift of 123 nm is observed. This is an interesting behavior that is potentially useful for metabolic labeling of RNA and DNA: The red‐shift of fluorescence from *λ* = 444 nm for **33** to *λ* = 567 nm for **DNA‐5** provides the potential to distinguish the reaction of the modified nucleosides from the reaction of the metabolically modified DNA simply by the fluorescence color as readout inside cells.

**FIGURE 3:**
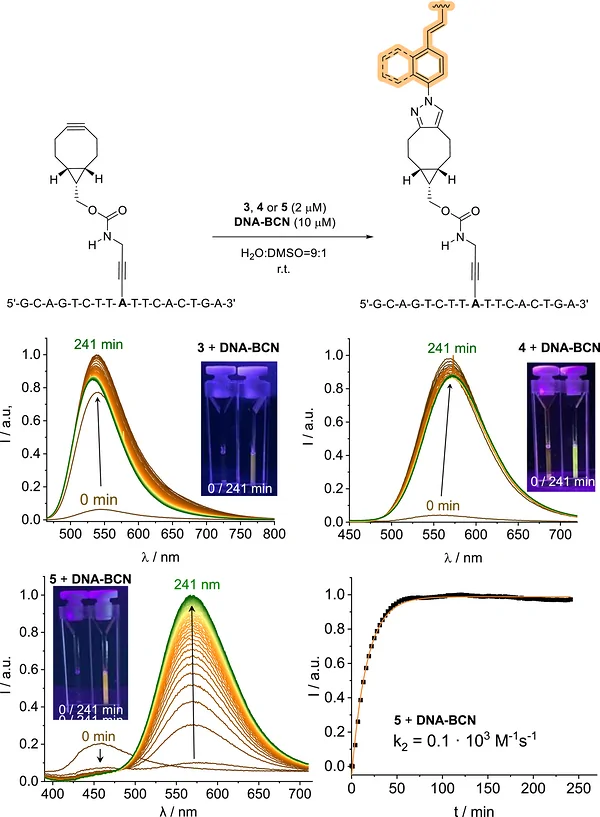
Top: SPSAC of **DNA‐BCN** with sydnon‐dye conjugates **3**–**5**; middle: fluorescence turn‐on of the reaction of **DNA‐BCN** with sydnone‐dye conjugate **3** (left) and **4** (right); bottom: fluorescence turn‐on (left) and derived kinetics (right) of the reaction of **DNA‐BCN** with sydnone‐dye conjugate **5**; 20 µM sydnone‐dye conjugate **3** and **4**, 2 µM sydnone‐dye conjugate **5,** 10 µM **DNA‐BCN** in H_2_O:DMSO = 99:1 at r.t., *λ*_exc_ = 448 nm (**3**), 424 nm (**4**), 372 nm (**5**).

### 2.3 Fluorescent Readout of SPSAC in Cultured Cells

To investigate the biocompatibility and bioorthogonality, fluorescent labeling of DNA by SPSAC was tested using dyes **3**–**5** *in cellulo*. Therefore, HeLa cells were transfected for 24 h with 150 nM of synthetic **DNA‐BCN** using Lipofectamine as transfection reagent. Subsequently, the cells were fixed using 4% paraformaldehyde for 1 h. The fixation was stopped by adding 50.0 mM glycine and 50.0 mM NH_4_Cl for 5 min. Although wash‐free conditions are particularly important for metabolic labeling strategies, they failed for transfected modified DNA. After washing the cells two times, the cells were treated by the respective dyes, 150 nM **3**, 300 nM **4** and 600 nM **5** based on the differing brightness of the dyes, from stock solutions in DMSO. After 2 h, cells were washed. Finally, we imaged the cells via confocal fluorescence microscopy (Figure 4). The click‐products **DNA‐3**, **DNA‐4,** and **DNA‐5** could clearly be visualized in the cytosol. As positive controls, we performed the transfection experiment with in vitro “pre‐clicked” **DNA‐3**, **DNA‐4**, and **DNA‐5** (Figures S63–S65). These HeLa cells showed similar fluorescence in the cytosol which confirmed the successfully transfected click product. As negative controls, we treated the HeLa cells with the dyes **3**–**5** without prior transfection by **DNA‐BCN**. There was only minor fluorescence detectable in those cells. Taken together, we evidenced the successful fluorescence labeling of transfected **DNA‐BCN** by sydnone‐dye conjugates **3**–**5**. Accordingly, fluorogenic SPSAC was proven to be bioorthogonal for DNA labeling. This paves the way to application of sydnone dyes in cell imaging and future metabolic labeling experiments.

**FIGURE 4:**
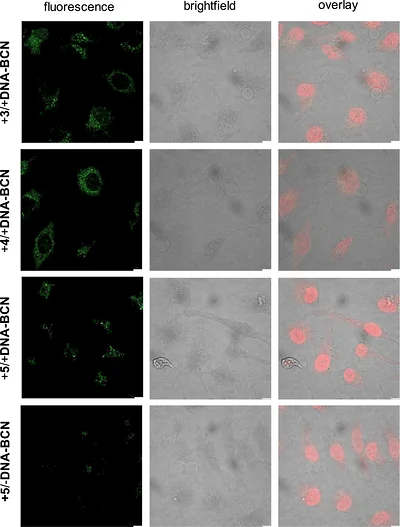
Fluorescence microscopic images of fixed HeLa cells treated for 2 h with sydnones **3**, **4**, or **5**, after transfection with **DNA‐BCN**. Negative control in last lane: HeLa cells without transfected **DNA‐BCN** treated with sydnones **5** for 2 h; **3** and **4**: *λ*_exc_ = 488 nm, *λ*_em_ = 500–600 nm, **5**: *λ*_exc_ = 405 nm, *λ*_em_ = 500–600 nm, Scale bar: 10 µm. Draq5 was added to stain DNA.

## 3 Conclusion

Cyanine‐styryl dyes **1**–**5** conjugated to sydnones were synthesized to combine fluorogenicity with bioorthogonal reactivity for DNA and RNA modification. The synthesis was based on synthetic modules and building blocks using Heck couplings as central conjugation steps. Sydnones **1**–**5** are storable in solid state and are stable in aqueous solution and puffer allowing incubations with cells. We have applied SPSAC chemistry with BCN as reactive group, characterized the reaction kinetics and fluorogenicity in vitro, and evidenced bioorthogonality by experiments *in cellulo*. Due to their significant Stokes shifts of 72–195 nm, excitation and emission are well separated which is an advantage for fluorescent cell imaging. Reaction kinetics and fluorogenicity of dyes **1**–**5** were investigated by spectroscopic means using BCN‐modified 7‐deazadenosine **18** as RNA monomer, 7‐deaza‐2’‐deoxyadenosine **23** as DNA monomer and **DNA‐BCN** as oligomer. For monomers **18** and **23**, the determined second‐order rate constants k_2_ were found in a range from 0.4±0.05 M^−1^s^−1^ to 7.0±0.3 M^−1^s^−1^. No significant fluorescence turn‐on was observed during the SPSAC of sydnones **1** and **2** with **18** and **23**. Both dyes have therefore no potential for fluorogenic labeling of nucleic acids. Sydnones **3** and **4** have an enlarged dye part by the naphthalene that increases the fluorescence turn‐on to 3–4 and bathochromically shifts the fluorescence. The structurally different and smaller sydnone **5** reacts to products not only with fluorescence turn‐on of 7–8 but also with a hyposchromic shift. Similar experiments in vitro with the BCN‐modified oligonucleotide **DNA‐BCN** showed a significant fluorescence turn‐on of 11–17 for dyes **3** and **4**, which is more than magnitude of order fluorescence contrast. The determined second order rate constants of k_2_ = 1,500 M^−1^s^−1^ and k_2_ = 2,300 M^−1^s^−1^ evidenced a significant acceleration of the SPSAC which can be rationalized by the DNA template effect due to the curved dye structure of the dyes and the electrostatic interaction with DNA. Sydnone‐dye **5** shows a time‐dependent fluorescence contrast of 6 and rate constant of k_2_ = 100 M^−1^s^−1^. Obviously, the DNA template effect is smaller due to the smaller size and the missing curvature of this dye. More importantly, the fluorescence of the SPSAC product of dye **5** with **DNA‐BCN** shows a red‐shift of 123 nm. This is a potentially useful property for metabolic labeling of RNA and DNA because it helps to distinguish the reaction of the modified nucleosides from the reaction of the metabolically modified DNA simply by the fluorescence color as readout inside cells. Metabolic labeling of DNA and RNA is not anymore limited to small groups as nucleoside modifications and bulky bioorthogonally reactive groups can be used, too. We showed recently that BCN‐modified and trans‐cyclooctene‐modified nucleosides can be indeed applied for metabolic labeling of RNA by circumventing the problem of cellular kinase selectivity using the concept of pronucleotides [41]. Furthermore, we evidenced bioorthogonality by fluorescence labeling of transfected **DNA‐BCN** *in cellulo* using sydnone‐dye conjugates **3**–**5**. The dyes are cell permeable and can undergo SPSAC reactions with BCN‐modified DNA in the environment of HeLa cells. In conclusion, sydnone‐dye conjugates **3**–**5** have promising properties for fluorescent cell imaging in general and future metabolic labeling experiments in particular.

## 4 Experimental Section

### 4.1 Materials and Methods

The reagents used for synthesis were purchased from ABCR, ALFA AESAR, CARBOLUTIONS, FLUKA, MERCK, SIGMA ALDRICH, BLDpharm, TCI and VWR and used without further purification. Solvents were purchased from SIGMA ALDRICH, ACROS ORGANICS and FISCHER, which were at least quality level *pro analysi* (*p.A*.). Dry solvents were also purchased, stored and used under argon. Technical solvents were used for work‐up and for chromatographic purification, unless otherwise specified. Double deionized water (*Milli‐Q Direct 8/16* system from Merck *Millipore*) and high purity solvents (HPLC *grade*) were used for HPLC separations. Buffers used were filtered before. Deuterated solvents were bought from EURISO‐TOP. Air‐ and water‐sensitive reactions were carried out using Schlenk technique under an argon atmosphere (Argon 5.0, Air Liquide, 99.999% purity). The glass equipment was previously heated several times in a high vacuum and flushed with argon several times. Liquids were taken utilizing plastic syringes and cannulas or pipettes of the Eppendorf brand. If required, the solvents were degassed by passing in argon or by applying the *freeze‐pump‐thaw* method. Solids were transferred using weighing paper. For reactions at low temperatures, the following cooling mixtures were used: 0°C: ice in water, ‐21°C: NaCl in ice, ‐78°C: dry ice in isopropanol. Solvents were removed under reduced pressure on a rotary evaporator at a water bath temperature of 45°C. For thin‐layer chromatography, aluminum plates coated with silica gel (silica gel 60, F_254_, layer thickness 0.250 mm) from MERCK were used. For the detection of the spot samples, UV‐active samples were detected with UV light, by fluorescence quenching at λ = 254 nm or fluorescence excitation at λ = 366 nm. UV‐inactive samples were stained with potassium permanganate solution (1.50 g KMnO_4_, 10.0 g K_2_CO_3_, 1.25 mL 10% NaOH solution, 200 mL H_2_O). Saccharides were stained with a solution of sulfuric acid in methanol (5% H_2_SO_4_) and amines with a ninhydrin solution (3.00 g ninhydrin in 200 mL EtOH). Column chromatography was used to purify the crude products. Silica gel 60 (pore size 60 Å, particle size 40–63 µm) from SIGMA ALDRICH served as stationary phase. The raw products were adsorbed on *Celite S*, dry loaded or applied as a solution. Elution was carried out with overpressure generated by a hand pump. Reversed‐phase high‐performance liquid chromatography (HPLC) of oligonucleotides was carried out on a THERMO SCIENTIFIC HPLC instrument (*Dionex UltiMate3000* autosampler, pump module, column oven, multidiode array, RS fluorescence detector, fraction collector, *Chromeleon 7* software). The separation was performed on a semi‐preparative column (*VDSpehr OptiBio PUR 300* S18‐SE column (250 × 10 mm, 5 µm)). The analytes were applied to the column with an injection volume of 300 µL and a flow rate of 3.50 mL/min, unless otherwise stated. The column was equilibrated with 0.05 M ammonium acetate buffer in water and eluted with various gradients of acetonitrile. The signals were measured using a UV diode array detector at wavelengths of 260 nm, 290 nm and the characteristic absorption of the respective artificial building blocks. For the monitoring of the SPSAC reactions via HPLC‐MS an analytical column (*VDSpehr OptiBio PUR 300* S18‐SE column (250 × 4.6 mm, 5 µm)) was used. A solvent gradient of acetonitrile and double‐distilled water was used. The analytes were applied on the column with an injection volume of 20 µL and a flow rate of 1.00 mL/min, unless otherwise stated. For the analytical runs with the sydnone fluorophores 0.1% TFA was additionally added to the solvents. The signals were measured using a UV diode array detector at wavelengths of 260 , 290 nm and the characteristic absorption of the respective artificial building blocks. Nuclear magnetic resonance analyses were performed in deuterated solvents on a BRUKER *Advance 400* (400 MHz ^1^H ‐NMR or 101 MHz ^13^C‐NMR) or BRUKER *Advance 500* (500 MHz ^1^H‐NMR or 126 MHz ^13^C‐NMR). For the measurement, 5–10 mg of the sample were dissolved in approx. 0.5 mL of deuterated solvent in a measuring tube with a diameter of 5 mm. The deuterated solvents employed were [d6]‐DMSO, [d1]‐CDCl_3_ and [d4]‐MeOD. The measured chemical shifts (*δ*) were given in *parts per million* (ppm) and refer to tertramethylsilane as 0.0 ppm. The spectrum was calibrated to the signals of the not fully deuterated solvent used; [*d6*]‐DMSO: ^1^H‐NMR: δ = 2.50 ppm and ^13^C‐NMR: δ = 39.52 ppm, [*d1*]‐CDCl_3_: ^1^H‐NMR: δ = 7.26 ppm and ^13^C‐NMR: *δ* = 77.16 ppm, [*d4*]‐ MeOD: ^1^H‐NMR: δ  = 3.31 ppm and ^13^C‐NMR: δ = 49.00 ppm. The measured coupling constants *J* were given in Hertz (Hz). The multiplicities of the signals were labelled with the abbreviations s (singlet), d (doublet), dd (doublet of doublet), t (triplet), q (quartet), quin (quintet) and m (multiplet) and br (broadened signal). The spectra were evaluated with the help of the software MESTRENOVA. The chemical shifts of known solvents were taken from known literature sources for example Fulmer et al. [41]. MALDI‐TOF measurements were carried out on an *AXIMA Confidence* spectrometer from *SHIMADZU*. A standard plate made of stainless steel from the company Bruker with 386 application points was used as the target. A matrix of 3‐ hydroxypicolinic acid (saturated solution in acetonitrile/water = 1:1) and diammonium hydrogen citrate (0.44 M in water) in a 9:1 mixture was used for DNA mass measurements. The signals were reported by their mass to charge ratio (*m*/*z*). Small molecule masses were recorded on a Q Exactive Orbitrap and an LTQ Orbitrap XL from THERMO FISHER SCIENTIFIC using electron spray ionization (ESI). The signals were given in mass/charge ration (*m*/*z*) and the molecular ion was shown as [M]^+^ or in protonated form as [M+H]^+^ or [M‐H]^+^ in positive mode. The examination of the synthesized substances was carried out in quartz glass cuvettes from STARNA path length 1 cm, sample volume 1 mL or 0.5 mL) at a temperature of 20°C. All spectra were baseline‐corrected using a blank. The UV/Vis absorption spectra were recorded on a Cary3500 spectrometer from AGILANT with Peltier element from AGILENT. The samples were prepared using 1 cm quartz glass cuvettes (STARNA) with a volume of 1 mL at 25°C. To determine the extinction coefficients, different concentrations were measured and applied to Lambert–Beer's law. Fluorescence spectra were recorded on a *Fluoromax‐4* spectrofluorometer from HORIBA‐SCIENTIFIC with a Peltier element from Horiba Scientific at 20°C. The spectrometer was calibrated using the Raman scatter of water as a reference to ensure accurate wavelength measurement.

### 4.2 Synthesis of **1**

In a heated 10 mL crimp vial under argon, 0.100 g **7** [28] (0.220 mmol, 1.00 eq.), 0.010 g Pd_2_(dba)_3_ (0.010 mmol, 0.05 eq.), 0.031 g Qphos (0.040 mmol, 0.20 eq.) and 0.083 g **6** [33] (0.440 mmol, 2.00 eq.) dried under high vacuum. The solids were then dissolved in 2.00 mL dry DMF, and 0.15 mL triethylamine (0.111 g, 1.10 mmol, 5.00 eq.) was added. The reaction was stirred for 18 h in a metal heating block at 80°C. After the solution had cooled to room temperature Et_2_O was added until no further precipitate was formed. The suspension was transferred into reaction tubes. The solution was centrifuged and the supernatant was pipetted off and discarded. Subsequently, Et_2_O was added and the suspension was vortexed and centrifuged again in the next step and the supernatant discarded. This was carried out two more times. The residue was dried in high vacuum. The product was obtained as a brown solid in a yield of 0.062 g (0.110 mmol, 49%). ^1^H‐NMR (400 MHz, DMSO‐*d_6_*): δ (ppm) = 8.72 (d, *J* = 6.4 Hz, 2H, arom.), 8.42 (s, 1H, arom.), 8.24 (d, *J* = 16.2 Hz, 1H, R‐CH = CH‐R), 8.17 (d, *J* = 6.5 Hz, 2H, arom.), 8.02 – 7.96 (m, 3H, arom., R‐CH = CH‐R), 7.93 (d, *J* = 8.6 Hz, 2H, arom.), 7.82 (s, 1H, arom.), 7.73 – 7.61 (m, 3H, arom.), 7.48 (d, *J* = 16.5 Hz, 1H, R‐CH = CH‐R), 7.35 (d, *J* = 16.2 Hz, 1H, R‐CH = CH‐R), 4.19 (s, 3H, R‐C*H_3_*), 3.91 (s, 3H, R‐C*H_3_*). ^13^C‐NMR (126 MHz, DMSO‐*d_6_*): δ (ppm) = 168.5, 154.0, 144.3, 141.7, 138.1, 136.1, 135.3, 132.8, 130.2, 127.4, 125.8, 124.8, 121.8, 119.8, 117.2, 113.0, 111.5, 94.5, 46.3, 33.3. HR‐MS (ESI): *m*/*z* (calculated for C_27_H_23_N_4_O_2_^+^): 435.1816 [M]^+^; found: 435.1811 [M]^+^.

### 4.3 Synthesis of **2**

In a heated 10 mL crimp vial under argon, 0.100 g **8** [27] (0.220 mmol, 1.00 eq.), 0.010 g Pd_2_(dba)_3_ (0.010 mmol, 0.05 eq.), 0.031 g Qphos (0.040 mmol, 0.20 eq.) and 0.083 g **6** [33] (0.440 mmol, 2.00 eq.) dried under high vacuum. The solids were then dissolved with 2.00 mL dry DMF and 0.15 mL Et_3_N (0.111 g, 1.10 mmol, 5.00 eq.) was added. The reaction was stirred for 18 h in a metal heating block at 80°C. After the solution had cooled to room temperature Et_2_O was added until no further precipitate was formed. The suspension was transferred into reaction tubes. The solution was centrifuged and the supernatant was pipetted off and discarded. Subsequently, Et_2_O was added and the suspension was vortexed and centrifuged again in the next step and the supernatant discarded. This was carried out two more times. The residue was dried in high vacuum and purified via reversed‐phase column chromatography. The product was obtained as a brown solid in a yield of 0.056 g (0.099 mmol, 45%). ^1^H‐NMR (500 MHz, DMSO‐*d_6_*): δ (ppm) = 8.68 (d, *J* = 6.8 Hz, 2H, arom.), 8.41 (d, *J* = 15.8 Hz, 1H, R‐CH = CH‐R), 8.26 (d, *J* = 16.1 Hz, 1H, R‐CH = CH‐R), 8.21 (s, 1H, arom.), 8.04 (d, *J* = 8.7 Hz, 2H, arom.), 7.98 (t, *J* = 7.9 Hz, 4H, arom.), 7.82 (s, 1H, arom.), 7.57 (d, *J* = 8.1 Hz, 1H, arom.), 7.51 (d, *J* = 7.4 Hz, 1H, arom.), 7.33 (t, *J* = 7.8 Hz, 1H, arom.), 7.28 (d, *J* = 16.1 Hz, 1H, R‐CH = CH‐R), 7.14 – 7.09 (m, 1H, R‐CH = CH‐R), 4.17 (s, 3H, R‐C*H_3_*), 3.91 (s, 3H, R‐C*H_3_*). ^13^C‐NMR (126 MHz, DMSO‐*d_6_*): δ (ppm) = 168.6, 153.3, 144.5, 141.3, 138.0, 136.0, 133.2, 132.6, 130.8, 130.4, 129.1, 128.0, 123.9, 122.8, 121.8, 119.8, 118.6, 113.2, 111.2, 94.6, 46.5, 33.4. HR‐MS (ESI): *m*/*z* (calculated for C_27_H_23_N_4_O_2_^+^): 435.1816 [M]^+^; found: 435.1811 [M]^+^

### 4.4 Synthesis of **11**

In a heated Schlenk flask under argon 6.50 g **10** [38] (24.2 mmol, 1.00 eq.) was dissolved in 45 mL DMF. 4.01 g K_2_CO_3_ (28.9 mmol, 1.20 eq.) was added. 3.21 mL ethyl bromoacetate (4.84 g, 28.9 mmol, 1.20 eq.) was then added in portions for 10 min. The reaction mixture was allowed to stir overnight at room temperature. 5.25 mL diethylamine (3.71 g, 50.7 mmol, 2.10 eq.) was added in portions for 5 min and the reaction solution was allowed to stir for 1 h. The solid was filtered off. The solvent of the filtrate was removed. The residue was purified by automated column chromatography (cyclohexane:EtOAc 0–40 %). The product was obtained as a red brown solid with a yield of 3.20 g (9.01 mmol, 37%). ^1^H‐NMR (400 MHz, DMSO‐*d_6_*): δ (ppm) = 8.15 (d, *J* = 8.3 Hz, 1H, arom.), 7.89 (dd, *J* = 8.0, 1.3 Hz, 1H, arom.), 7.82 (d, *J* = 8.2 Hz, 1H, arom.), 7.60 (t, *J* = 7.2, 1H, arom.), 7.52 (t, *J* = 7.1 Hz, 1H, arom.), 6.94 (t, 1H, R‐NH‐R), 6.20 (d, *J* = 8.2 Hz, 1H, arom.), 4.13 (q, *J* = 7.1 Hz, 2H, R‐CH_2_‐CH_3_), 4.08 (d, *J* = 6.2 Hz, 2H, R‐NH‐CH_2_‐R), 1.20 (t, *J* = 7.1 Hz, 3H, R‐CH_2_‐CH_3_). ^13^C‐NMR (126 MHz, DMSO‐*d_6_*): δ (ppm) = 170.8, 144.6, 137.6, 133.7, 131.6, 127.8, 125.2, 124.1, 122.2, 105.3, 82.4, 60.4, 44.8, 14.1. HR‐MS (ESI): *m*/*z* (calculated for C_14_H_15_INO_2_^+^): 356.0142 [M+H]^+^; found: 356.0140 [M+H]^+^.

### 4.5 Synthesis of **12**

In a Schlenk flask under argon, 4.48 g **11** (12.6 mmol, 1.00 eq.) was placed and dissolved in 16 mL THF. The solution was cooled to 0°C with an ice bath. Meanwhile, 0.604 g LiOH (25.2 mmol, 2.00 eq.) was dissolved in 16 mL H_2_O and then added to the reaction solution. The solution was allowed to stir at 0°C for 1 h and then at room temperature for 1 h. The THF was evaporated off. A pH of 2 was then adjusted with HCl (2 M) and the resulting precipitate was filtered off and washed with a small amount of water. The residue was dried under reduced pressure. The product was obtained as a light violet solid in a yield of 3.99 g (12.2 mmol, 97%). ^1^H‐NMR (400 MHz, DMSO‐*d_6_*): δ (ppm) = 12.66 (s, 1H, R‐COOH), 8.13 (d, *J* = 8.3 Hz, 1H, arom.), 7.89 (d, *J* = 8.3 Hz, 1H, arom.), 7.82 (d, *J* = 8.2 Hz, 1H, arom.), 7.59 (t, *J* = 7.3, 1H, arom.), 7.51 (t, *J* = 7.6 Hz, 1H, arom.), 6.83 (s, 1H, R‐NH‐R), 6.21 (d, *J* = 8.2 Hz, 1H, arom.), 3.98 (s, 2H, R‐NH‐CH_2_‐R). ^13^C‐NMR (126 MHz, DMSO‐*d_6_*): δ (ppm) = 172.2, 144.8, 137.7, 133.7, 131.5, 127.7, 125.1, 124.1, 122.2, 105.3, 82.1, 44.7. HR‐MS (ESI): *m*/*z* (calculated for C_12_H_11_INO_2_^+^): 327.9829 [M+H]^+^; found: 327.9827 [M+H]^+^.

### 4.6 Synthesis of **13**

In a heated Schlenk flask under argon, 3.99 g **12** (12.2 mmol, 1.00 eq.) was introduced and dissolved in 55 mL THF. Slowly 1.60 mL tert‐butyl nitrite (1.38 g, 13.4 mmol, 1.10 eq.) was added to the reaction solution over 10 min. The reaction mixture was allowed to stir for 1 h at room temperature. 1.87 mL Trifluoroacetic anhydride (2.82 g, 13.4 mmol, 1.10 eq.) was then added over 10 min and the reaction solution was allowed to stir at room temperature for 1 h. EtOAc was added and the solution was quenched with saturated sodium bicarbonate solution. The aqueous phase was extracted three times with EtOAc. The combined organic phases were dried over sodium sulfate and the solvent was removed. The crude product was purified by automated column chromatography (CH_2_Cl_2_:MeOH 0%–20%). The product was obtained as a red‐brown solid in a yield of 3.42 g (10.1 mmol, 83%). ^1^H‐NMR (400 MHz, CDCl_3_‐*d_1_*): δ (ppm) = 8.28 (d, *J* = 7.9 Hz, 2H, arom.), 7.81 – 7.66 (m, 3H, arom.), 7.42 (d, *J* = 7.8 Hz, 1H, arom.), 6.68 (s, 1H, sydnone core). ^13^C‐NMR (126 MHz, CDCl_3_‐*d_1_*): δ (ppm) = 168.7, 136.5, 135.3, 133.5, 132.1, 130.1, 129.9, 127.3, 124.0, 105.5, 98.6, 77.4. HR‐MS (ESI): *m*/*z* (calculated for C_12_H_8_IN_2_O_2_^+^): 338.9625 [M+H]^+^; found: 338.9624 [M+H]^+^.

### 4.7 Synthesis of **14**

In a heated Schlenk flask under argon, 1.02 g **13** (3.00 mmol, 1.00 eq.) was introduced and dissolved in 70 mL DMF. To the reaction solution, 0.382 g LiCl (9.01 mmol, 3.00 eq.), 0.105 g Pd(PPh_3_)_2_Cl_2_ (0.15 mmol, 0.05 eq.) and 0.60 mL tetravinyltin (0.750 g, 3.31 mmol, 1.10 eq.) were added. The reaction mixture was allowed to stir overnight at 65°C. The solvent was then removed. The residue was dissolved in CH_2_Cl_2_ and 1 M NaOH solution and allowed to stir for 1 h. The aqueous phase was extracted three times with CH_2_Cl_2_. The combined organic phases were dried over Na_2_SO_4_. The solvent was removed. The crude product was purified by automated column chromatography (CH_2_Cl_2_:MeOH 0–10%). The product was obtained as an orange solid in a yield of 0.715 g (3.00 mmol, 100%). ^1^H‐NMR (400 MHz, CDCl_3_‐*d_1_*): δ (ppm) = 8.29 – 8.21 (m, 1H, arom.), 7.78 – 7.73 (m, 1H, arom.), 7.72 (s, 1H, arom.), 7.69 (tt, *J* = 5.6, 3.0 Hz, 3H, arom.), 7.49 (dd, *J* = 17.3, 11.0 Hz, 1H, R‐CH = CH_2_), 7.47 (d, *J* = 11.0 Hz, 1H, arom.), 6.67 (s, 1H, sydnone core), 5.91 (dd, *J* = 17.3, 0.9 Hz, 1H, R‐CH = CH_2_), 5.69 (dd, *J* = 11.0, 0.9 Hz, 1H, R‐CH = CH_2_). ^13^C‐NMR (126 MHz, CDCl_3_‐*d_1_*): δ (ppm) = 168.9, 141.0, 133.2, 131.8, 129.0, 128.1, 127.1, 125.0, 123.4, 122.7, 121.5, 120.7, 98.6, 77.4.

HR‐MS (ESI): *m*/*z* (calculated for C_14_H_11_N_2_O_2_^+^): 239.0815 [M+H]^+^; found: 239.0825 [M+H]^+^.

### 4.8 Synthesis of **3**

In a heated 10 mL crimp vial under argon, 0.100 g **7** [28] (0.220 mmol, 1.00 eq.), 0.010 g Pd_2_(dba)_3_ (0.010 mmol, 0.05 eq.), 0.031 g Qphos (0.040 mmol, 0.20 eq.) and 0.105 g **14** (0.440 mmol, 2.00 eq.) dried under high vacuum. The solids were then dissolved with 2.00 mL dry DMF and 0.15 mL Et_3_N (0.111 g, 1.10 mmol, 5.00 eq.) was added. The reaction was stirred in a metal heating block at 80°C for 18 h. After the solution had cooled to room temperature, Et_2_O was added until no further precipitate was formed. The suspension was transferred into reaction tubes. The solution was centrifuged and the supernatant was pipetted off and discarded. Subsequently, Et_2_O was added and the suspension was vortexed and centrifuged again in the next step and the supernatant discarded. This was carried out two more times. The residue was dried in high vacuum and purified via reversed‐phase column chromatography. The product was obtained as a brown solid in a yield of 0.079 g (0.132 mmol, 60%). ^1^H‐NMR (500 MHz, DMSO‐*d_6_*): δ (ppm) = 8.76 – 8.70 (m, 3H, arom.), 8.50 (s, 1H, arom.), 8.24 (dd, *J* = 16.1, 12.6 Hz, 2H, R‐CH = CH‐R, arom.), 8.14 (dd, *J* = 14.5, 7.4 Hz, 3H, R‐CH = CH‐R, arom.), 8.06 (d, *J* = 7.8 Hz, 1H, arom.), 8.00 (s, 1H, arom.), 7.94 (d, *J* = 8.6 Hz, 1H, arom.), 7.82 (dtd, *J* = 16.0, 8.0, 3.8 Hz, 3H, R‐CH = CH‐R, arom.), 7.74 (s, 1H, arom.), 7.70 – 7.63 (m, 2H, arom.), 7.38 (d, *J* = 16.2 Hz, 1H, R‐CH = CH‐R), 4.19 (s, 3H, R‐C*H_3_*), 3.93 (s, 3H, R‐C*H_3_*). ^13^C‐NMR (126 MHz, DMSO‐*d_6_*): δ (ppm) = 168.4, 157.8, 157.6, 154.0, 144.3, 139.7, 138.1, 136.0, 135.6, 135.2, 131.0, 130.5, 129.7, 128.9, 127.8, 126.9, 125.8, 125.0, 124.2, 122.2, 121.8, 121.7, 117.3, 113.1, 111.5, 99.8, 46.3, 33.3. HR‐MS (ESI): *m*/*z* (calculated for C_31_H_25_N_4_O_2_^+^): 485.1972 [M]^+^; found: 485.1968 [M]^+^.

### 4.9 Synthesis of **4**

In a heated 10 mL crimp vial under argon, 0.100 g **8** [27] (0.220 mmol, 1.00 eq.), 0.010 g Pd_2_(dba)_3_ (0.010 mmol, 0.05 eq.), 0.031 g Qphos (0.040 mmol, 0.20 eq.) and 0.105 g **14** (0.440 mmol, 2.00 eq.) dried under high vacuum. The solids were then dissolved with 2.00 mL dry DMF and 0.15 mL Et_3_N (0.111 g, 1.10 mmol, 5.00 eq.) was added. The reaction was stirred in a metal heating block at 80°C for 18 h. After the solution had cooled to room temperature, Et_2_O was added until no further precipitate was formed. The suspension was transferred into reaction tubes. The solution was centrifuged and the supernatant was pipetted off and discarded. Subsequently, Et_2_O was added and the suspension was vortexed and centrifuged again in the next step and the supernatant discarded. This was carried out two more times. The residue was dried in high vacuum and purified via reversed‐phase column chromatography. The product was obtained as a brown solid in a yield of 0.059 g (0.099 mmol, 45%). ^1^H‐NMR (500 MHz, DMSO‐*d_6_*): δ (ppm) = 8.70 – 8.63 (m, 3H, arom.), 8.48 (d, *J* = 15.9 Hz, 1H, R‐CH = CH‐R), 8.25 (dd, *J* = 22.0, 7.1 Hz, 3H, R‐CH = CH‐R, arom.), 8.12 – 8.05 (m, 2H, R‐CH = CH‐R, arom.), 8.02 (d, *J* = 6.9 Hz, 2H, arom.), 7.87 – 7.83 (m, 1H, arom.), 7.80 (d, *J* = 7.5 Hz, 1H, arom.), 7.79 – 7.74 (m, 3H, R‐CH = CH‐R, arom.), 7.60 (d, *J* = 8.2 Hz, 1H, arom.), 7.39 (t, *J* = 7.9 Hz, 1H, arom.), 7.13 (d, *J* = 15.9 Hz, 1H, R‐CH = CH‐R), 4.17 (s, 3H, R‐C*H_3_*), 3.94 (s, 3H, R‐C*H_3_*). ^13^C‐NMR (126 MHz, DMSO‐*d_6_*): δ (ppm) = 168.4, 157.7, 157.5, 153.2, 144.4, 139.3, 138.1, 136.0, 132.8, 132.6, 131.0, 130.8, 130.0, 129.0, 128.0, 126.9, 125.9, 124.8, 124.1, 123.9, 122.7, 122.5, 122.0, 121.7, 120.2, 118.8, 113.2, 111.2, 99.9, 46.4, 33.4. HR‐MS (ESI): *m*/*z* (calculated for C_31_H_25_N_4_O_2_^+^): 485.1972 [M]^+^; found: 485.1969 [M]^+^.

### 4.10 Synthesis of **5**

In accordance with a literature prescription to a solution of 0.088 g **17** [28] (0.310 mmol, 1.00 eq.) and 0.065 g **15** [25] (0.340 mmol, 1.10 eq.) in 8 mL MeOH was added three drops of piperidine. The mixture was stirred at 75°C for 24 h. Et_2_O was added and the mixture was cooled to 0°C and stirred for 1 h, after which the precipitate was filtered. The precipitate was dried under reduced pressure. The product was obtained as a dark violet solid in a yield of 0.071 g (0.155 mmol, 50%) ^1^H‐NMR (400 MHz, DMSO‐*d_6_*): δ (ppm) = 9.45 (d, *J* = 6.4 Hz, 1H, arom.), 9.09 (dd, *J* = 8.8, 1.3 Hz, 3H, arom.), 8.59 – 8.47 (m, 3H, arom.), 8.35 – 8.29 (m, 3H, arom.), 8.23 (d, *J* = 16.1 Hz, 1H, R‐CH = CH‐R), 8.16 – 8.09 (m, 3H, arom), 7.90 (s, 1H, sydnone core), 4.59 (s, 3H, N‐C*H_3_*). ^13^C‐NMR (101 MHz, DMSO‐*d_6_*): δ (ppm) = 168.5, 151.9, 148.6, 140.2, 139.2, 138.8, 135.2, 130.1 (2C), 129.6, 129.5, 126.60, 126.56, 123.1, 122.0 (2C), 119.5, 117.1, 94.9, 45.0. HR‐MS (ESI): *m*/*z* (calculated for C_20_H_16_N_3_O_2_^+^): 330.1237 [M]^+^; found: 330.1230 [M]^+^.

### 4.11 Synthesis of **22**

In a heated round bottom flask under argon atmosphere, 1.88 g **21** [40] (4.78 mmol, 1.00 eq.), 2.17 g **19** [39] (14.3 mmol, 3.00 eq.), 0.200 g CuI (1.05 mmol, 0.22 eq.) and 1.33 mL Et_3_N (0.968 g, 9.56 mmol, 2.00 eq.) were dissolved in 30 mL dry DMF. The solution was degassed and then 0.553 g Pd(PPh_3_)_4_ (0.48 mmol, 0.10 eq.) was added. The reaction mixture was stirred at room temperature for 24 h and the solvent was removed under reduced pressure. The residue was purified by column chromatography (CH_2_Cl_2_:MeOH 10 – 50%). The product was obtained as a light brown solid in a yield of 1.39 g (3.35 mmol, 70%). ^1^H‐NMR (400 MHz, DMSO‐*d_6_*): δ (ppm) = 10.1 (t, *J* = 5.5 Hz, 1H, N*H*), 8.11 (s, 1H, H‐2), 7.77 (s, 1H, H‐8), 6.84 (br s, 2H, N*H_2_*), 6.01 (d, *J* = 6.1 Hz, 1H, H‐1’), 5.33 (d, *J* = 6.3 Hz, 1H, 3’‐OH), 5.19 (t, *J* = 5.6 Hz, 1H, 5’‐OH), 5.11 (d, *J* = 4.8 Hz, 1H, 2’‐OH), 4.36 (q, *J* = 6.1 Hz, 1H, H‐3’), 4.31 (d, *J* = 5.4 Hz, 2H, C*H_2_*), 4.10 – 4.03 (m, 1H, H‐4’), 3.89 (q, *J* = 3.6 Hz, 1H, H‐2’), 3.66 – 3.58 (m, 1H, H‐5_a_’), 3.53 (ddd, *J* = 11.9, 6.1, 3.6 Hz, 1H, H‐5_b_’). ^13^C‐NMR (126 MHz, DMSO‐*d_6_*): δ (ppm) = 157.5, 152.7, 149.6, 127.0, 112.2, 102.3, 94.1, 91.0, 87.3, 86.8, 85.2, 76.1, 74.0, 70.5, 61.5, 29.9. HR‐MS (ESI): *m*/*z* (calculated for C_16_H_17_F_3_N_5_O_5_^+^): 416.1176 [M+H]^+^; found: 416.1172 [M+H]^+^.

### 4.12 Synthesis of **18**

In a heated flask under argon atmosphere, 0.070 g **22** (0.25 mmol, 1.00 eq.) was dissolved in 3 mL of concentrate q. NH_4_OH solution and stirred overnight at room temperature. The solvent was removed under reduced pressure and the residue was dried in a high vacuum. The product was obtained as a brown solid with a quantitative yield. In a heated round bottom flask under argon atmosphere, 0.046 g of the residue (0.14 mmol, 1.00 eq.), was dissolved in 5 mL dry DMF. Then 0.08 mL triethylamine (0.058 g, 0.58 mmol, 4.00 eq.) and 0.063 g BCN‐carbamate‐NHS‐ester (0.22 mmol, 1.50 eq.) were added, and the reaction mixture was stirred for 16 h at room temperature. The solvent was then removed under reduced pressure. The residue was dissolved in 25 mL of methanol, approx. 50 mg of Amberlite IRA 402 bicarbonate was added and stirred for 30 min. The solid was filtered off and the solvent was removed under reduced pressure. The crude product was purified by column chromatography (SiO_2_, CH_2_Cl_2_:MeOH 0%– 15%). The product was obtained as a colorless solid in a yield of 0.056 g (0.11 mmol, 80%). ^1^H‐NMR (400 MHz, DMSO‐*d_6_*): δ = 8.10 (s, 1H, H‐2), 7.71 (m, 2H, H‐8, N*H*), 6.00 (d, *J* = 6.0 Hz, 1H, H‐1’), 5.32 (d, *J* = 6.3 Hz, 1H, 3’‐OH), 5.20 (t, *J* = 5.5 Hz, 1H, 5’‐OH), 5.11 (d, *J* = 4.8 Hz, 1H, 2’‐OH), 4.35 (q, *J* = 6.0 Hz, 1H, H‐3’), 4.10 (d, *J =* 8.1 Hz, 2H, BCN), 4.09 – 4.05 (m, 1H, H‐4’), 4.03 (d, *J =* 5.5 Hz, 2H, C*H_2_*), 3.89 (q, *J =* 3.4 Hz, 1H, H‐2’), 3.62 (dt, *J* = 11.7, 4.5 Hz, 1H, H‐5_a_’), 3.54 (dt, *J* = 5.7, 2.8 Hz, 1H, H‐5_b_’)., 2.27 – 2.08 (m, 6H, BCN), 1.55 (q, *J* = 10.4, 9.5 Hz, 2H, BCN), 1.35 – 1.26 (m, 1H, BCN), 0.91 – 0.83 (m, 2H, BCN). ^13^C‐NMR (126 MHz, DMSO‐*d_6_*): δ (ppm) = 157.6, 152.7, 149.6, 126.3, 102.4, 99.0, 94.5, 89.3, 87.2, 85.2, 75.2, 74.0, 70.5, 61.5, 30.9, 28.6, 20.8, 19.6, 17.6. HR‐MS (ESI): *m*/*z* (calculated for C_25_H_30_N_5_O_6_^+^): 496.2191 [M+H]^+^; found: 496.2186 [M+H]^+^.

### 4.13 Synthesis of **BCN‐DNA**

The synthesis of the oligonucleotide precursor **DNA‐NH_2_** of **DNA‐BCN** was performed using the K&A H‐6 DNA/RNA synthesizer and was done in the 3’ to 5’ direction. *Controlled Pore Glass* (CPG) with an occupancy of 1 µmol (500 Å) was employed. The CPG columns as well as the reagents required for the synthesis were bought commercially from GLENRESEARCH, CHEMGENES, PROLIGO and SIGMA ALDRICH. The synthesis of **DNA‐NH_2_** was carried out under argon as an inert gas. The synthetic phosphoramitide **34** [28, 40] was used as 0.1 M solution and the conventional phosphoramidites as 0.067 M solutions. A standard coupling protocol was used for the insertion of the conventional building blocks. For the synthetic building block **34**, the standard synthesis protocol was modified to achieve longer coupling times, which should ensure more effective insertion of the artificial building blocks. After the synthesis was completed, the CPG columns were dried under high vacuum for 1 h. The CPG column was opened and the column material transferred to a microcentrifuge tube. 700 µl aq. NH_4_OH (> 25%, *trace select*, FLUKA) was added to cleave **DNA‐NH_2_** from the solid phase and also to remove protective groups. The suspension was vortexed and was heated for 15 h at 55°C in a heating block. After cooling the suspension, the ammonia was removed using a *CHRIST Alpha RVC* vacuum concentrator (35 min, 35°C, 100 mbar). The supernatant solution was pipetted off and the residue was washed three times with 200 µl HPLC water The united centrifugates were lyophilized (25°C, 0.1 mbar) after freezing in liquid nitrogen on a *CHRIST Alpha 1–2 LD Plus* lyophilization unit. **DNA‐NH_2_** was purified using DMT affinity columns from GLEN RESEARCH (Glen‐pak DNA Purification Cartridges). The lyophilized crude **DNA‐NH_2_** was dissolved in 1 mL dd‐H_2_O. 1 mL NaCl‐solution (100 mg/mL) was added to the solution to gain an end salt concentration of 50 mg/mL. The affinity column was first pretreated with 0.5 mL MeCN and 1 mL 2 M Triethylammoniumacetat buffer (pH7). The oligonucleotide‐salt solution was applied on to the column in 1 mL aliquots. After that, 1 mL of TFA solution (2% in water) was added twice and the column was washed twice with 1 mL double distilled water. **DNA‐NH_2_** was eluted off the column with 1 mL 0.5% NH_4_OH in MeCN/water (1:1) and then evaporated to dryness under vacuum at 0.1 mbar and 25°C overnight. Approximately 1 µmol of the oligonucleotide was dissolved in 300 µL dry DMSO. 2.00 mg BCN – NHS ester was dissolved in 300 µL dry DMSO and added to the oligonucleotide. The solution was then mixed with 5.00 µL Et_3_N and shaken on a laboratory shaker at room temperature for 16 h. The solvent was then removed overnight under vacuum at 0.1 mbar and 25°C. A semi‐preparative RP‐HPLC was used to purify the postsynthetically modified **DNA‐BCN**. The separation was carried out on a Dionex Ultimate3000 with autosampler, pump module, column oven, multi‐diode array, RS fluorescence detector and fraction collector from THERMOFISHER SCIENTIFIC using Chromeleon 7 software. For semi‐preparative separation, VDSphere OptiBio PUR 300 C18‐SE column (250 × 10 mm, 5 µm) from VDS OPTILAB was used with a flow rate of 3.5 mL/min at a temperature of 40°C. A gradient of 0 – 30% MeCN and 0.1 M NH_4_OAc over 30 min served as the mobile phase (0 min: 0% MeCN; 30 min: 20% MeCN; 40 min: 20% MeCN). **DNA‐BCN** was dissolved in 300 µL dd water and was injected via the autosampler with an injection volume of 300 µL. The oligonucleotide was detected at a wavelength of 260 nm and 280 nm and collected in fractions. The purity of the collected fractions was verified by MALDI‐TOF mass spectrometry. The identified pure fractions were combined and lyophilized. The concentration of **DNA‐BCN** was calculated by using the *Lambert‐Beer law*. For this purpose, the optical density at *λ* = 260 nm was measured on an *ND‐1000 spectrophotometer* from NANODROP (in nucleic acid mode). The molar extinction coefficient ε_260_ of an oligonucleotide based only on natural bases at 260 nm in water is given by the following equation: ε_260_ = (n_A_ · ε_A_ + n_G_ · ε_G_ + n_C_ · ε_C_ + n_T_ · ε_T_) · 0.9 + ε**_23_** with n_A_, n_G_, n_C_, n_T_: Number n of conventional bases and ε_A,G,C,T_: molar extinction coefficient 260 nm, ε_A_ = 15,400 M^−1^cm^−1^; ε_G_ = 11,700 M^−1^cm^−1^; ε_C_ = 7,300 M^−1^cm^−1^; ε_T_ = 8,800 M^−1^cm^−1^´; ε**_23_** = 4,300 M^−1^cm^−1^ [28], 0.9: correction factor for hypochromicity. MS (MALDI‐TOF): calcd: *m*/*z* = 5386 [M]^+^, exp: *m*/*z* = 5389 [M]^+^.

### 4.14 SPSAC of **1–5** with **18** and **23**

Quartz cuvettes with a light path of 10 mm and a probe volume of 0.50 mL were used. All reactants were prepared as stock solutions with 4 mM concentration for dyes **1**–**5** and 20 mM for **18** and **23**. In each case, 2.5 µL were diluted with double‐distilled water to a total volume of 0.5 mL, resulting in a solvent composition of H_2_O/DMSO (99:1 v/v%), concentration of 20 µM for dyes **1**–**5** of 100 µM for **18** and **23**, a ratio between dye:**18** and dye:**23** of 1:10 (Table S1). To monitor the progression of the reaction, the absorption was measured time‐dependently, representatively for **1** and **2** with **23** (Figures S37,S38). The fluorescence spectra were measured on a Fluoromax‐4 spectrometer of the company HORIBA‐SCIENTIFIC with a Peltier‐element for temperature control. The measurements were corrected for Raman scattering of the respective solvent. Fluorescence spectra were recorded time‐dependently to monitor the increase in fluorescence intensity (Figures S39–S49). The excitation wavelengths were adjusted to the dyes **1**–**5** (Table S2). A blank sample with H_2_O/DMSO 99:1 was used to correct the starting fluorescence in addition to the dye (I_0_). To determine the reaction rate constant k_2_ by the increase in fluorescence, fluorescence spectra obtained were corrected, normalized and then integrated over the wavelengths λ_exc_ + 15 nm to 800 nm. The time‐dependent fluorescence intensities were fitted using a monoexponential function. The rate constant *k_2_* can be calculated from the curvature *k*_obs_ and is given in M^−1^s^−1^: $y=a+be^{-k_{obs}x}$.

### 4.15 SPSAC of **1–5** with **DNA‐BCN**

Quartz cuvettes with a light path of 10 mm and a probe volume of 0.50 mL were used. The stock solution of **DNA‐BCN** was prepared in water and the stock solutions of dyes **1**–**5** were diluted 1:10 with DMSO to obtain a concentration of 400 µM. This resulted in an end concentration of 2.0 µM dye **1**–**5** and 10 µM **DNA‐BCN** in water/DMSO 99:1 (Table S4). The fluorescence spectra were measured on a Fluoromax‐4 spectrometer of the company HORIBA‐SCIENTIFIC with a Peltier‐element for temperature control. The measurements were corrected for Raman scattering of the respective solvent. Fluorescence spectra were recorded time‐dependently to monitor the increase in fluorescence intensity (Figure 3) and were fitted using a monoexponential function as described above. **DNA‐3**, **DNA‐4** and **DNA‐5** were identified by ESI‐MS analysis (Table S5 and Figures S60, S61).

### 4.16 Cell Experiments

All cell experiments were carried out under sterile conditions. The materials and equipment used, as well as protective gloves, were disinfected with an 80% ethanol solution before use. Consumables that were not sterile‐packaged were first autoclaved before use. For transfection experiments, human cervical carcinoma cells (HeLa) from the company ATCC, were cultured under sterile conditions in Dulbecco's modified Eagle medium ([+] 4.5 g/L D‐glucose, L‐glutamine, [+] pyruvate, DMEM, Gibco), supplemented with 10% fetal calf serum (HI FBS, Gibco) and 1 U/mL penicillin‐streptomycin (Gibco). The cells were incubated in a BINDER incubator at 37°C, 5% CO_2_ and humidity of 90%. The cells were passaged at a confluence of approximately 80%. First the medium was removed and the cells washed with 5 mL Dulbecco's Phosphate Buffered Saline (DPBS, Gibco). To detach the cells from the bottom of the cell culture flask, 1 mL of a 0.25% trypsin/EDTA solution were put on the cells and the cells were incubated for 3 min at 37°C. Then 9 mL DMEM was put on the cells to stop the trypsination and the cell suspension was transferred to a new cell culture flask in the appropriate dilution with fresh medium. For subsequent cell experiments, the cell number was determined using a Neubauer counting chamber. In a next step, the cells were diluted to the desired cell number and transferred to μ‐slides 8‐well (IBIDI). For the transfection experiments 2∙10^4^ cells per well were seeded in a volume of 200 µL in an IBIDI (μ‐slide 8 well ibiTreat, IBIDI) plate. Simultaneously, the oligonucleotide was transfected using Lipofectamine (Lipofectamine 2000, Invitrogen). For this purpose, 0.15 µL Lipofectamine stock solution was mixed with 4.85 µL Opti‐MEM (serum‐reduced medium, Gibco) per well with 150 nM of **DNA‐BCN** or the in vitro clicked **DNA‐1**, **DNA‐2**, **DNA‐3** (positive control) and incubated for 20 min at room temperature. The formed lipoplex was pipetted into the cell suspension in the IBIDI well and 2 µL Endoporter (GENE TOOL) per well was added, and the IBIDI was put on the laboratory shaker for 1 min. After the cells were incubated for 24 h the cell culture medium was removed and the cells were washed with DPBS. 4.00% paraformaldehyde solution was put on the cells and for 1 min incubated on the laboratory shaker. The fixation was stopped by adding 50.0 mM glycine and 50.0 mM NH_4_Cl in DPBS for 5 min. After washing the cells two times with DPBS 150 nM (**3**), 300 nM (**4**), and 600 nM (**5**) (stock solution in DMSO, diluted in DMEM) was added. 2 h later the cells were washed twice with DPBS (2 × 15 min). Draq5 was added to stain the cell core and the cells were examined using a confocal fluorescence microscope (Leica DMI8, TCS SP8 inverted microscope) with a HC PL APO 63x/1.40 OIL CS2 objective. The fluorophore conjugates were excited with a 488 nm laser (10% intensity) (**3**/**DNA‐3** and **4**/**DNA‐4**) and 405 nm (10% intensity) (**5**/**DNA‐5**) and the emission was measured in the range of 520–650 nm.

## Conflicts of Interest

The authors declare no conflicts of interest.

## Supporting information



The authors have cited additional references within the Supporting Information [42].

## Data Availability

The data that support the findings of this study are available on request from the corresponding author. The data are not publicly available due to privacy or ethical restrictions.
