# Supplementary material for: Fluorogenic Sydnones for Bioorthogonal Labeling of DNA
Source: Chemistry. 2026 Jun 23;32(37):e71314. doi: 10.1002/chem.71314 (PMC13628573; doi:10.1002/chem.71314)
Supplement: Supplementary file 1 [file CHEM-32-e71314-s001.pdf]

Fluorogenic sydnones for bioorthogonal labeling of DNA

Kerstin Müller,[a] and Hans-Achim Wagenknecht*[a]

[a] Dr. Kerstin Müller, Prof. Dr. Hans-Achim Wagenknecht
Institut of Organic Chemistry
Karlsruhe Institute of Technology (KIT)
Fritz-Haber-Weg 6, 76131 Karlsruhe, Germany
* Corresponding author, E-mail: Wagenknecht@kit.edu

Supporting Information

Table of content page

1. Materials and methods 2

2. Organic-chemical synthesis 4

3. DNA synthesis 30

4. SPSAC in vitro 32

5. Cell experiments 44

1. Materials and methods

Synthesis

The reagents used for synthesis were purchased from ABCR, ALFA AESAR, CARBOLUTIONS, FLUKA, MERCK, SIGMA ALDRICH, BLDpharm, TCI and VWR and used without further purification. Solvents were purchased from SIGMA ALDRICH, ACROS ORGANICS and FISCHER, which were at least quality level *pro analysi* (*p.A.*). Dry solvents were also purchased, stored and used under argon. Technical solvents were used for work-up and for chromatographic purification, unless otherwise specified. Double deionised water (*Milli-Q® Direct 8/16* system from Merck *Millipore*) and high purity solvents (HPLC *grade*) were used for HPLC separations. Buffers used were filtered before. Deuterated solvents were bought from EURISO-TOP.

Air- and water-sensitive reactions were carried out using Schlenk technique under an argon atmosphere (Argon 5.0, Air Liquide, 99.999 % purity). The glass equipment was previously heated several times in a high vacuum and flushed with argon several times. Liquids were taken utilizing plastic syringes and cannulas or pipettes of the Eppendorf brand. If required, the solvents were degassed by passing in argon or by applying the *freeze-pump-thaw* method. Solids were transferred using weighing paper. For reactions at low temperatures, the following cooling mixtures were used: 0 °C: ice in water, -21 °C: NaCl in ice, -78 °C: dry ice in isopropanol. Solvents were removed under reduced pressure on a rotary evaporator at a water bath temperature of 45 °C.

Chromatography

For thin-layer chromatography, aluminum plates coated with silica gel (silica gel 60, F_{254}, layer thickness 0.250 mm) from MERCK were used. For the detection of the spot samples, UV-active samples were detected with UV light, by fluorescence quenching at λ = 254 nm or fluorescence excitation at λ = 366 nm. UV-inactive samples were stained with potassium permanganate solution (1.50 g $KMnO_4$, 10.0 g K_2CO_3, 1.25 mL 10 % NaOH solution, 200 mL H_2O). Saccharides were stained with a solution of sulfuric acid in methanol (5 % H_2SO_4) and amines with a ninhydrin solution (3.00 g ninhydrin in 200 mL EtOH).

Column chromatography was used to purify the crude products. Silica gel 60 (pore size 60 Å, particle size 40-63 µm) from SIGMA ALDRICH served as stationary phase. The raw products were adsorbed on *Celite S*, dry loaded or applied as a solution. Elution was carried out with overpressure generated by a hand pump.

Reversed-phase high-performance liquid chromatography (HPLC) of oligonucleotides was carried out on a THERMO SCIENTIFIC HPLC instrument (*Dionex UltiMate3000* autosampler, pump module, column oven, multidiode array, RS fluorescence detector, fraction collector, *Chromeleon 7* software). The separation was performed on a semi-preparative column (*VDSpehr OptiBio PUR 300* S18-SE column (250 x 10 mm, 5 µm)). The analytes were applied to the column with an injection volume of 300 µL and a flow rate of 3.50 mL/min, unless otherwise stated. The column was equilibrated with 0.05 M ammonium acetate buffer in water and eluted with various gradients of acetonitrile. The signals were measured using a UV diode array detector at wavelengths of 260 nm, 290 nm and the characteristic absorption of the respective artificial building blocks. For the monitoring of the SPSAC reactions via HPLC-MS an analytical column (*VDSpehr OptiBio PUR 300* S18-SE column (250 x 4.6 mm, 5 µm)) was used. A solvent gradient of acetonitrile and double-distilled water was used. The analytes were applied on the column with an injection volume of 20 µL and a flow rate of 1.00 ml/min, unless otherwise

stated. For the analytical runs with the sydnone fluorophores 0.1 % TFA was additionally added to the solvents. The signals were measured using a UV diode array detector at wavelengths of 260 nm, 290 nm and the characteristic absorption of the respective artificial building blocks.

NMR Spectroscopy

Nuclear magnetic resonance analyses were performed in deuterated solvents on a BRUKER *Advance 400* (400 MHz ^{1}H -NMR or 101 MHz ^{13}C-NMR) or BRUKER *Advance 500* (500 MHz ^{1}H-NMR or 126 MHz ^{13}C-NMR). For the measurement, 5-10 mg of the sample were dissolved in approx. 0.5 ml of deuterated solvent in a measuring tube with a diameter of 5 mm. The deuterated solvents employed were [d6]-DMSO, [d1]-$CDCl_3$ and [d4]-MeOD. The measured chemical shifts (δ) were given in *parts per million* (ppm) and refer to tertramethylsilane as 0.0 ppm. The spectrum was calibrated to the signals of the not fully deuterated solvent used; [*d6*]-DMSO: ^{1}H-NMR: δ = 2.50 ppm and ^{13}C-NMR: δ = 39.52 ppm, [*d1*]-$CDCl_3$: ^{1}H-NMR: δ = 7.26 ppm and ^{13}C-NMR: δ = 77.16 ppm, [*d4*]- MeOD: ^{1}H-NMR: δ = 3.31 ppm and ^{13}C-NMR: δ = 49.00 ppm. The measured coupling constants *J* were given in Hertz (Hz). The multiplicities of the signals were labelled with the abbreviations s (singlet), d (doublet), dd (doublet of doublet), t (triplet), q (quartet), quin (quintet) and m (multiplet) and br (broadened signal). The spectra were evaluated with the help of the software MESTRENOVA. The chemical shifts of known solvents were taken from known literature sources for example *Fulmer et al*.[35]

Mass Spectrometry

MALDI-TOF measurements were carried out on an *AXIMA Confidence* spectrometer from *SHIMADZU*. A standard plate made of stainless steel from the company Bruker with 386 application points was used as the target. A matrix of 3- hydroxypicolinic acid (saturated solution in acetonitrile/water=1:1) and diammonium hydrogen citrate (0.44 M in water) in a 9:1 mixture was used for DNA mass measurements. The signals were reported by their mass to charge ratio (m/z).

Small molecule masses were recorded on a Q Exactive Orbitrap and an LTQ Orbitrap XL from THERMO FISHER SCIENTIFIC using electron spray ionization (ESI). The signals were given in mass/charge ration (m/z) and the molecular ion was shown as $[M]^+$ or in protonated form as $[M+H]^+$ or $[M-H]^+$ in positive mode.

Optical spectroscopy

The examination of the synthesized substances was carried out in quartz glass cuvettes from STARNA path length 1 cm, sample volume 1 mL or 0.5 mL) at a temperature of 20 °C. All spectra were baseline-corrected using a blank.

The UV/Vis absorption spectra were recorded on a Cary3500 spectrometer from AGILANT with Peltier element from AGILENT. The samples were prepared using 1 cm quartz glass cuvettes (STARNA) with a volume of 1 mL at 25 °C. To determine the extinction coefficients, different concentrations were measured and applied to Lambert-Beer’s law.

Fluorescence spectra were recorded on a *Fluoromax-4* spectrofluorometer from HORIBA-SCIENTIFIC with a Peltier element from Horiba Scientific at 20 °C. The spectrometer was calibrated using the Raman scatter of water as a reference to ensure accurate wavelength measurement.

2. Organic-chemical synthesis

Compound 1

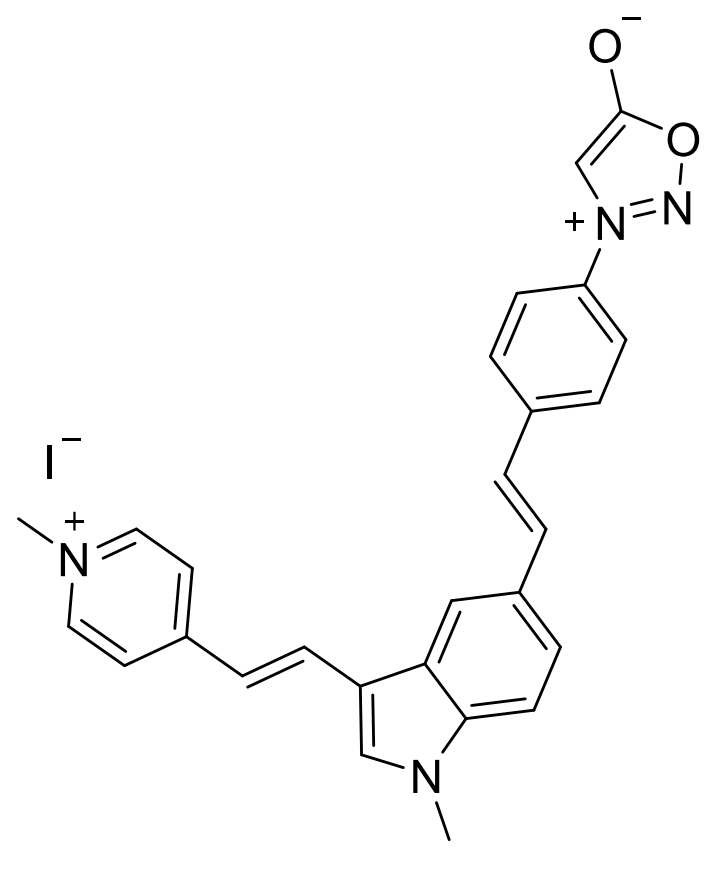

$C_{27}H_{23}N_4O_2^+I^-$
562.41 g/mol

In a heated 10 mL crimp vial under argon, 0.100 g **7**[31] (0.220 mmol, 1.00 eq.), 0.010 g $Pd_2(dba)_3$ (0.010 mmol, 0.05 eq.), 0.031 g Qphos (0.040 mmol, 0.20 eq.) and 0.083 g **6**[25] (0.440 mmol, 2.00 eq.) dried under high vacuum. The solids were then dissolved in 2.00 mL dry DMF, and 0.15 mL triethylamine (0.111 g, 1.10 mmol, 5.00 eq.) was added. The reaction was stirred for 18 h in a metal heating block at 80 °C. After the solution had cooled to room temperature Et_2O was added until no further precipitate was formed. The suspension was transferred into reaction tubes. The solution was centrifuged and the supernatant was pipetted off and discarded. Subsequently, Et_2O was added and the suspension was vortexed and centrifuged again in the next step and the supernatant discarded. This was carried out two more times. The residue was dried in high vacuum. The product was obtained as a brown solid in a yield of 0.062 g (0.110 mmol, 49%).

^{1}H-NMR (400 MHz, DMSO-d_6): δ (ppm) = 8.72 (d, J = 6.4 Hz, 2H, arom.), 8.42 (s, 1H, arom.), 8.24 (d, J = 16.2 Hz, 1H, R-CH=CH-R), 8.17 (d, J = 6.5 Hz, 2H, arom.), 8.02 – 7.96 (m, 3H, arom., R-CH=CH-R), 7.93 (d, J = 8.6 Hz, 2H, arom.), 7.82 (s, 1H, arom.), 7.73 – 7.61 (m, 3H, arom.), 7.48 (d, J = 16.5 Hz, 1H, R-CH=CH-R), 7.35 (d, J = 16.2 Hz, 1H, R-CH=CH-R), 4.19 (s, 3H, R-CH_3), 3.91 (s, 3H, R-CH_3).

^{13}C-NMR (126 MHz, DMSO-d_6): δ (ppm) = 168.5, 154.0, 144.3, 141.7, 138.1, 136.1, 135.3, 132.8, 130.2, 127.4, 125.8, 124.8, 121.8, 119.8, 117.2, 113.0, 111.5, 94.5, 46.3, 33.3.

HR-MS (ESI): m/z (calculated for $C_{27}H_{23}N_4O_2^+$): 435.1816 $[M]^+$; found: 435.1811 $[M]^+$.

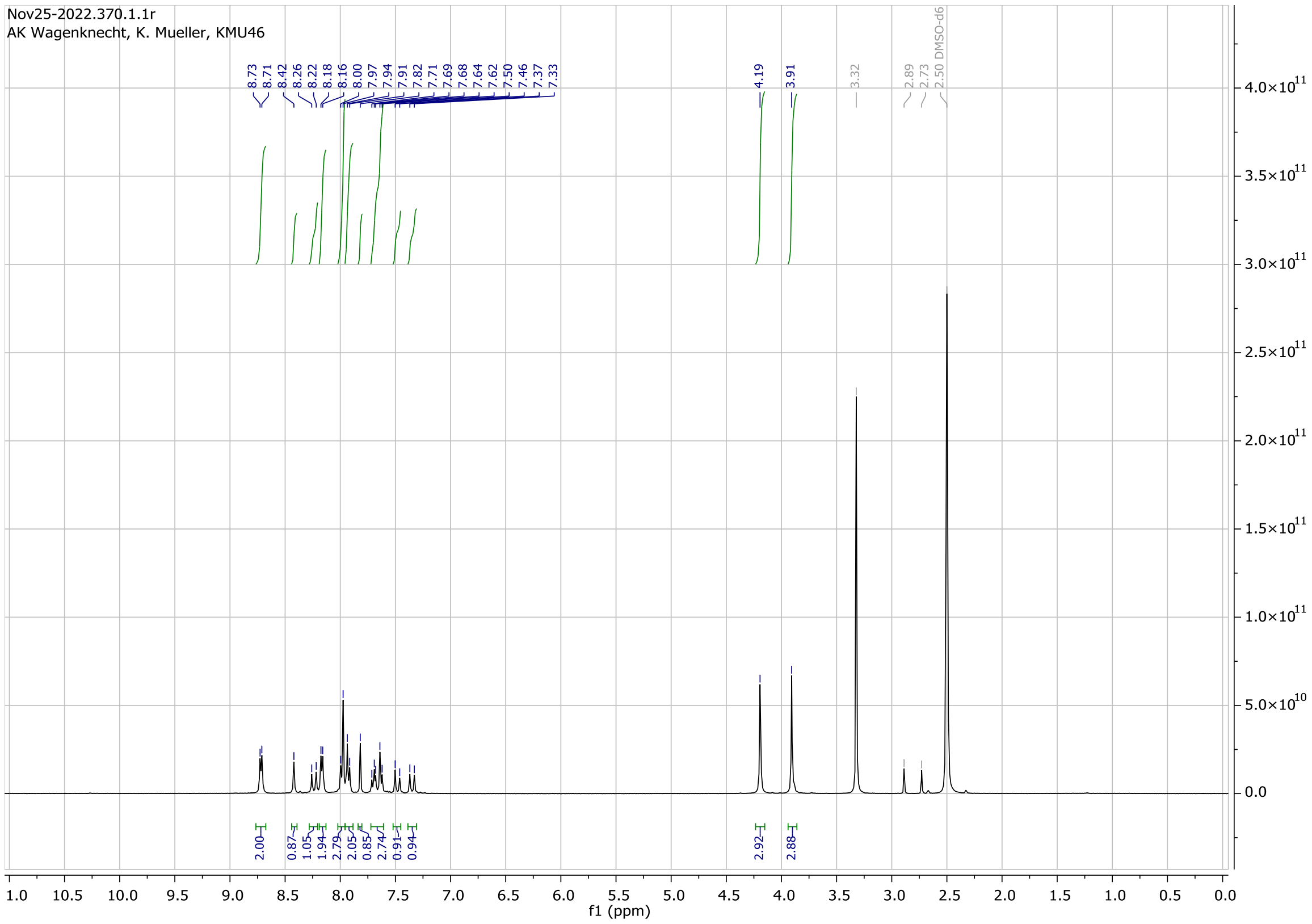

Figure S1. ^{1}H-NMR spectrum (400 MHz, DMSO-d_6) of **1**.

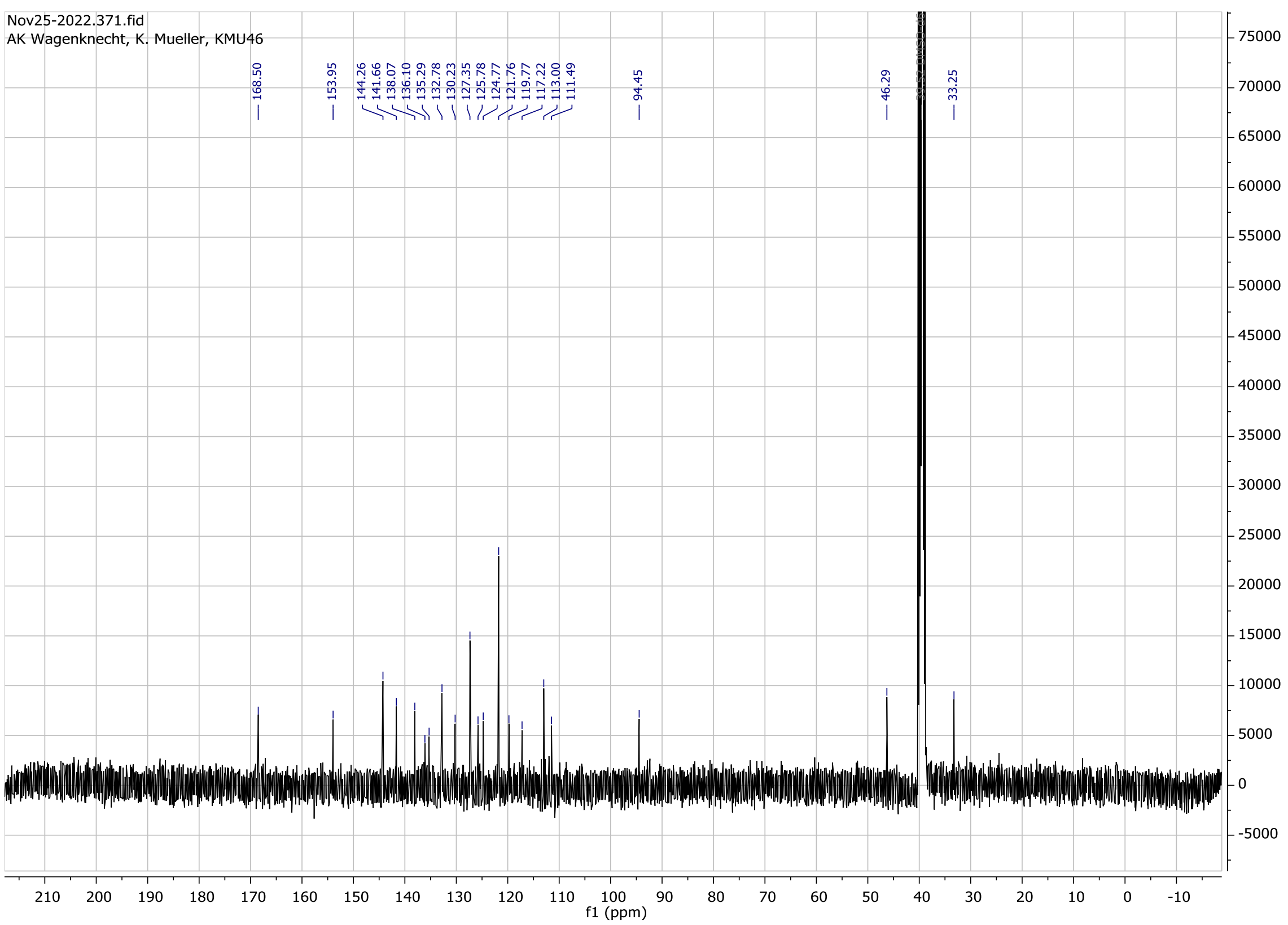

Figure S2. ^{13}C-NMR spectrum (126 MHz, DMSO-d_6) of **1**.

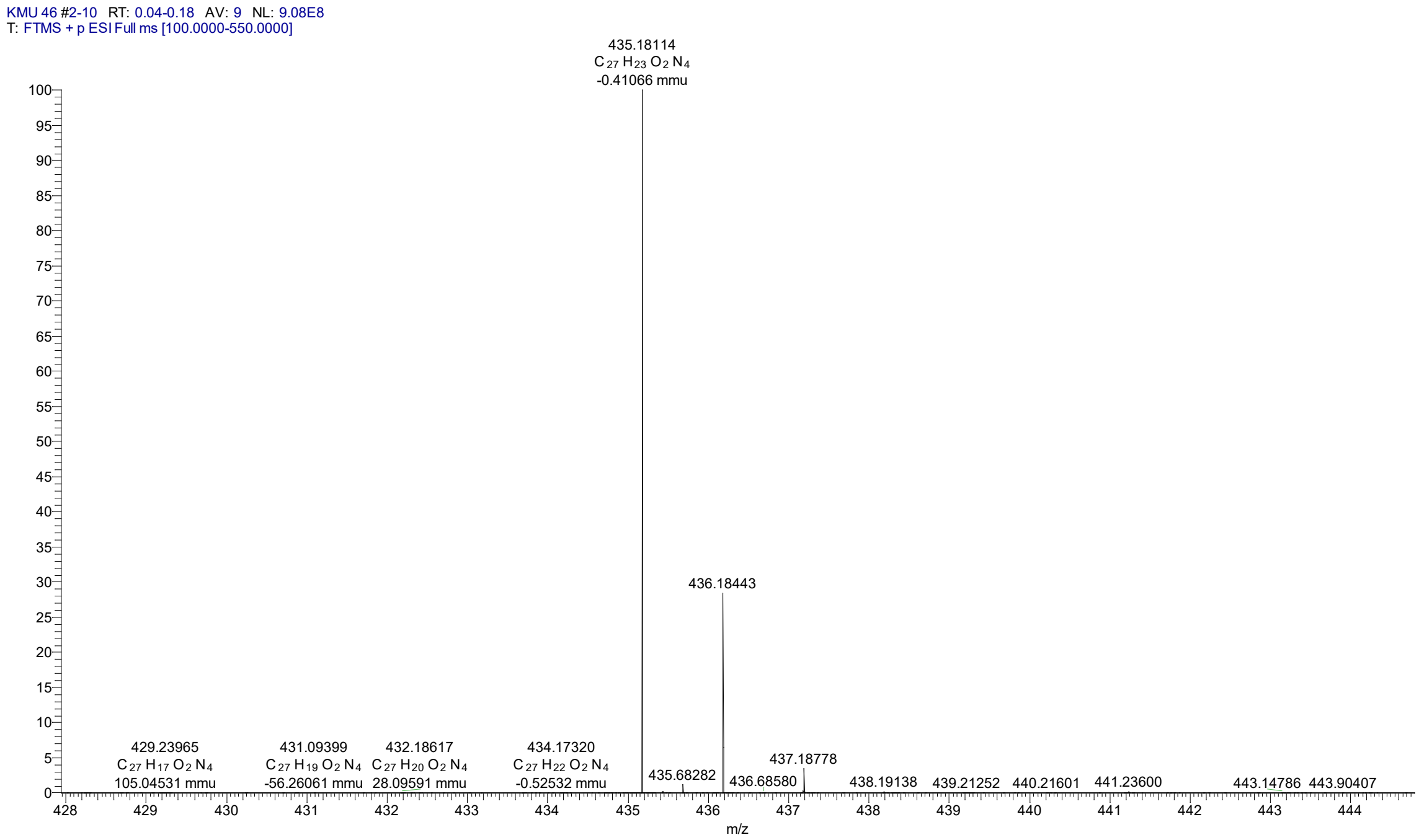

Figure S3. HR-MS analysis (ESI) of **3**.

Compound 2

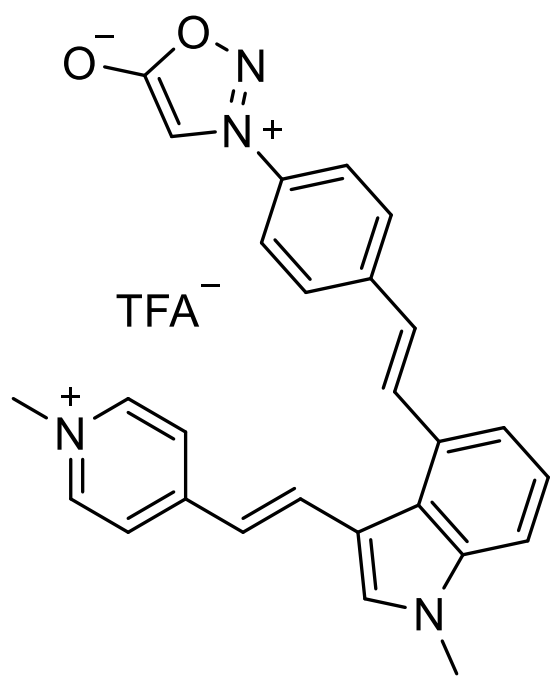

$C_{27}H_{23}N_4O_2^+C_2F_3O_2^-$
548.51 g/mol

In a heated 10 mL crimp vial under argon, 0.100 g **8**[30] (0.220 mmol, 1.00 eq.), 0.010 g $Pd_2(dba)_3$ (0.010 mmol, 0.05 eq.), 0.031 g Qphos (0.040 mmol, 0.20 eq.) and 0.083 g **6**[25] (0.440 mmol, 2.00 eq.) dried under high vacuum. The solids were then dissolved with 2.00 mL dry DMF and 0.15 mL Et_3N (0.111 g, 1.10 mmol, 5.00 eq.) was added. The reaction was stirred for 18 h in a metal heating block at 80 °C. After the solution had cooled to room temperature Et_2O was added until no further precipitate was formed. The suspension was transferred into reaction tubes. The solution was centrifuged and the supernatant was pipetted off and discarded. Subsequently, Et_2O was added and the suspension was vortexed and centrifuged again in the next step and the supernatant discarded. This was carried out two more times. The residue was dried in high vacuum and purified via reversed-phase column chromatography. The product was obtained as a brown solid in a yield of 0.056 g (0.099 mmol, 45%).

^{1}H-NMR (500 MHz, DMSO-d_6): δ (ppm) = 8.68 (d, J = 6.8 Hz, 2H, arom.), 8.41 (d, J = 15.8 Hz, 1H, R-CH=CH-R), 8.26 (d, J = 16.1 Hz, 1H, R-CH=CH-R), 8.21 (s, 1H, arom.), 8.04 (d, J = 8.7 Hz, 2H,

arom.), 7.98 (t, J = 7.9 Hz, 4H, arom.), 7.82 (s, 1H, arom.), 7.57 (d, J = 8.1 Hz, 1H, arom.), 7.51 (d, J = 7.4 Hz, 1H, arom.), 7.33 (t, J = 7.8 Hz, 1H, arom.), 7.28 (d, J = 16.1 Hz, 1H, R-CH=CH-R), 7.14 – 7.09 (m, 1H, R-CH=CH-R), 4.17 (s, 3H, R-CH_3), 3.91 (s, 3H, R-CH_3).

^{13}C-NMR (126 MHz, DMSO-d_6): δ (ppm) = 168.6, 153.3, 144.5, 141.3, 138.0, 136.0, 133.2, 132.6, 130.8, 130.4, 129.1, 128.0, 123.9, 122.8, 121.8, 119.8, 118.6, 113.2, 111.2, 94.6, 46.5, 33.4.

HR-MS (ESI): m/z (calculated for $C_{27}H_{23}N_4O_2^+$): 435.1816 [M]$^+$; found: 435.1811 [M]$^+$

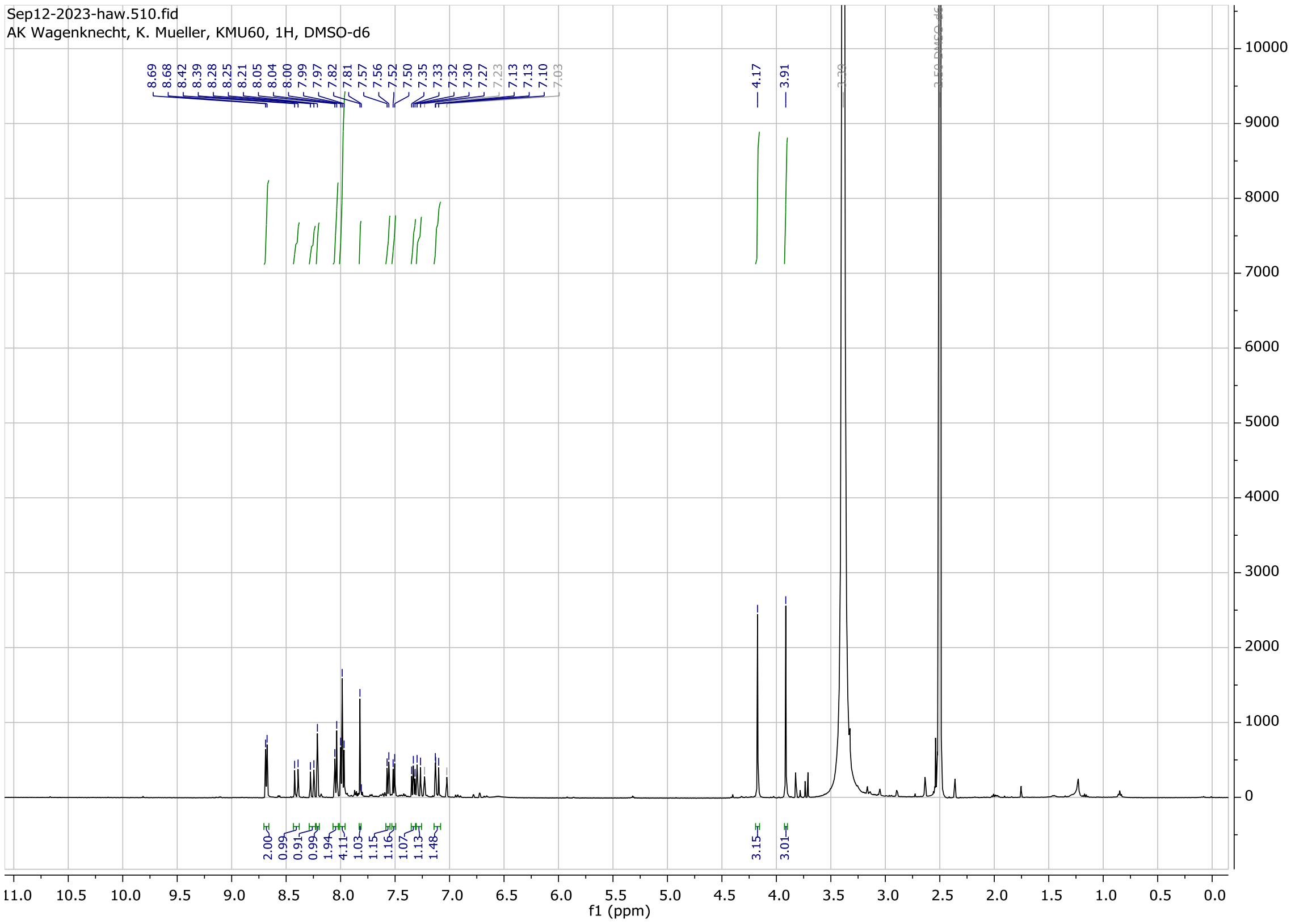

Figure S4. ^{1}H-NMR spectrum (500 MHz, DMSO-d_6) of **2**.

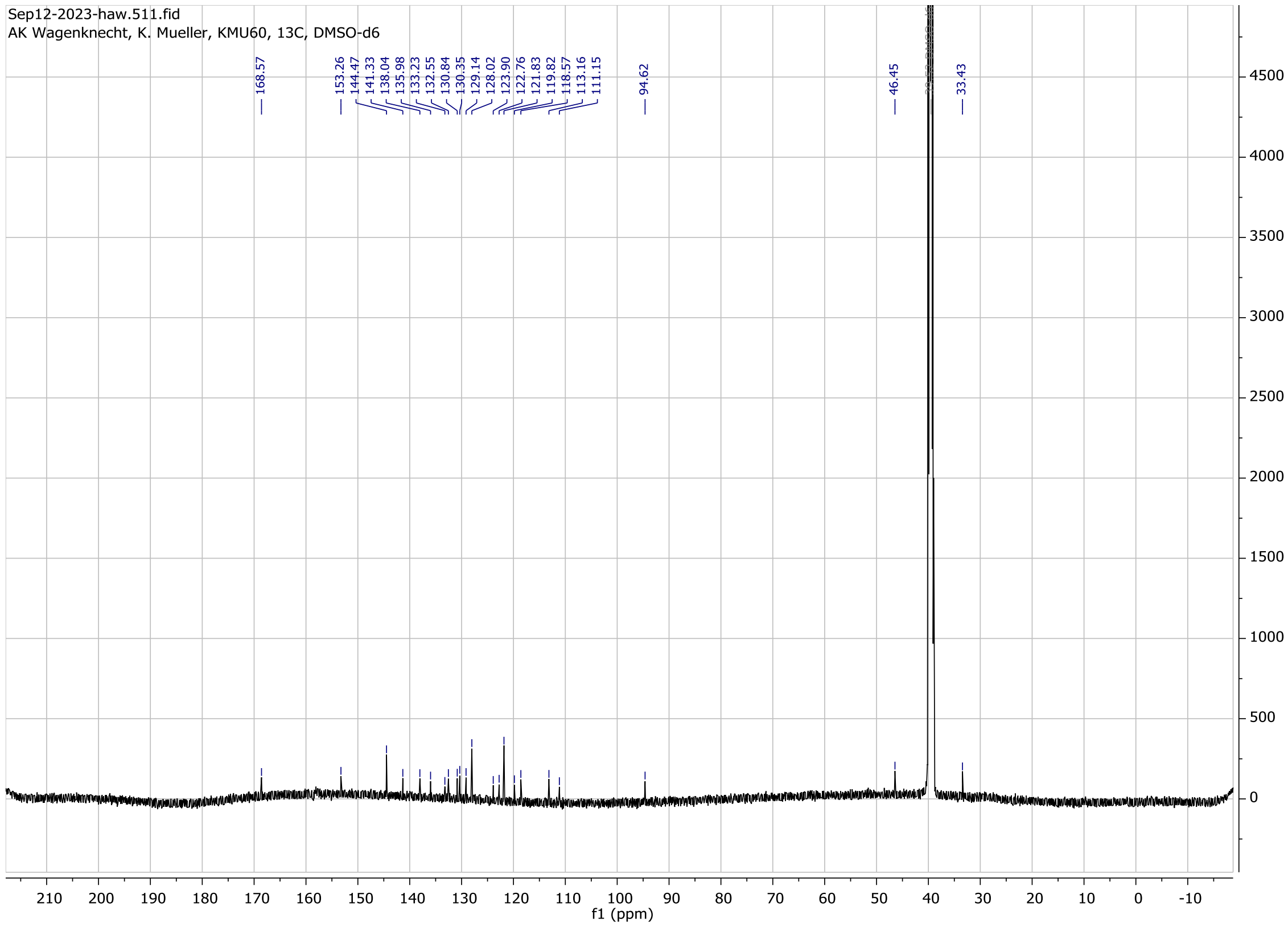

Figure S5. ^{13}C-NMR spectrum (126 MHz, DMSO-d_6) of **2**.

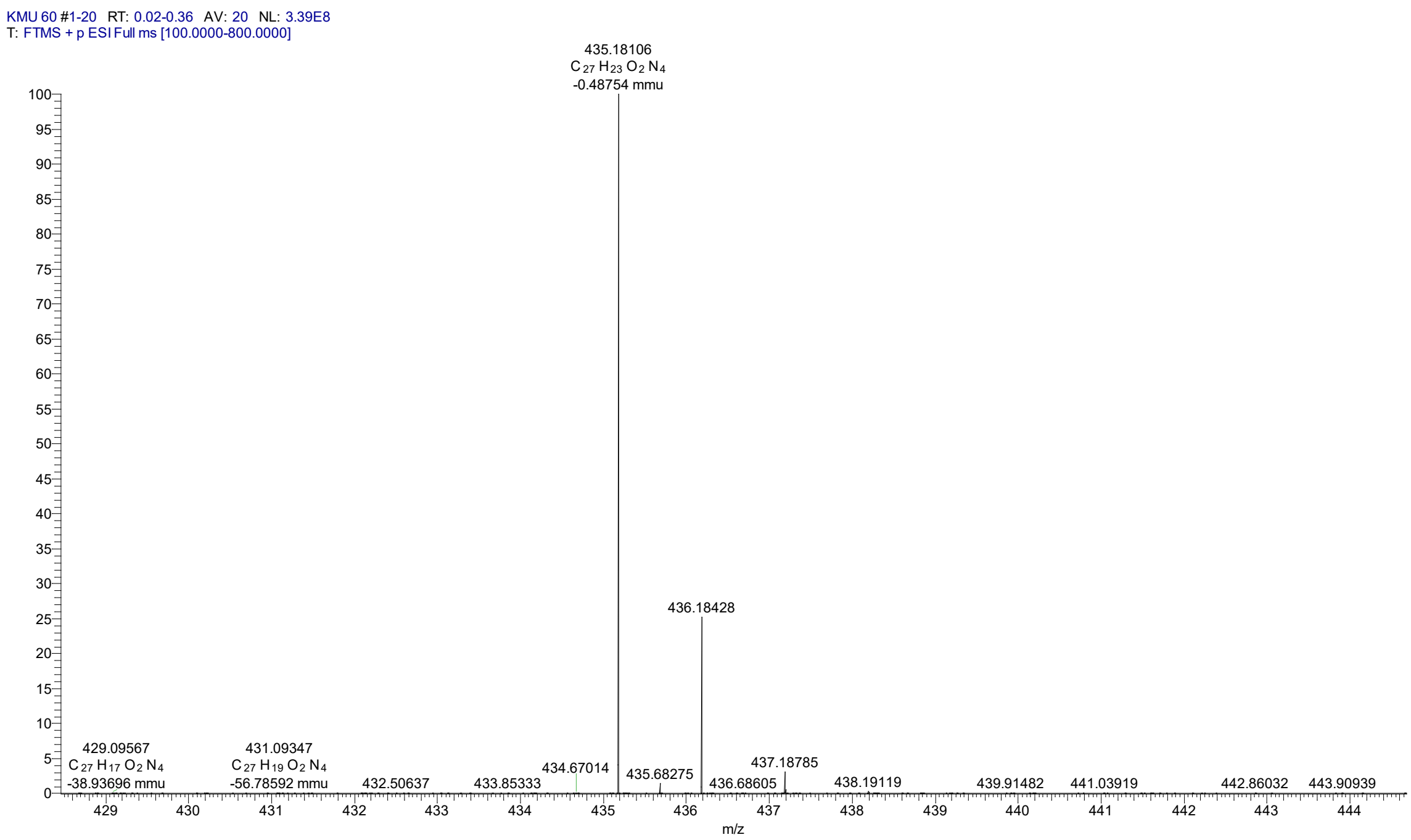

Figure S6. HR-MS analysis (ESI) of **2**.

Compound 11

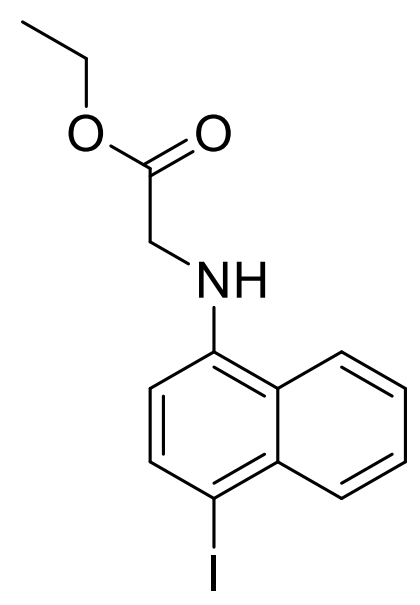

$C_{14}H_{14}INO_2$
355.18 g/mol

In a heated Schlenk flask under argon 6.50 g **10**[32] (24.2 mmol, 1.00 eq.) was dissolved in 45 mL DMF. 4.01 g K_2CO_3 (28.9 mmol, 1.20 eq.) was added. 3.21 mL ethyl bromoacetate (4.84 g, 28.9 mmol, 1.20 eq.) was then added in portions for 10 minutes. The reaction mixture was allowed to stir overnight at room temperature. 5.25 mL diethylamine (3.71 g, 50.7 mmol, 2.10 eq.) was added in portions for 5 min and the reaction solution was allowed to stir for 1 h. The solid was filtered off. The solvent of the filtrate was removed. The residue was purified by automated column chromatography (cyclohexane:EtOAc 0-40 %). The product was obtained as a red brown solid with a yield of 3.20 g (9.01 mmol, 37%).

^{1}H-NMR (400 MHz, DMSO-*d*$_6$): δ (ppm) = 8.15 (d, *J* = 8.3 Hz, 1H, arom.), 7.89 (dd, *J* = 8.0, 1.3 Hz, 1H, arom.), 7.82 (d, *J* = 8.2 Hz, 1H, arom.), 7.60 (t, *J* = 7.2, 1H, arom.), 7.52 (t, *J* = 7.1 Hz, 1H, arom.), 6.94 (t, 1H, R-NH-R), 6.20 (d, *J* = 8.2 Hz, 1H, arom.), 4.13 (q, *J* = 7.1 Hz, 2H, R-CH_2-CH_3), 4.08 (d, *J* = 6.2 Hz, 2H, R-NH-CH_2-R), 1.20 (t, *J* = 7.1 Hz, 3H, R-CH_2-CH_3).

^{13}C-NMR (126 MHz, DMSO-*d*$_6$): δ (ppm) = 170.8, 144.6, 137.6, 133.7, 131.6, 127.8, 125.2, 124.1, 122.2, 105.3, 82.4, 60.4, 44.8, 14.1.

HR-MS (ESI): m/z (calculated for $C_{14}H_{15}INO_2^+$): 356.0142 [M+H]$^+$; found: 356.0140 [M+H]$^+$.

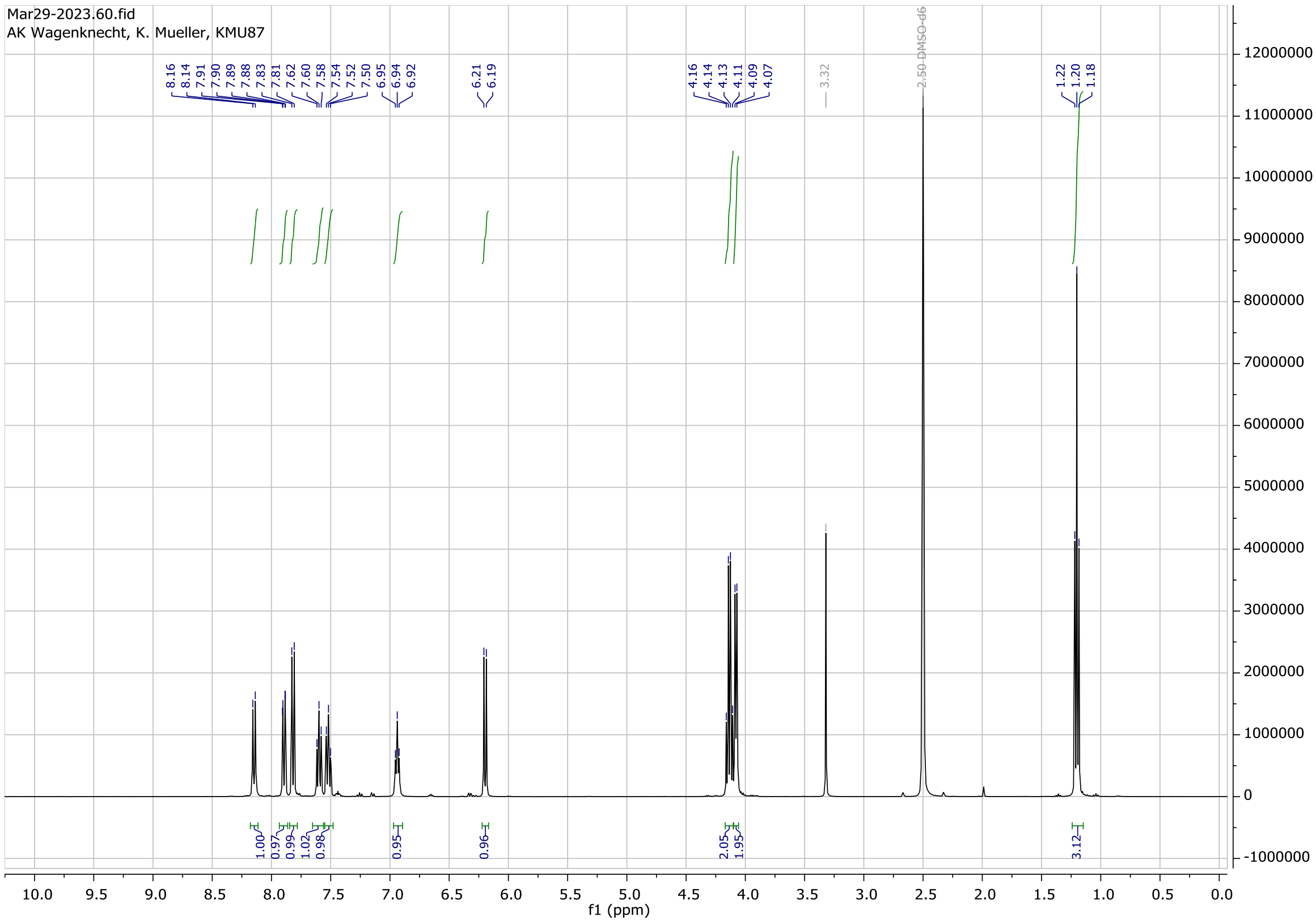

Figure S7. ^{1}H-NMR spectrum (400 MHz, DMSO-d_6) of **11**.

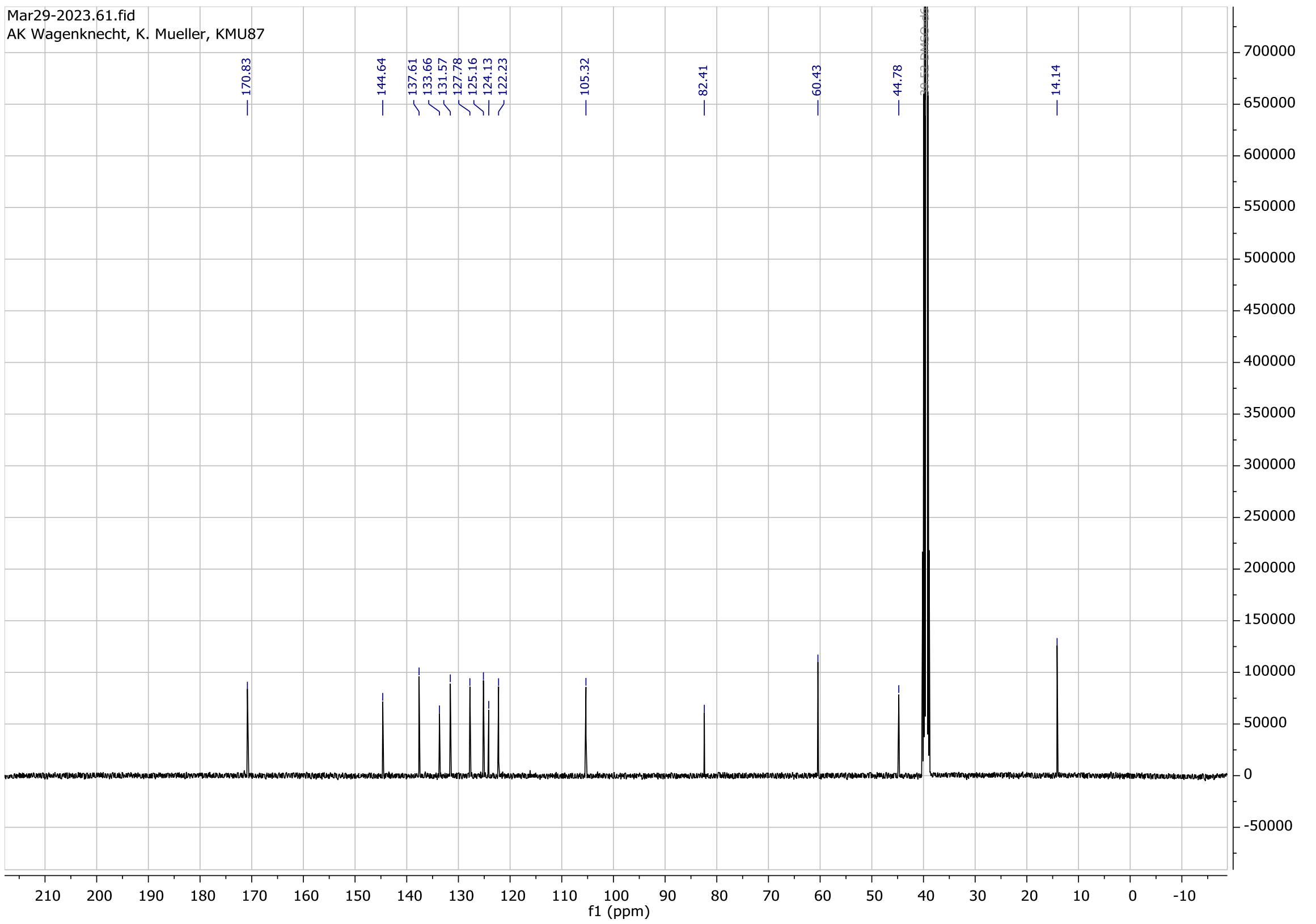

Figure S8. ^{13}C-NMR spectrum (126 MHz, DMSO-d_6) of **11**.

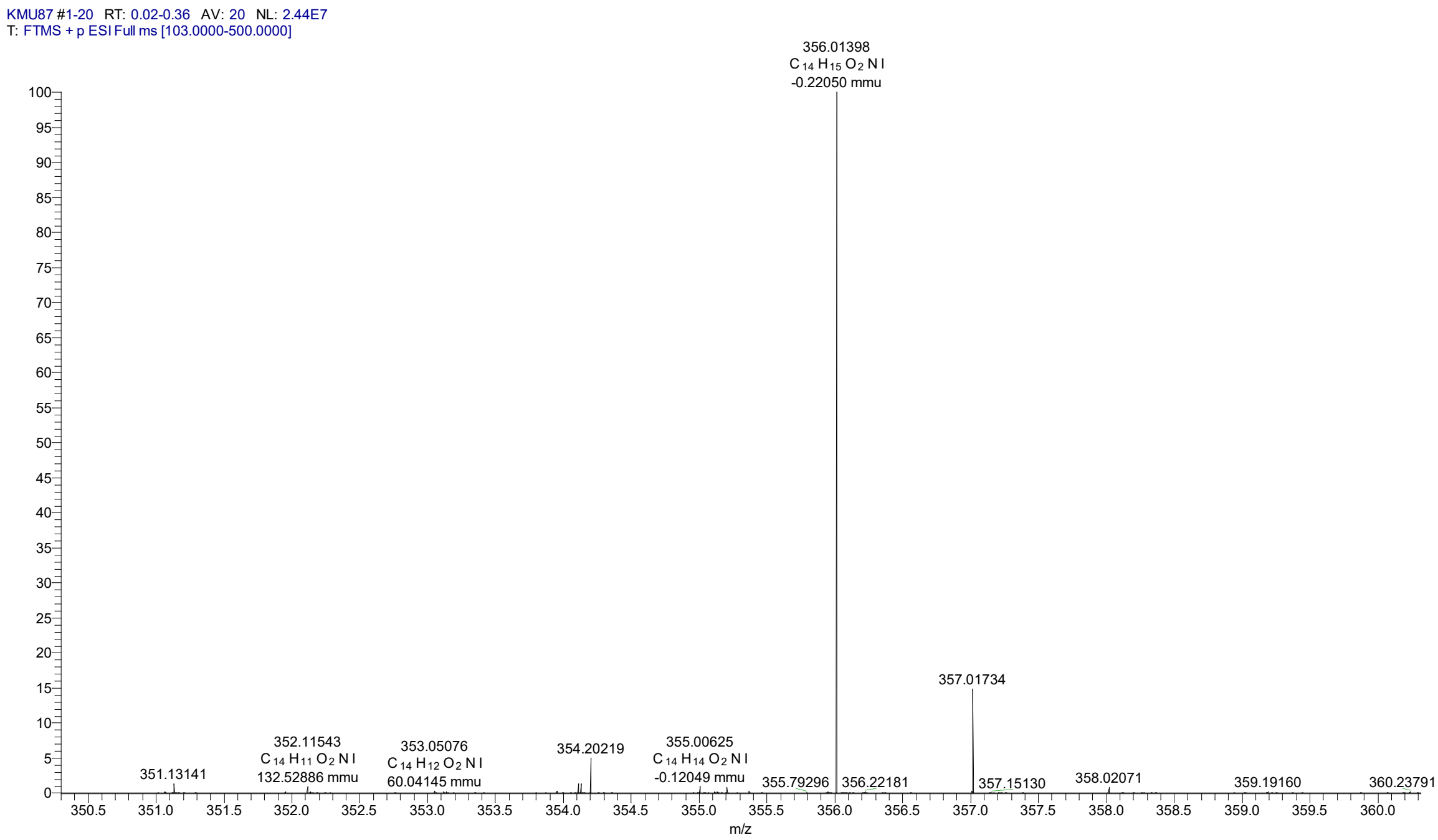

Figure S9. HR-MS analysis (ESI) of **11**.

Compound 12

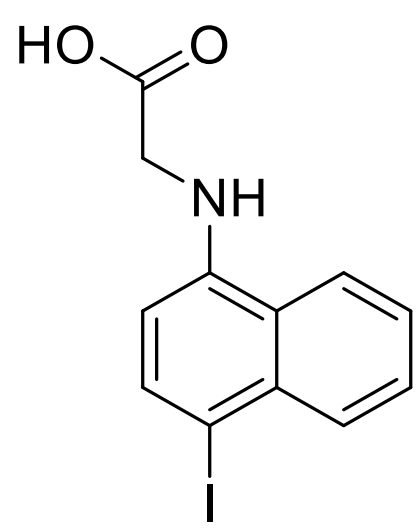

$C_{12}H_{10}INO_2$
327.12 g/mol

In a Schlenk flask under argon, 4.48 g **11** (12.6 mmol, 1.00 eq.) was placed and dissolved in 16 mL THF. The solution was cooled to 0 °C with an ice bath. Meanwhile, 0.604 g LiOH (25.2 mmol, 2.00 eq.) was dissolved in 16 mL H_2O and then added to the reaction solution. The solution was allowed to stir at 0 °C for 1 h and then at room temperature for 1 h. The THF was evaporated off. A pH of 2 was then adjusted with HCl (2 M) and the resulting precipitate was filtered off and washed with a small amount of water. The residue was dried under reduced pressure. The product was obtained as a light violet solid in a yield of 3.99 g (12.2 mmol, 97%).

^{1}H-NMR (400 MHz, DMSO-d_6): δ (ppm) = 12.66 (s, 1H, R-COOH), 8.13 (d, *J* = 8.3 Hz, 1H, arom.), 7.89 (d, *J* = 8.3 Hz, 1H, arom.), 7.82 (d, *J* = 8.2 Hz, 1H, arom.), 7.59 (t, *J* = 7.3, 1H, arom.), 7.51 (t, *J* = 7.6 Hz, 1H, arom.), 6.83 (s, 1H, R-NH-R), 6.21 (d, *J* = 8.2 Hz, 1H, arom.), 3.98 (s, 2H, R-NH-CH_2-R).

^{13}C-NMR (126 MHz, DMSO-d_6): δ (ppm) = 172.2, 144.8, 137.7, 133.7, 131.5, 127.7, 125.1, 124.1, 122.2, 105.3, 82.1, 44.7.

HR-MS (ESI): m/z (calculated for $C_{12}H_{11}INO_2^+$): 327.9829 [M+H]$^+$; found: 327.9827 [M+H]$^+$.

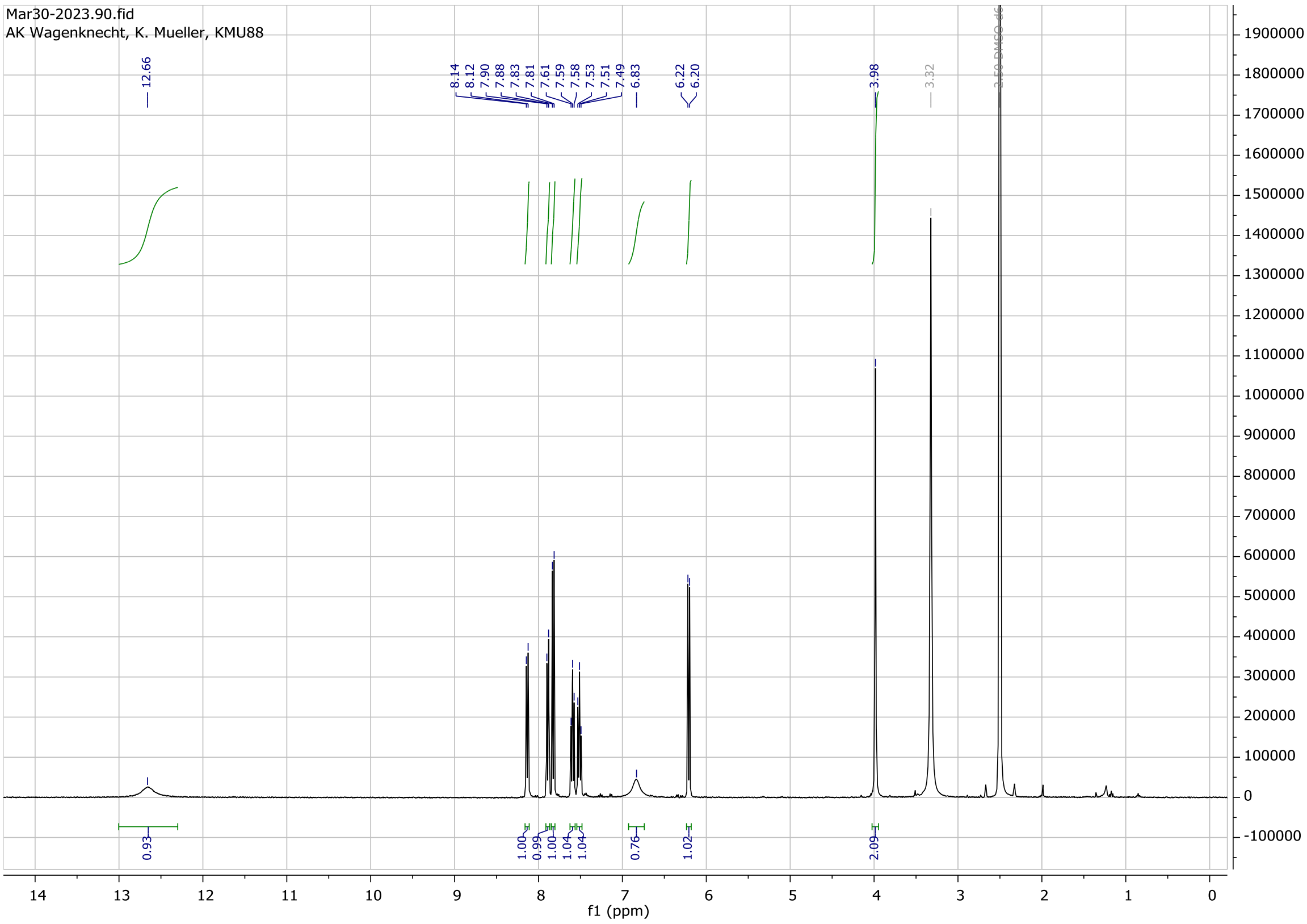

Figure S10. ^{1}**H-NMR** spectrum (400 MHz, DMSO-d_6) of **12**.

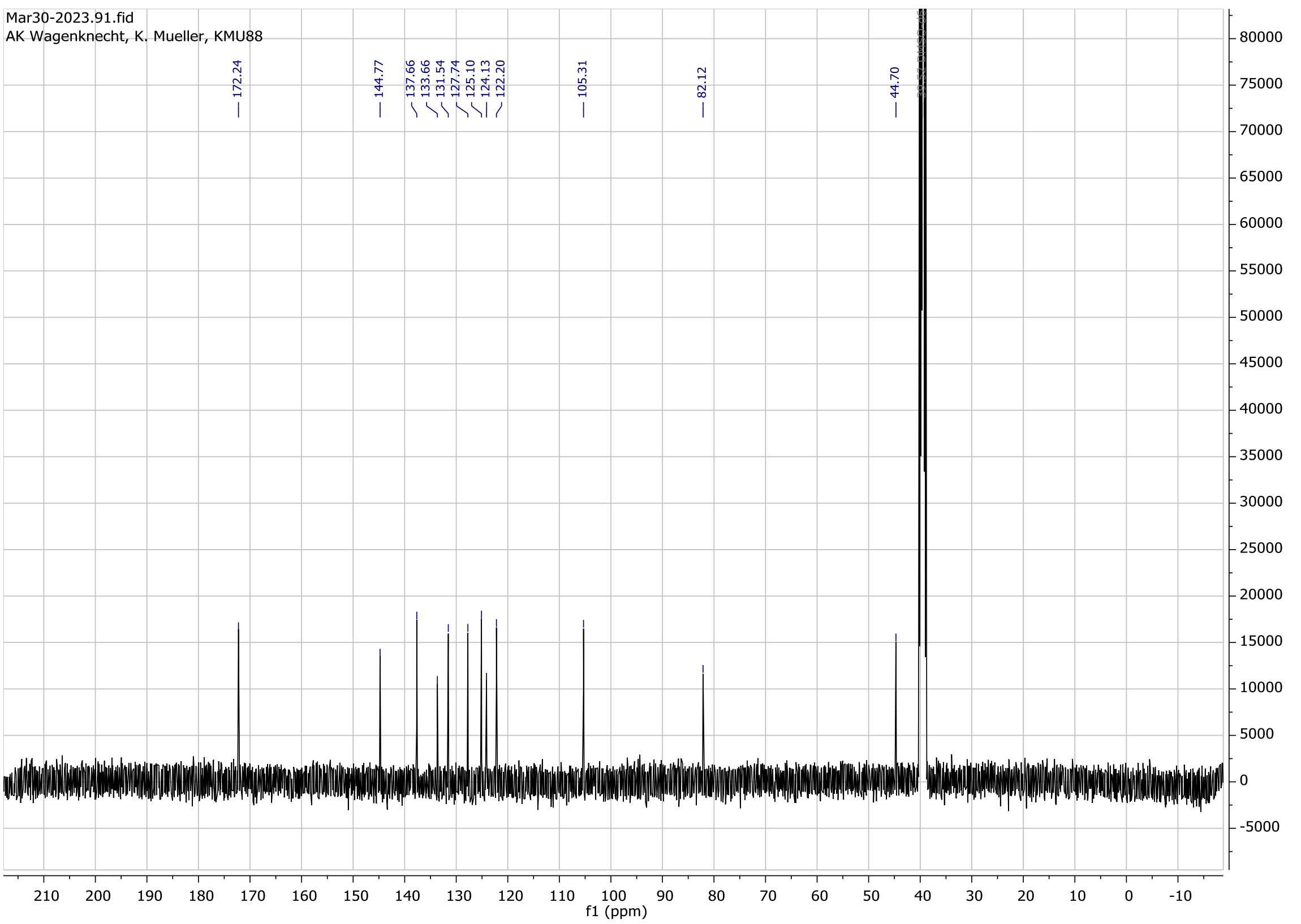

Figure S11. ^{13}C-NMR spectrum (126 MHz, DMSO-d_6) of **12**.

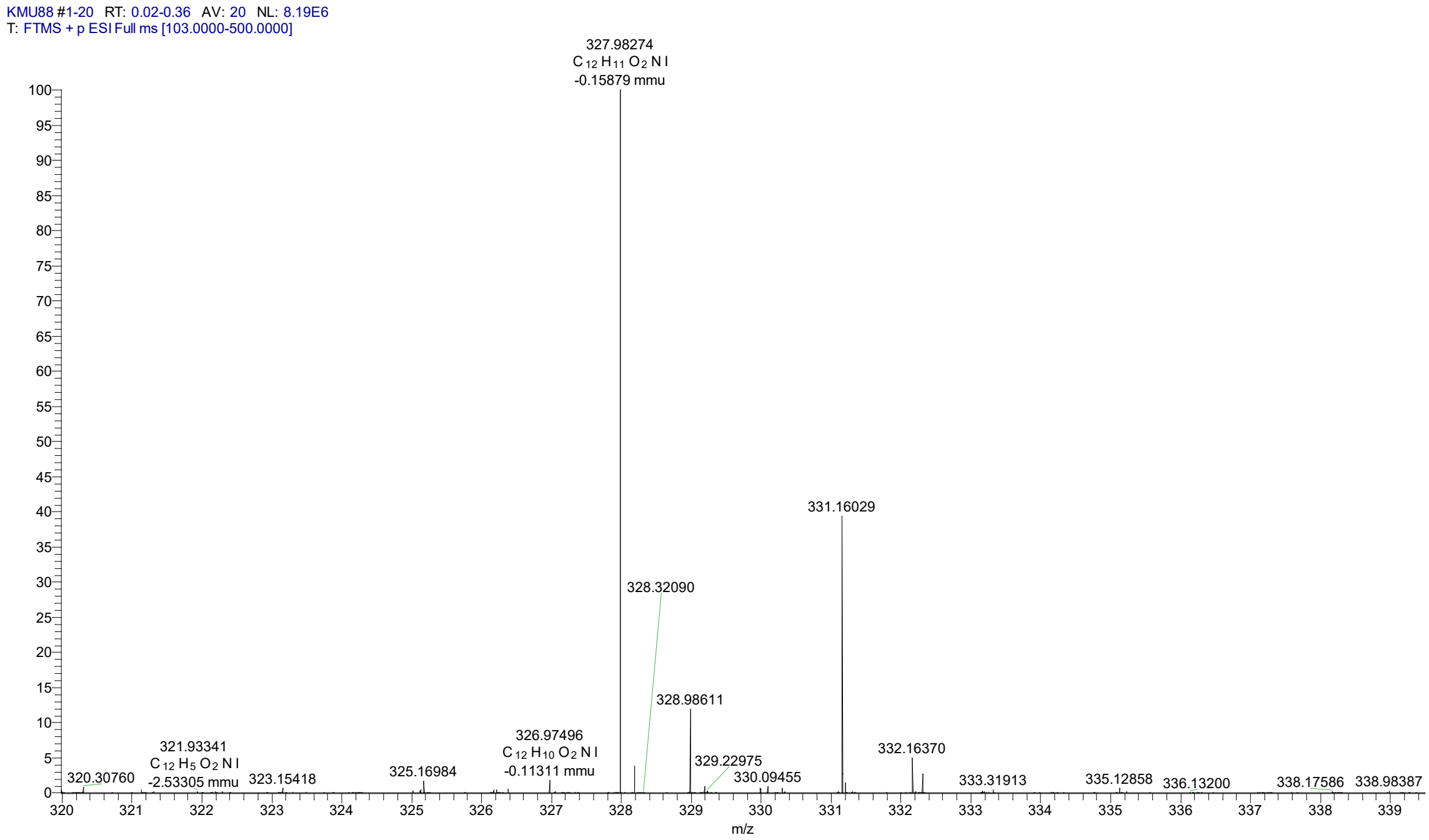

Figure S12. HR-MS analysis (ESI) of **12.**

Compound 13

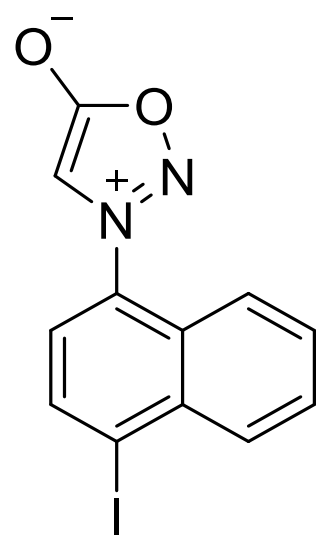

$C_{12}H_7IN_2O_2$
338.10 g/mol

In a heated Schlenk flask under argon, 3.99 g **12** (12.2 mmol, 1.00 eq.) was introduced and dissolved in 55 mL THF. Slowly 1.60 mL tert-butyl nitrite (1.38 g, 13.4 mmol, 1.10 eq.) was added to the reaction solution over 10 min. The reaction mixture was allowed to stir for 1 h at room temperature. 1.87 mL Trifluoroacetic anhydride (2.82 g, 13.4 mmol, 1.10 eq.) was then added over 10 min and the reaction solution was allowed to stir at room temperature for 1 h. EtOAc was added and the solution was quenched with saturated sodium bicarbonate solution. The aqueous phase was extracted three times with EtOAc. The combined organic phases were dried over sodium sulfate and the solvent was removed. The crude product was purified by automated column chromatography (CH_2Cl_2:MeOH 0-20 %). The product was obtained as a red-brown solid in a yield of 3.42 g (10.1 mmol, 83%).

^{1}H-NMR (400 MHz, $CDCl_3$-d_1): δ (ppm) = 8.28 (d, J = 7.9 Hz, 2H, arom.), 7.81 – 7.66 (m, 3H, arom.), 7.42 (d, J = 7.8 Hz, 1H, arom.), 6.68 (s, 1H, sydnone core).

^{13}C-NMR (126 MHz, $CDCl_3$-d_1): δ (ppm) = 168.7, 136.5, 135.3, 133.5, 132.1, 130.1, 129.9, 127.3, 124.0, 105.5, 98.6, 77.4.

HR-MS (ESI): m/z (calculated for $C_{12}H_8IN_2O_2^+$): 338.9625 [M+H]$^+$; found: 338.9624 [M+H]$^+$.

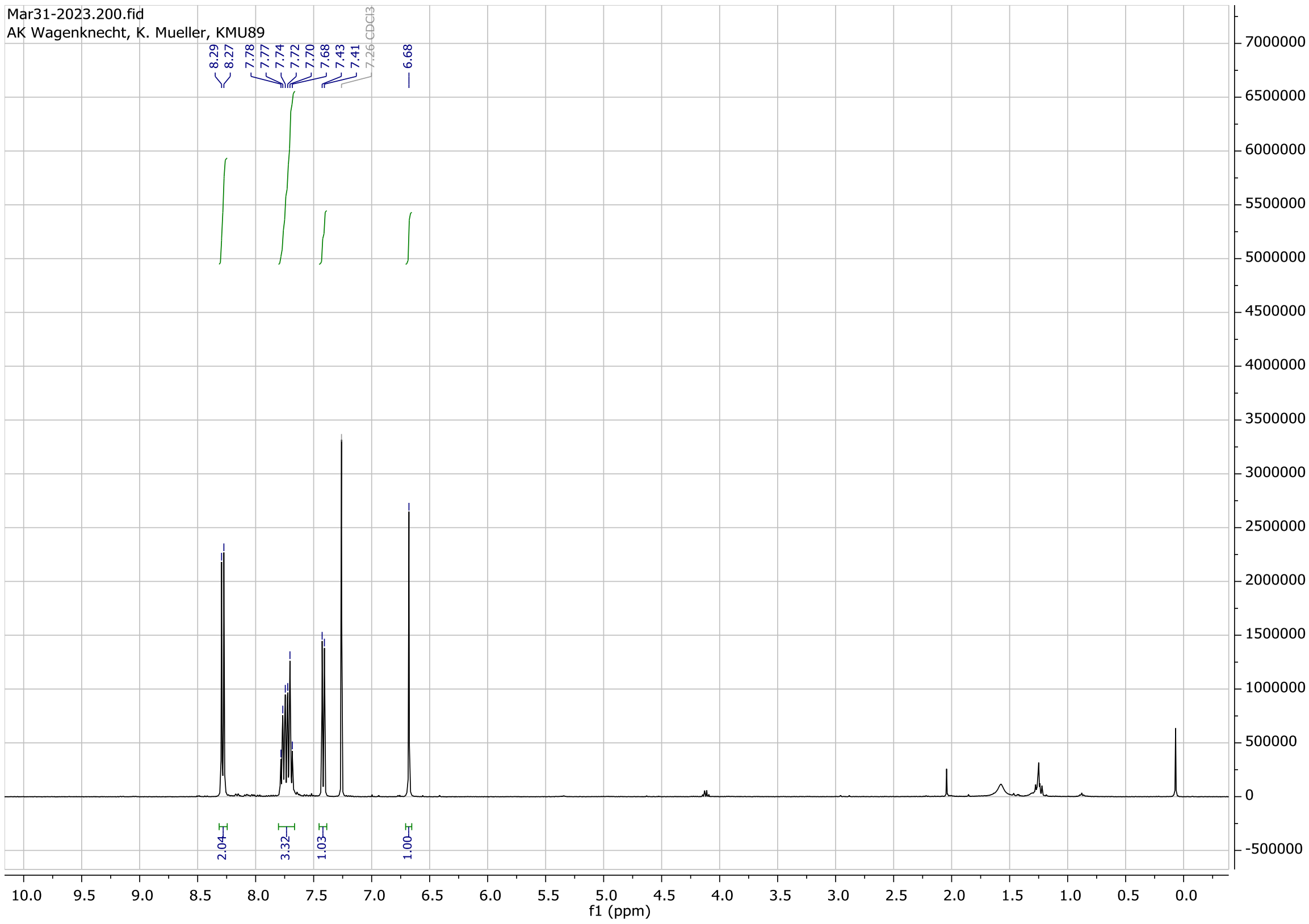

Figure S13. ^{1}H-NMR spectrum (400 MHz, $CDCl_3$-d_1) of **13**.

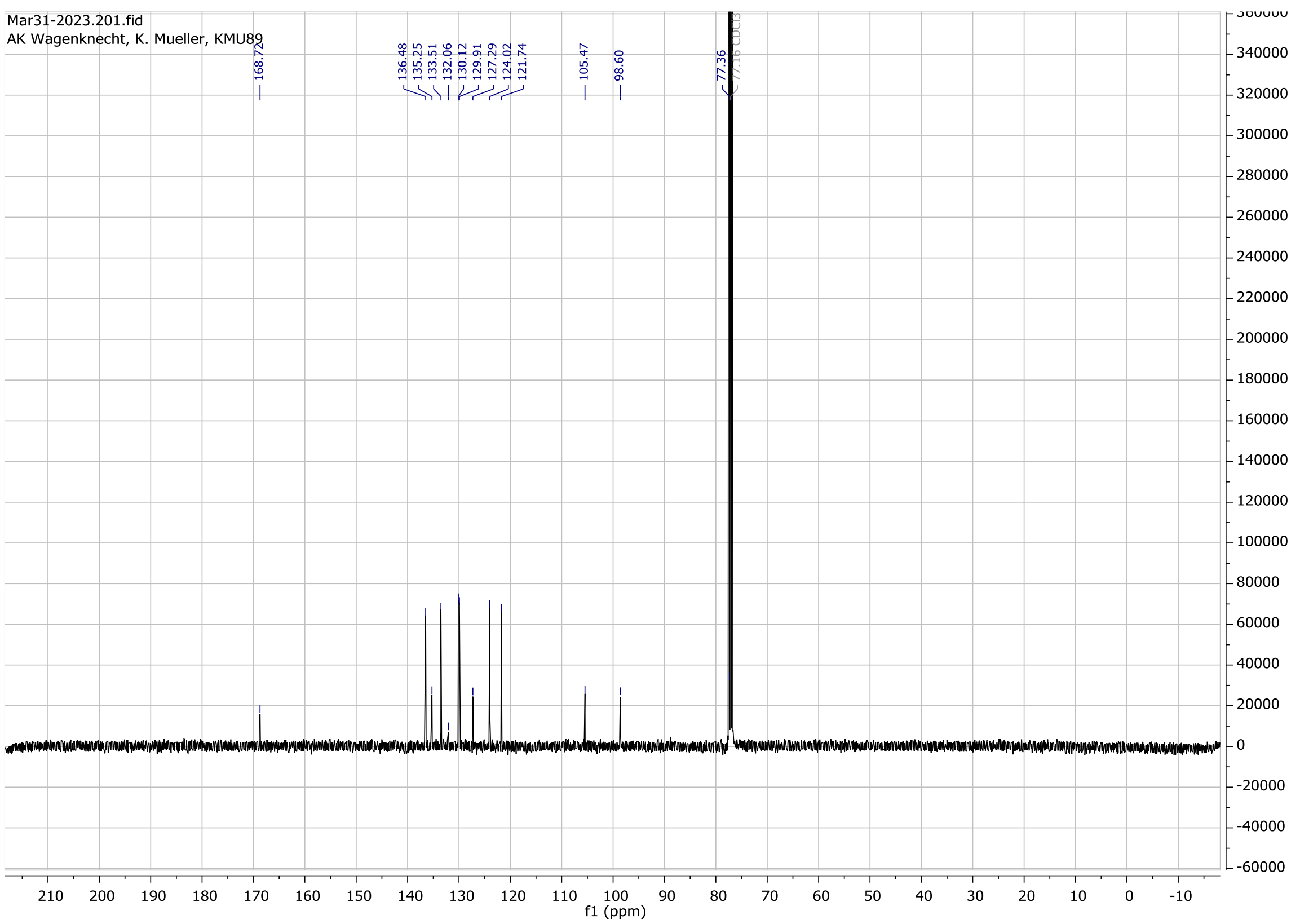

Figure S14. ^{13}C-NMR spectrum (126 MHz, $CDCl_3$-d_1) of **13**.

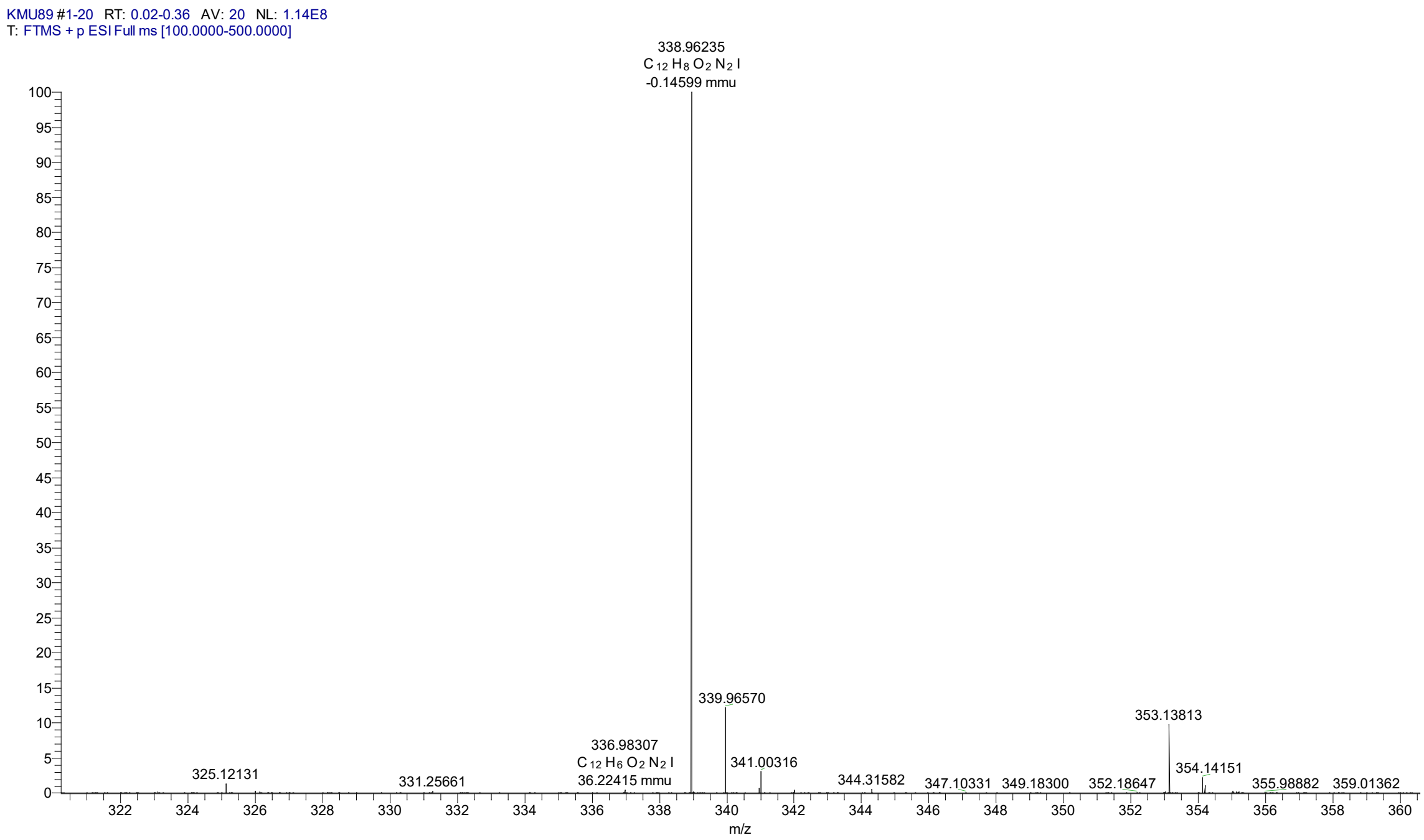

Figure S15. HR.MS analysis (ESI) of **13**.

Compound 14

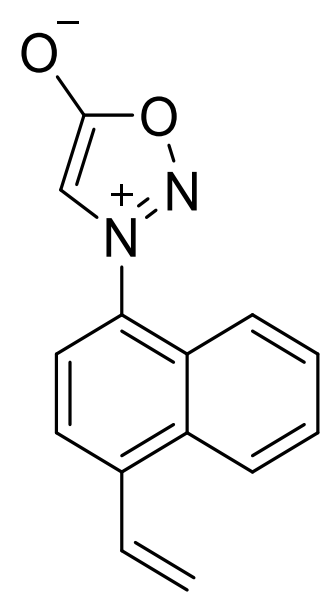

$C_{14}H_{10}N_2O_2$
238.25 g/mol

In a heated Schlenk flask under argon, 1.02 g **13** (3.00 mmol, 1.00 eq.) was introduced and dissolved in 70 mL DMF. To the reaction solution, 0.382 g LiCl (9.01 mmol, 3.00 eq.), 0.105 g $Pd(PPh_3)_2Cl_2$ (0.15 mmol, 0.05 eq.) and 0.60 mL tetravinyltin (0.750 g, 3.31 mmol, 1.10 eq.) were added. The reaction mixture was allowed to stir overnight at 65 °C. The solvent was then removed. The residue was dissolved in CH_2Cl_2 and 1 M NaOH solution and allowed to stir for 1 h. The aqueous phase was extracted three times with CH_2Cl_2. The combined organic phases were dried over Na_2SO_4. The solvent was removed. The crude product was purified by automated column chromatography (CH_2Cl_2:MeOH 0-10 %). The product was obtained as an orange solid in a yield of 0.715 g (3.00 mmol, 100%).

^{1}H-NMR (400 MHz, $CDCl_3$-*d₁*): δ (ppm) = 8.29 – 8.21 (m, 1H, arom.), 7.78 – 7.73 (m, 1H, arom.), 7.72 (s, 1H, arom.), 7.69 (tt, *J* = 5.6, 3.0 Hz, 3H, arom.), 7.49 (dd, *J* = 17.3, 11.0 Hz, 1H, R-CH=CH_2), 7.47 (d, *J* = 11.0 Hz, 1H, arom.), 6.67 (s, 1H, sydnone core), 5.91 (dd, *J* = 17.3, 0.9 Hz, 1H, R-CH=CH_2), 5.69 (dd, *J* = 11.0, 0.9 Hz, 1H, R-CH=CH_2).

^{13}C-NMR (126 MHz, $CDCl_3$-d_1): δ (ppm) = 168.9, 141.0, 133.2, 131.8, 129.0, 128.1, 127.1, 125.0, 123.4, 122.7, 121.5, 120.7, 98.6, 77.4.

HR-MS (ESI): m/z (calculated for $C_{14}H_{11}N_2O_2^+$): 239.0815 $[M+H]^+$; found: 239.0825 $[M+H]^+$.

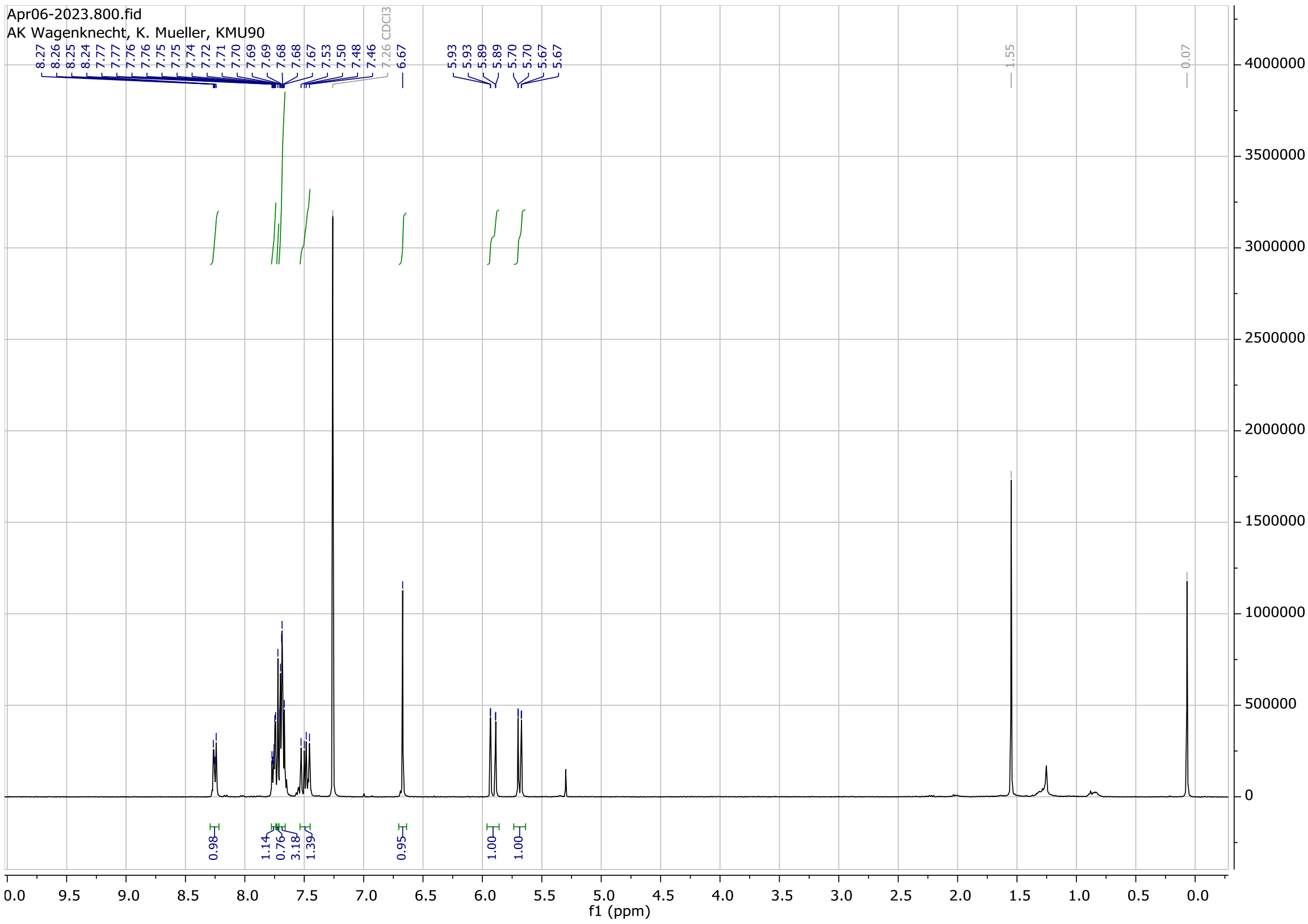

Figure S16. ^1H-NMR spectrum (400 MHz, $CDCl_3$-d_1) of **14**.

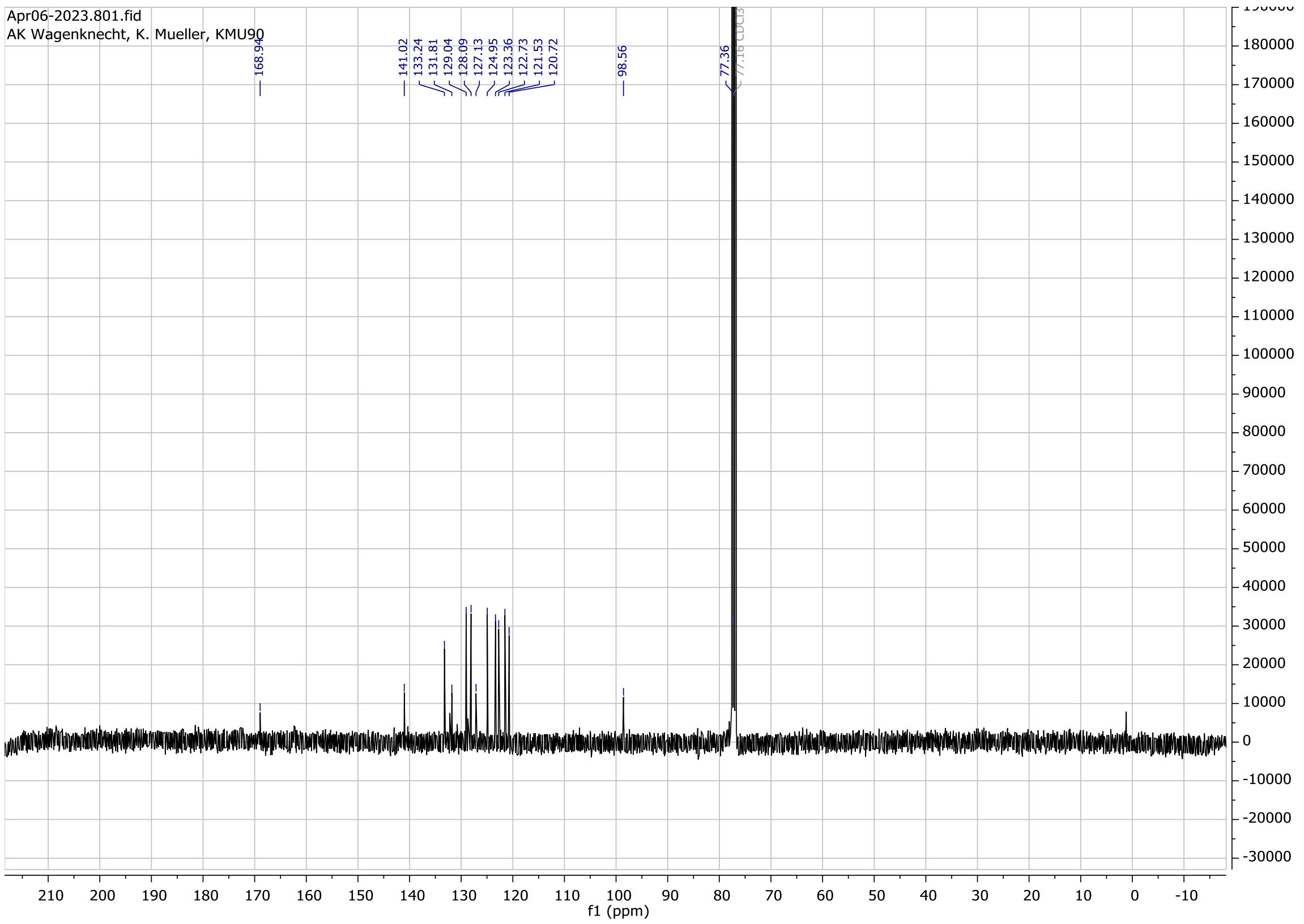

Figure S17. ^{13}C-NMR spectrum (126 MHz, $CDCl_3$-d_1) of **14**.

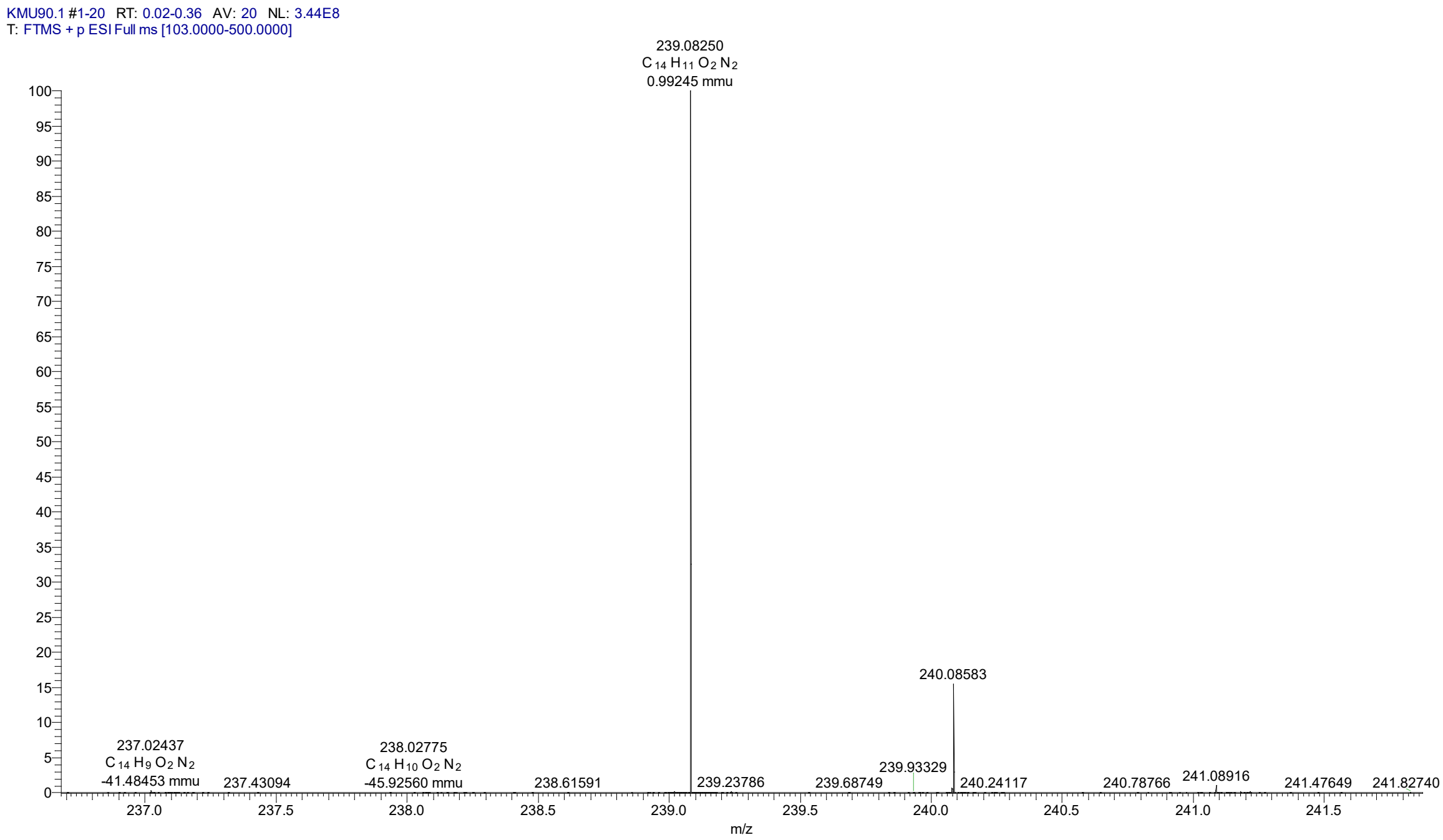

Figure S18. HR-MS analysis (ESI) of **14**.

Compound 3

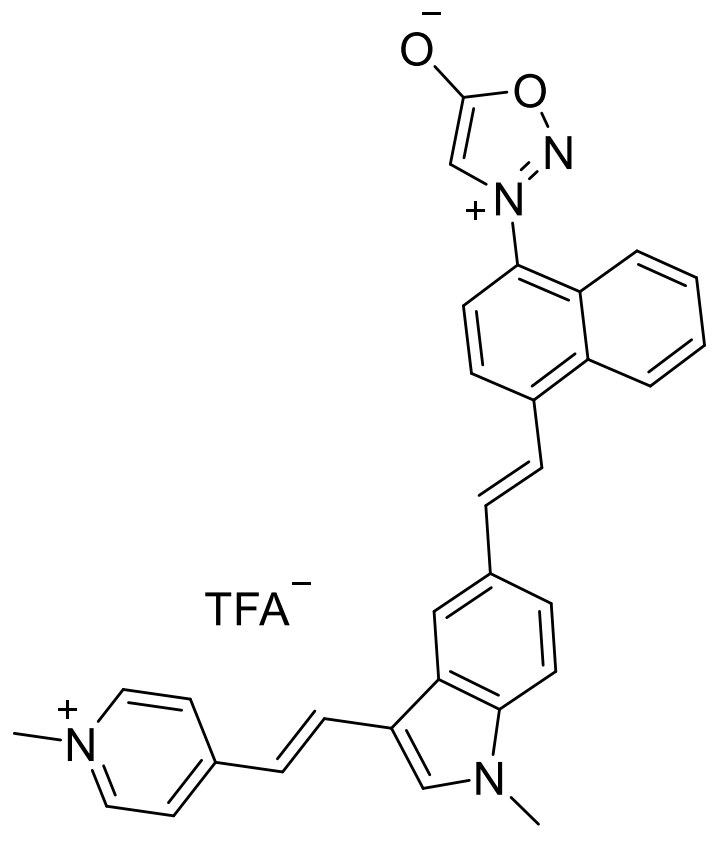

$C_{31}H_{25}N_4O_2^+C_2F_3O_2^-$
598.58 g/mol

In a heated 10 mL crimp vial under argon, 0.100 g **7**[31] (0.220 mmol, 1.00 eq.), 0.010 g $Pd_2(dba)_3$ (0.010 mmol, 0.05 eq.), 0.031 g Qphos (0.040 mmol, 0.20 eq.) and 0.105 g **14** (0.440 mmol, 2.00 eq.) dried under high vacuum. The solids were then dissolved with 2.00 mL dry DMF and 0.15 mL Et_3N (0.111 g, 1.10 mmol, 5.00 eq.) was added. The reaction was stirred in a metal heating block at 80 °C for 18 h. After the solution had cooled to room temperature, Et_2O was added until no further precipitate was formed. The suspension was transferred into reaction tubes. The solution was centrifuged and the supernatant was pipetted off and discarded. Subsequently, Et_2O was added and the suspension was vortexed and centrifuged again in the next step and the supernatant discarded. This was carried out two more times. The residue was dried in high vacuum and purified via reversed-phase column chromatography. The product was obtained as a brown solid in a yield of 0.079 g (0.132 mmol, 60%).

^{1}H-NMR (500 MHz, DMSO-*d*$_6$): δ (ppm) = 8.76 – 8.70 (m, 3H, arom.), 8.50 (s, 1H, arom.), 8.24 (dd, *J* = 16.1, 12.6 Hz, 2H, R-CH=CH-R, arom.), 8.14 (dd, *J* = 14.5, 7.4 Hz, 3H, R-CH=CH-R, arom.), 8.06 (d, *J* = 7.8 Hz, 1H, arom.), 8.00 (s, 1H, arom.), 7.94 (d, *J* = 8.6 Hz, 1H, arom.), 7.82 (dtd, *J* = 16.0, 8.0, 3.8 Hz, 3H, R-CH=CH-R, arom.), 7.74 (s, 1H, arom.), 7.70 – 7.63 (m, 2H, arom.), 7.38 (d, *J* = 16.2 Hz, 1H, R-CH=CH-R), 4.19 (s, 3H, R-CH_3), 3.93 (s, 3H, R-CH_3).

^{13}C-NMR (126 MHz, DMSO-*d*$_6$): δ (ppm) = 168.4, 157.8, 157.6, 154.0, 144.3, 139.7, 138.1, 136.0, 135.6, 135.2, 131.0, 130.5, 129.7, 128.9, 127.8, 126.9, 125.8, 125.0, 124.2, 122.2, 121.8, 121.7, 117.3, 113.1, 111.5, 99.8, 46.3, 33.3.

HR-MS (ESI): m/z (calculated for $C_{31}H_{25}N_4O_2^+$): 485.1972 [M]$^+$; found: 485.1968 [M]$^+$

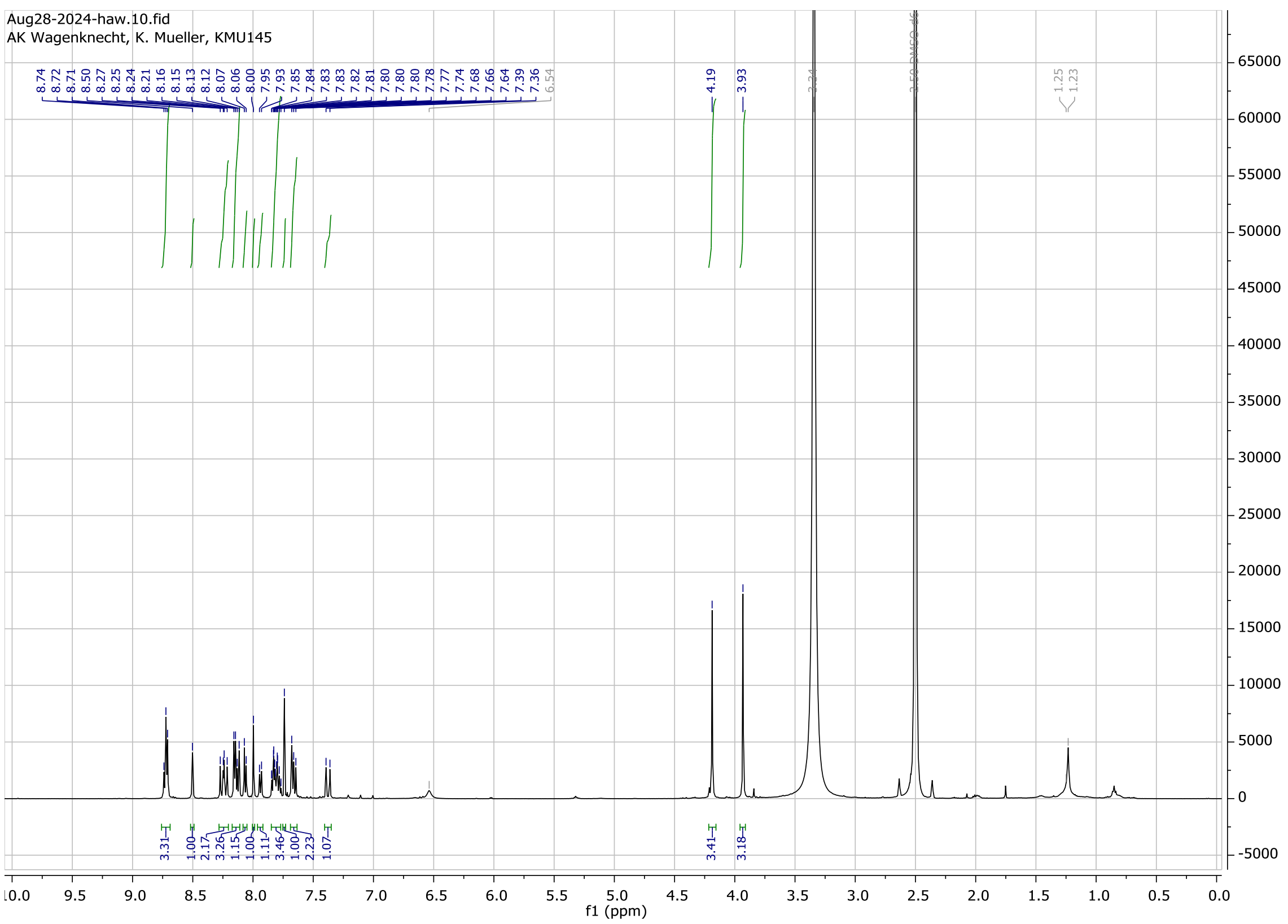

Figure S19. ^{1}H-NMR spectrum (500 MHz, DMSO-d_6) of **3**.

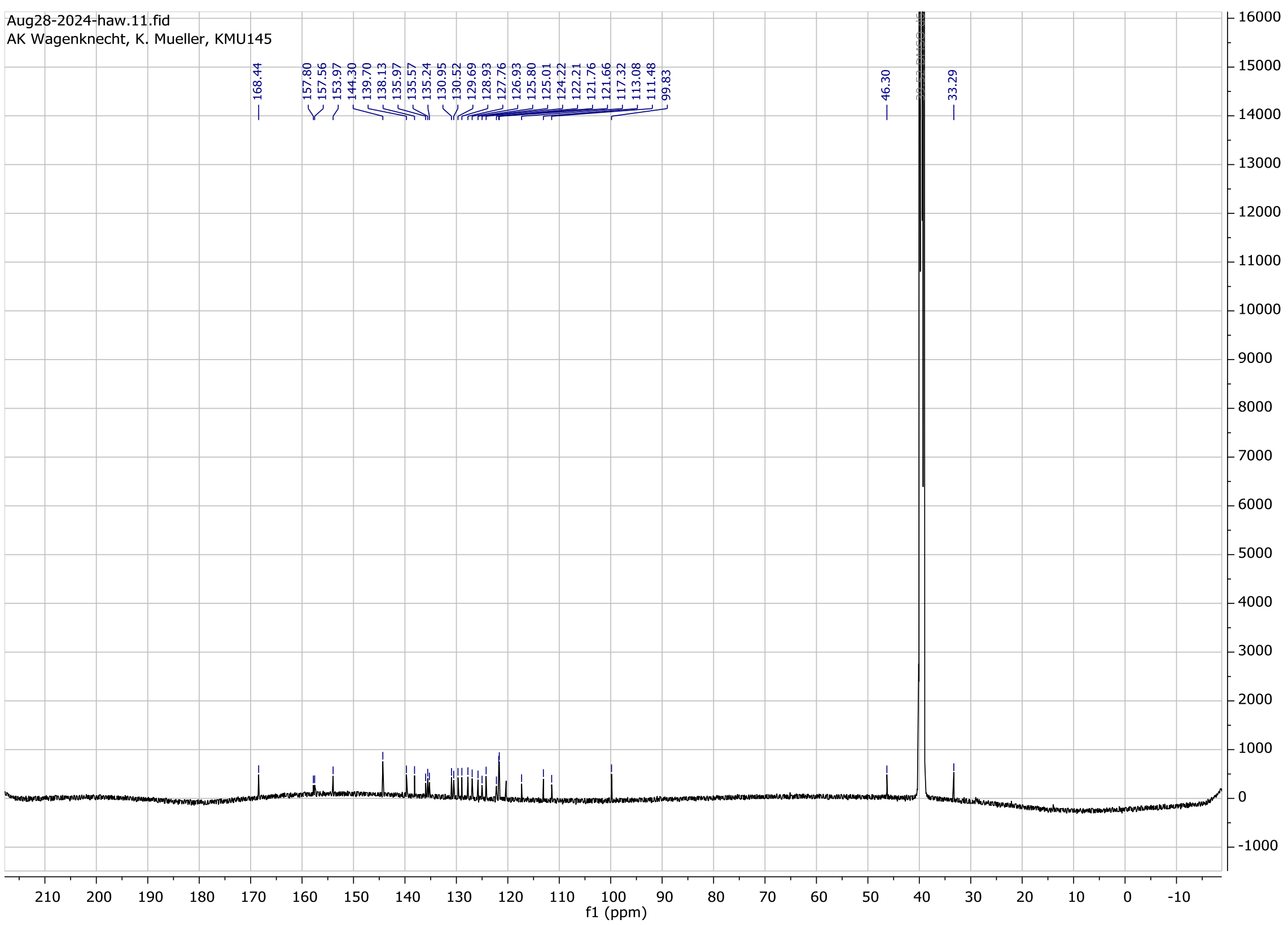

Figure S20. ^{13}C-NMR spectrum (126 MHz, DMSO-d_6) of **3**.

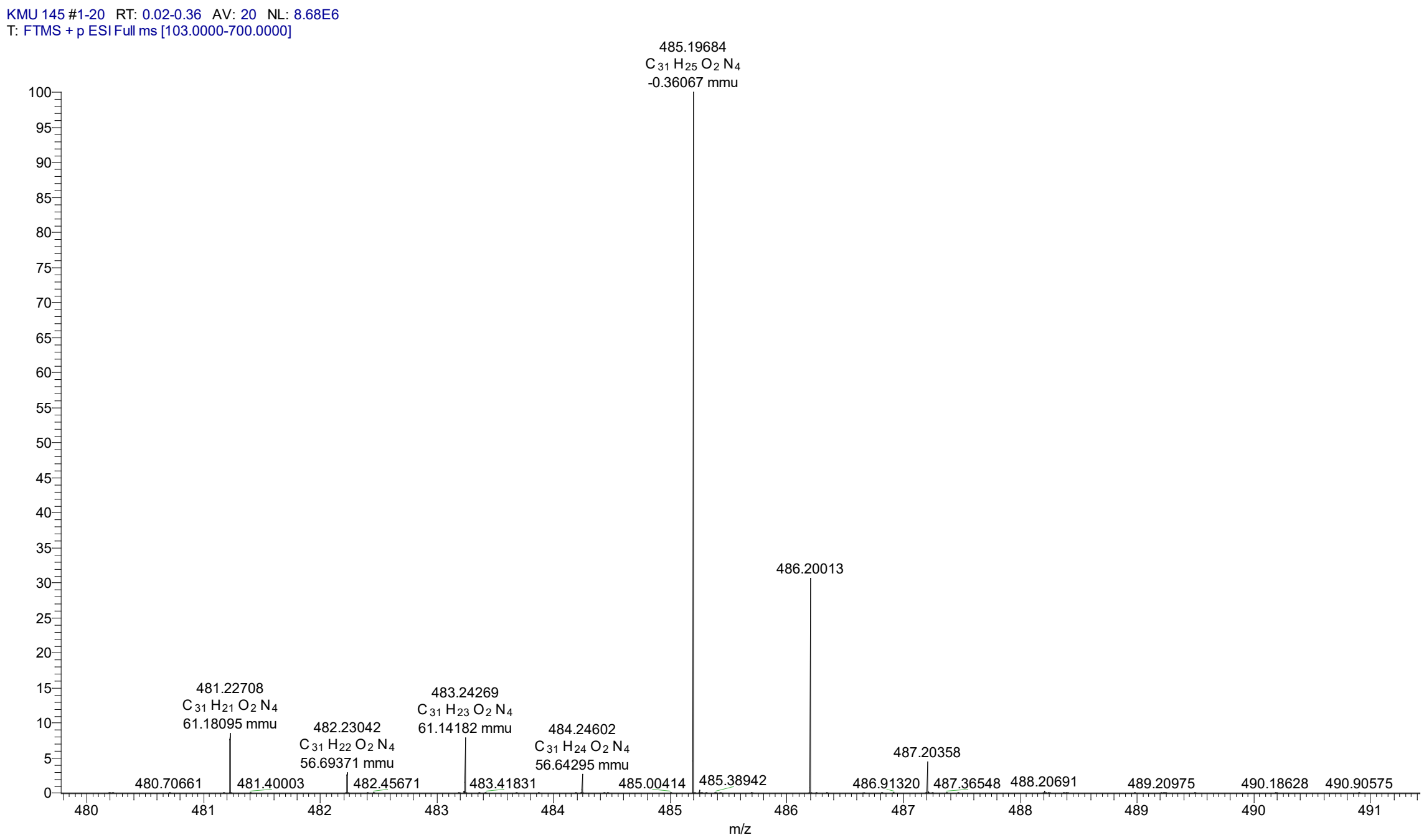

Figure S21. HR-MS analysis (ESI) of **3**.

Compound 4

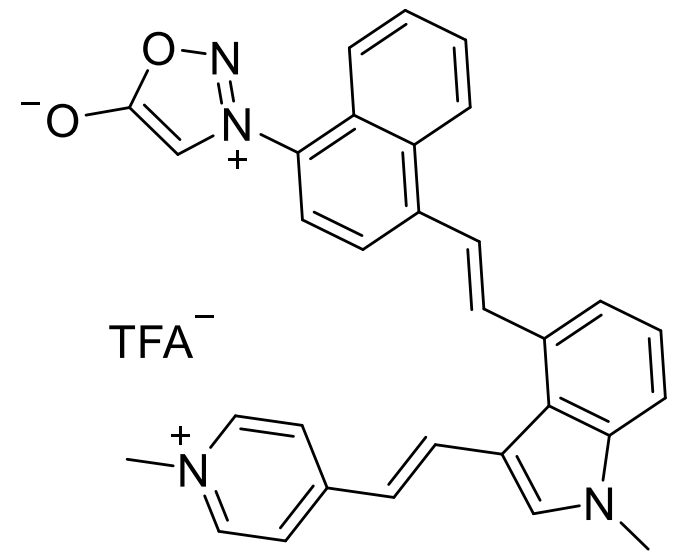

$C_{31}H_{25}N_4O_2{}^+C_2F_3O_2{}^-$
598.58 g/mol

In a heated 10 mL crimp vial under argon, 0.100 g **8**[30] (0.220 mmol, 1.00 eq.), 0.010 g $Pd_2(dba)_3$ (0.010 mmol, 0.05 eq.), 0.031 g Qphos (0.040 mmol, 0.20 eq.) and 0.105 g **14** (0.440 mmol, 2.00 eq.) dried under high vacuum. The solids were then dissolved with 2.00 mL dry DMF and 0.15 mL Et_3N (0.111 g, 1.10 mmol, 5.00 eq.) was added. The reaction was stirred in a metal heating block at 80 °C for 18 h. After the solution had cooled to room temperature, Et_2O was added until no further precipitate was formed. The suspension was transferred into reaction tubes. The solution was centrifuged and the supernatant was pipetted off and discarded. Subsequently, Et_2O was added and the suspension was vortexed and centrifuged again in the next step and the supernatant discarded. This was carried out two more times. The residue was dried in high vacuum and purified via reversed-phase column chromatography. The product was obtained as a brown solid in a yield of 0.059 g (0.099 mmol, 45%).

^{1}H-NMR (500 MHz, DMSO-d_6): δ (ppm) = 8.70 – 8.63 (m, 3H, arom.), 8.48 (d, *J* = 15.9 Hz, 1H, R-CH=CH-R), 8.25 (dd, *J* = 22.0, 7.1 Hz, 3H, R-CH=CH-R, arom.), 8.12 – 8.05 (m, 2H, R-CH=CH-R, arom.), 8.02 (d, *J* = 6.9 Hz, 2H, arom.), 7.87 – 7.83 (m, 1H, arom.), 7.80 (d, *J* = 7.5 Hz, 1H, arom.),

7.79 – 7.74 (m, 3H, R-CH=CH-R, arom.), 7.60 (d, *J* = 8.2 Hz, 1H, arom.), 7.39 (t, *J* = 7.9 Hz, 1H, arom.), 7.13 (d, *J* = 15.9 Hz, 1H, R-CH=CH-R), 4.17 (s, 3H, R-CH_3), 3.94 (s, 3H, R-CH_3).

^{13}C-NMR (126 MHz, DMSO-d_6): δ (ppm) = 168.4, 157.7, 157.5, 153.2, 144.4, 139.3, 138.1, 136.0, 132.8, 132.6, 131.0, 130.8, 130.0, 129.0, 128.0, 126.9, 125.9, 124.8, 124.1, 123.9, 122.7, 122.5, 122.0, 121.7, 120.2, 118.8, 113.2, 111.2, 99.9, 46.4, 33.4.

HR-MS (ESI): m/z (calculated for $C_{31}H_{25}N_4O_2^+$): 485.1972 [M]$^+$; found: 485.1969 [M]$^+$

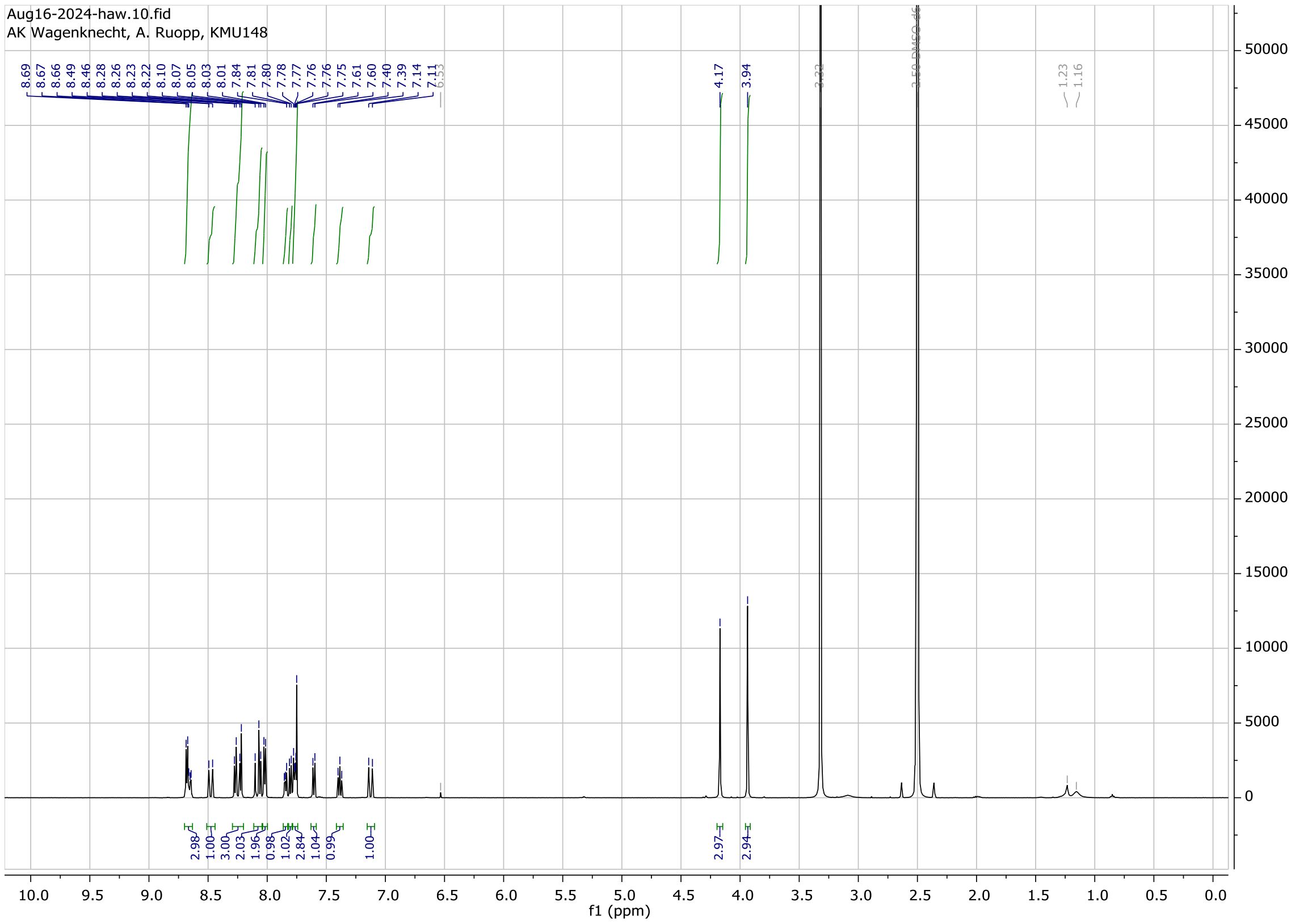

Figure S22. ^{1}H-NMR spectrum (500 MHz, DMSO-d_6) of **4**.

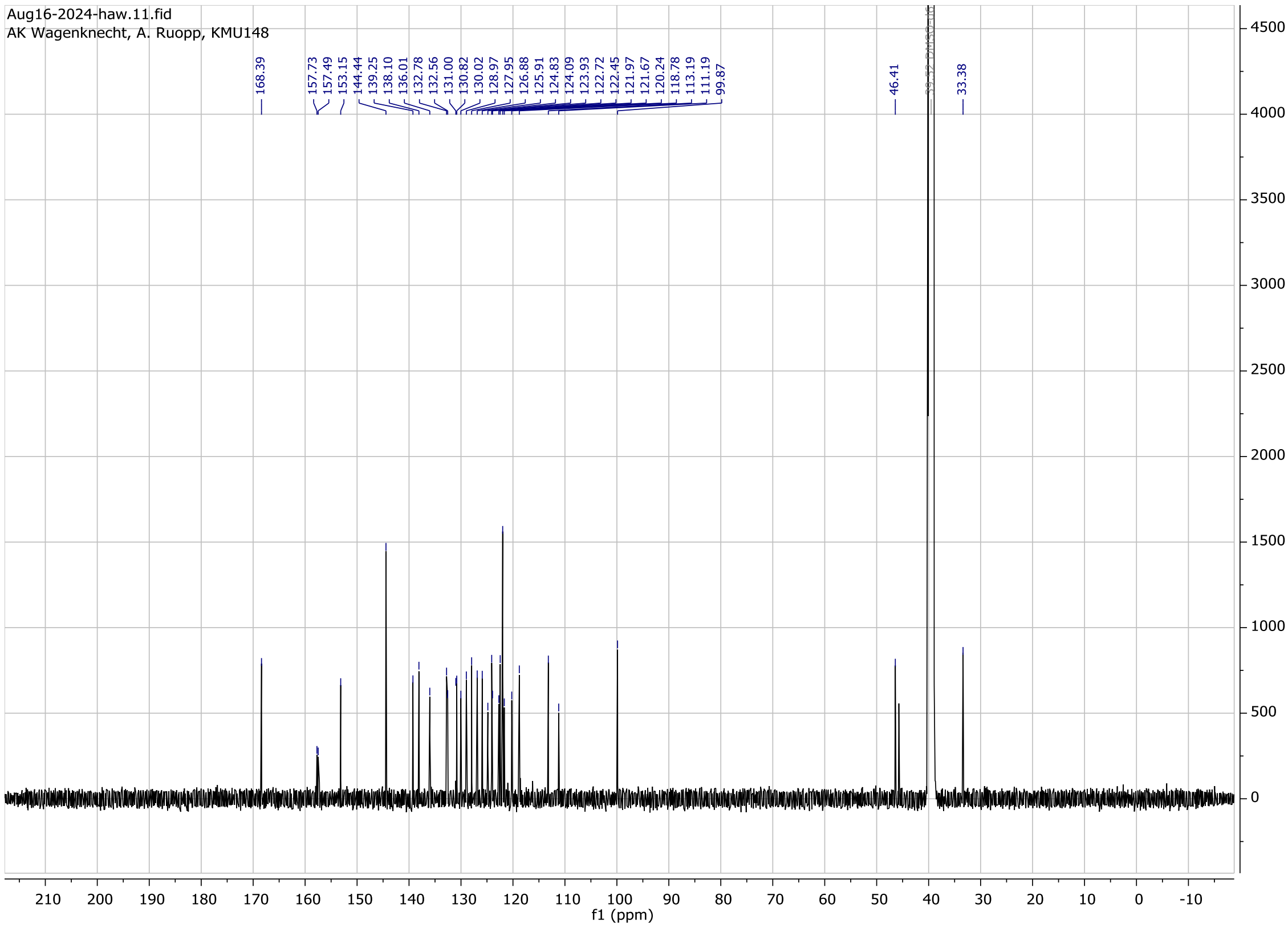

Figure S23. ^{13}C-NMR spectrum (126 MHz, DMSO-d_6) of **4**.

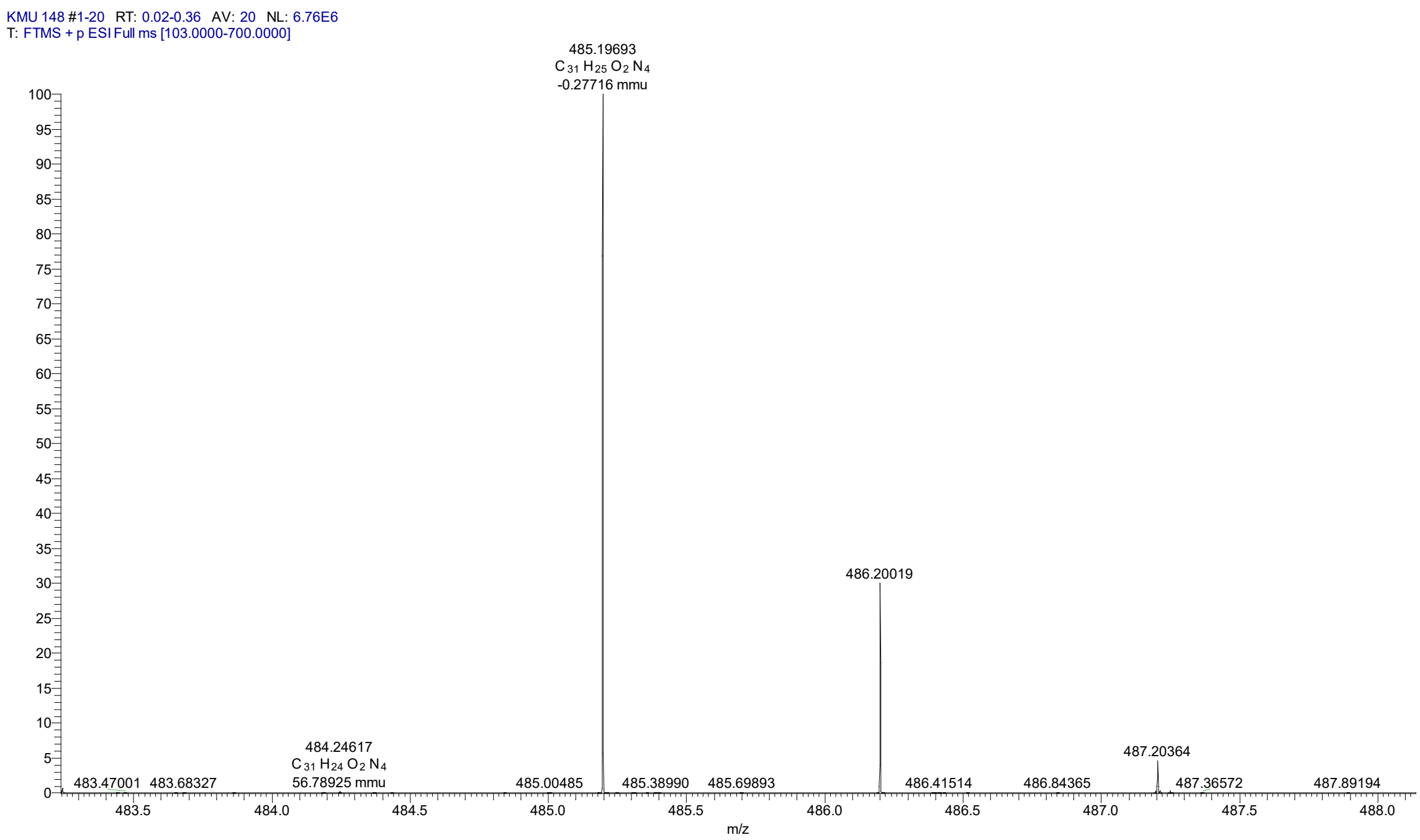

Figure S24. HR-MS analysis (ESI) of **4**.

Compound 5

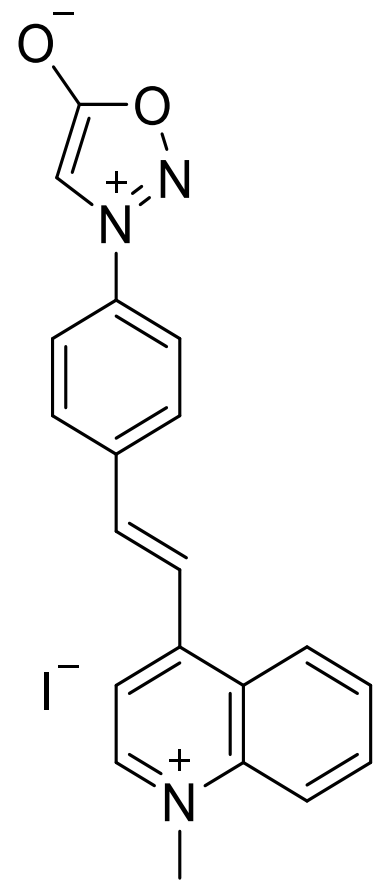

$C_{20}H_{16}N_3O_2^{+}I^{-}$
457.28 g/mol

In accordance with a literature prescription to a solution of 0.088 g **17**[31] (0.310 mmol, 1.00 eq.) and 0.065 g **15**[25] (0.340 mmol, 1.10 eq.) in 8 mL MeOH was added three drops of piperidine. The mixture was stirred at 75 °C for 24 h. Et_2O was added and the mixture was cooled to 0 °C and stirred for 1 h, after which the precipitate was filtered. The precipitate was dried under reduced pressure. The product was obtained as a dark violet solid in a yield of 0.071 g (0.155 mmol, 50%)

^{1}H-NMR (400 MHz, DMSO-*d₆*): δ (ppm) = 9.45 (d, *J* = 6.4 Hz, 1H, arom.), 9.09 (dd, *J* = 8.8, 1.3 Hz, 3H, arom.), 8.59 – 8.47 (m, 3H, arom.), 8.35 – 8.29 (m, 3H, arom.), 8.23 (d, *J* = 16.1 Hz, 1H, R-CH=CH-R), 8.16 – 8.09 (m, 3H, arom), 7.90 (s, 1H, sydnone core), 4.59 (s, 3H, N-CH_3).

^{13}C-NMR (101 MHz, DMSO-d_6): δ (ppm) = 168.5, 151.9, 148.6, 140.2, 139.2, 138.8, 135.2, 130.1 (2C), 129.6, 129.5, 126.60, 126.56, 123.1, 122.0 (2C), 119.5, 117.1, 94.9, 45.0.

HR-MS (ESI): m/z (calculated for $C_{20}H_{16}N_3O_2^+$): 330.1237 [M]$^+$; found: 330.1230 [M]$^+$.

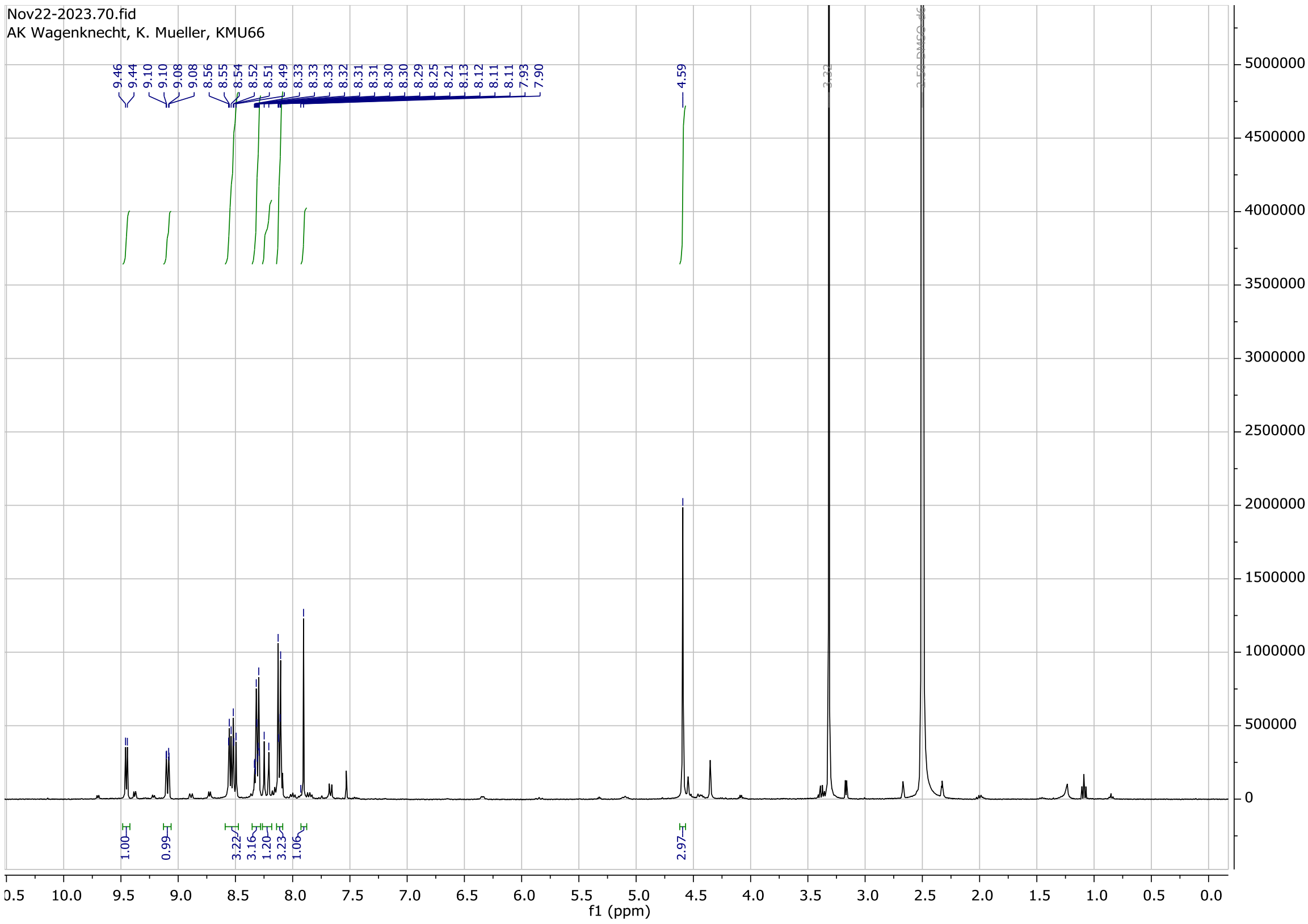

Figure S25. ^{1}H-NMR spectrum (400 MHz, DMSO-d_6) of **5**.

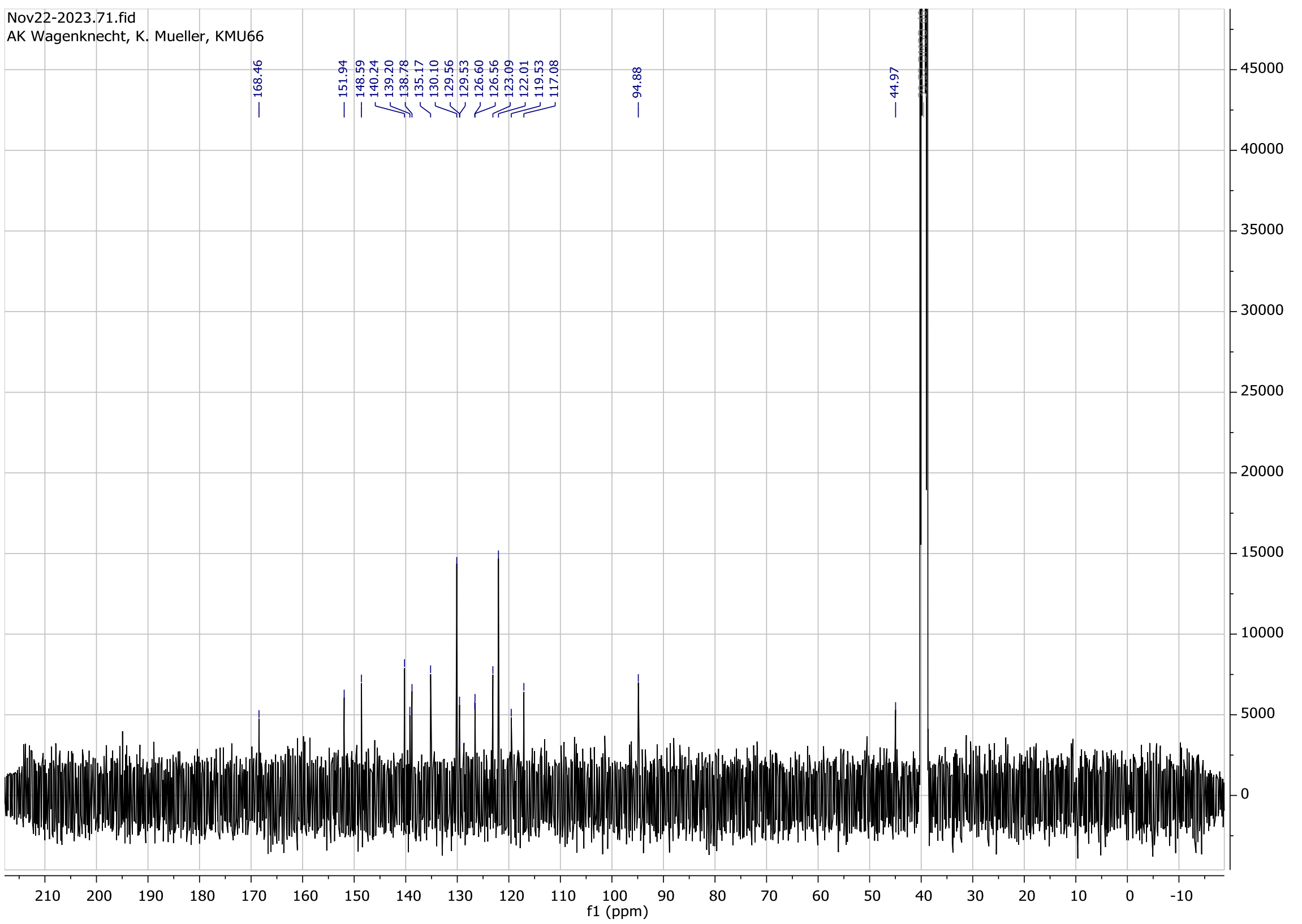

Figure S26. ^{13}C-NMR spectrum (101 MHz, DMSO-d_6) of **5**.

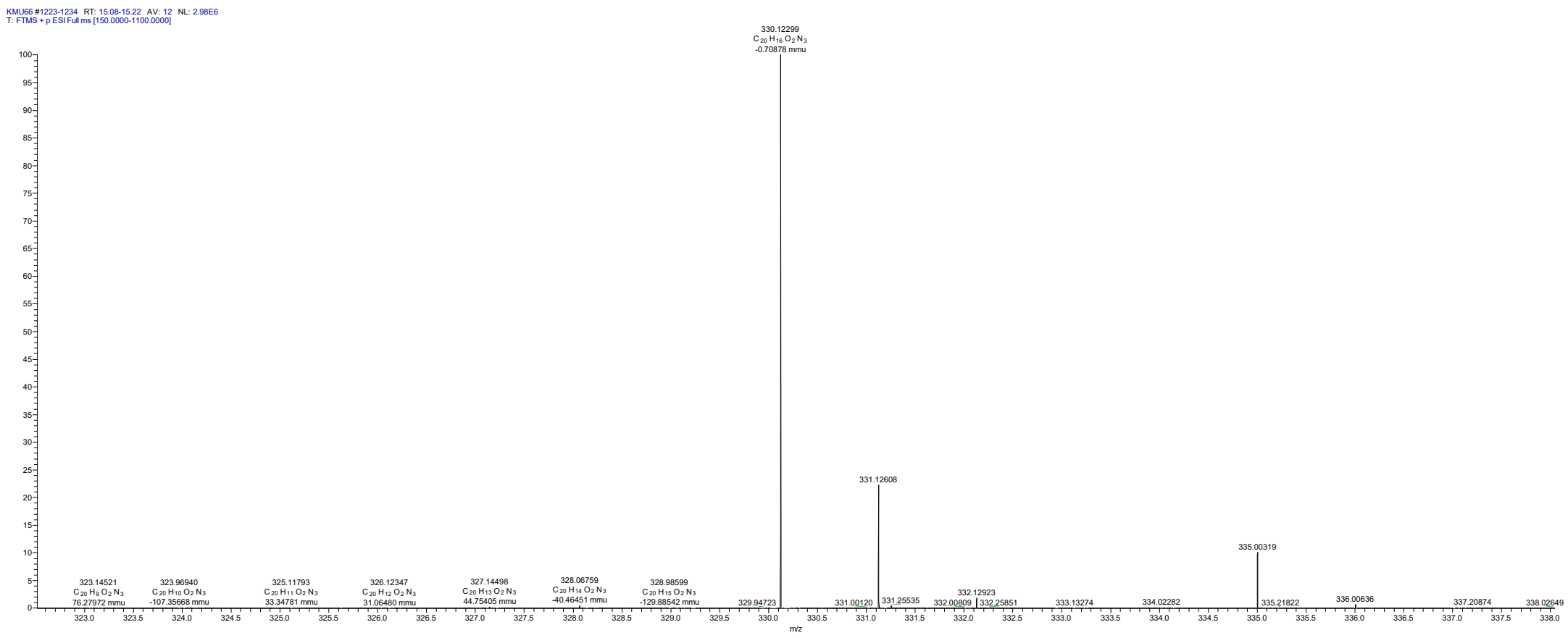

Figure S27. HR-MS analysis (ESI) of **5**.

Compound 22

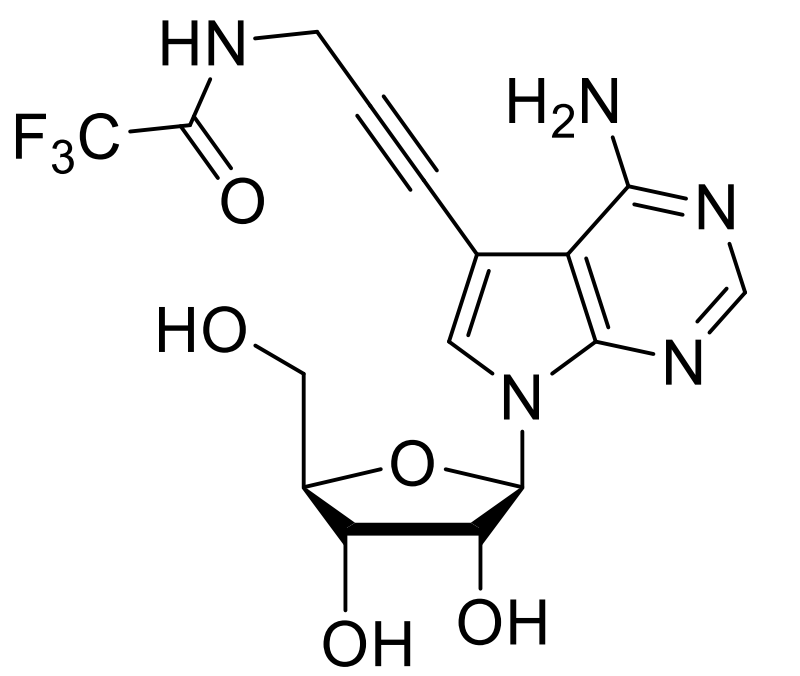

$C_{16}H_{16}F_3N_5O_5$
415.33 g/mol

In a heated round bottom flask under argon atmosphere, 1.88 g **21**[34] (4.78 mmol, 1.00 eq.), 2.17 g **19**[33] (14.3 mmol, 3.00 eq.), 0.200 g CuI (1.05 mmol, 0.22 eq.) and 1.33 mL Et_3N (0.968 g, 9.56 mmol, 2.00 eq.) were dissolved in 30 mL dry DMF. The solution was degassed and then 0.553 g $Pd(PPh_3)_4$ (0.48 mmol, 0.10 eq.) was added. The reaction mixture was stirred at room temperature for 24 h and the solvent was removed under reduced pressure. The residue was purified by column chromatography (CH_2Cl_2:MeOH 10 – 50 %). The product was obtained as a light brown solid in a yield of 1.39 g (3.35 mmol, 70 %).

^{1}H-NMR (400 MHz, DMSO-d_6): δ (ppm) = 10.1 (t, *J* = 5.5 Hz, 1H, N*H*), 8.11 (s, 1H, H-2), 7.77 (s, 1H, H-8), 6.84 (br s, 2H, N*H₂*), 6.01 (d, *J* = 6.1 Hz, 1H, H-1'), 5.33 (d, *J* = 6.3 Hz, 1H, 3'-OH), 5.19 (t, *J* = 5.6 Hz, 1H, 5'-OH), 5.11 (d, *J* = 4.8 Hz, 1H, 2'-OH), 4.36 (q, *J* = 6.1 Hz, 1H, H-3'), 4.31 (d, *J* = 5.4 Hz, 2H, C*H₂*), 4.10 – 4.03 (m, 1H, H-4'), 3.89 (q, *J* = 3.6 Hz, 1H, H-2'), 3.66 – 3.58 (m, 1H, H-5$_a$'), 3.53 (ddd, *J* = 11.9, 6.1, 3.6 Hz, 1H, H-5$_b$').

^{13}C-NMR (126 MHz, DMSO-d_6): δ (ppm) = 157.5, 152.7, 149.6, 127.0, 112.2, 102.3, 94.1, 91.0, 87.3, 86.8, 85.2, 76.1, 74.0, 70.5, 61.5, 29.9.

HR-MS (ESI): m/z (calculated for $C_{16}H_{17}F_3N_5O_5^+$): 416.1176 [M+H]$^+$; found: 416.1172 [M+H]$^+$.

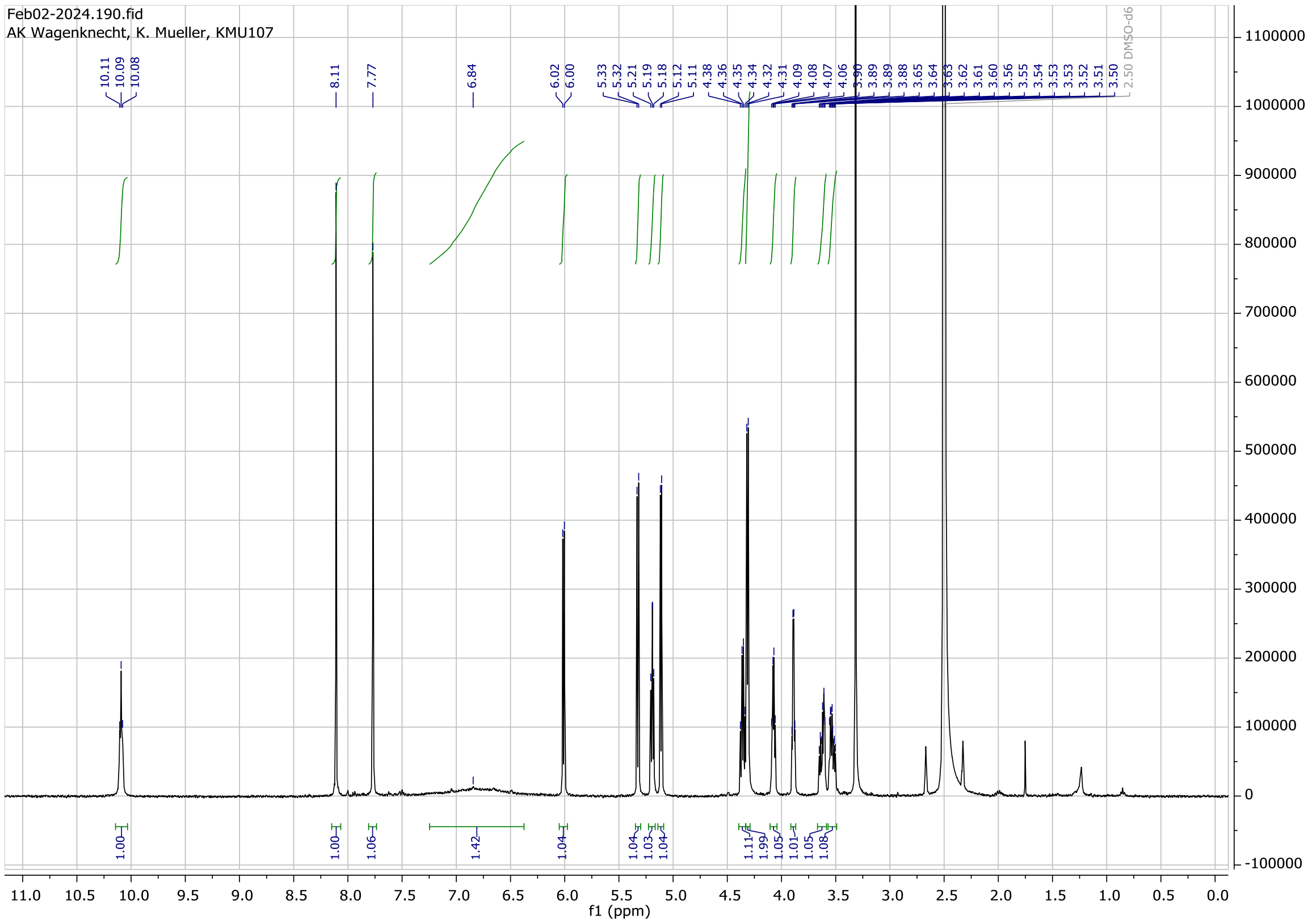

Figure S28: ^{1}H-NMR spectrum (400 MHz, DMSO-d_6) of **22**.

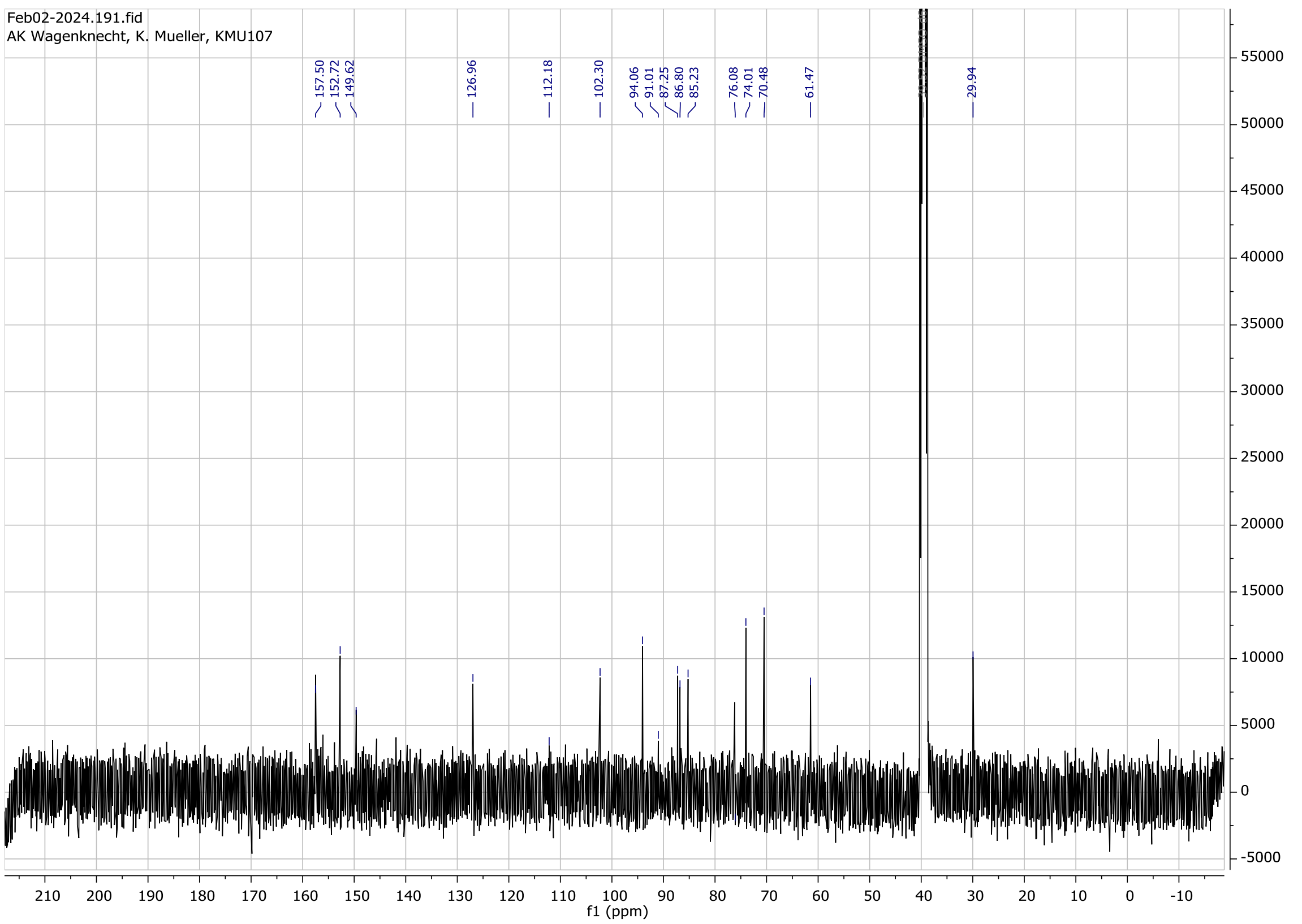

Figure S29: ^{13}C-NMR spectrum (126 MHz, DMSO-d_6) of **22**.

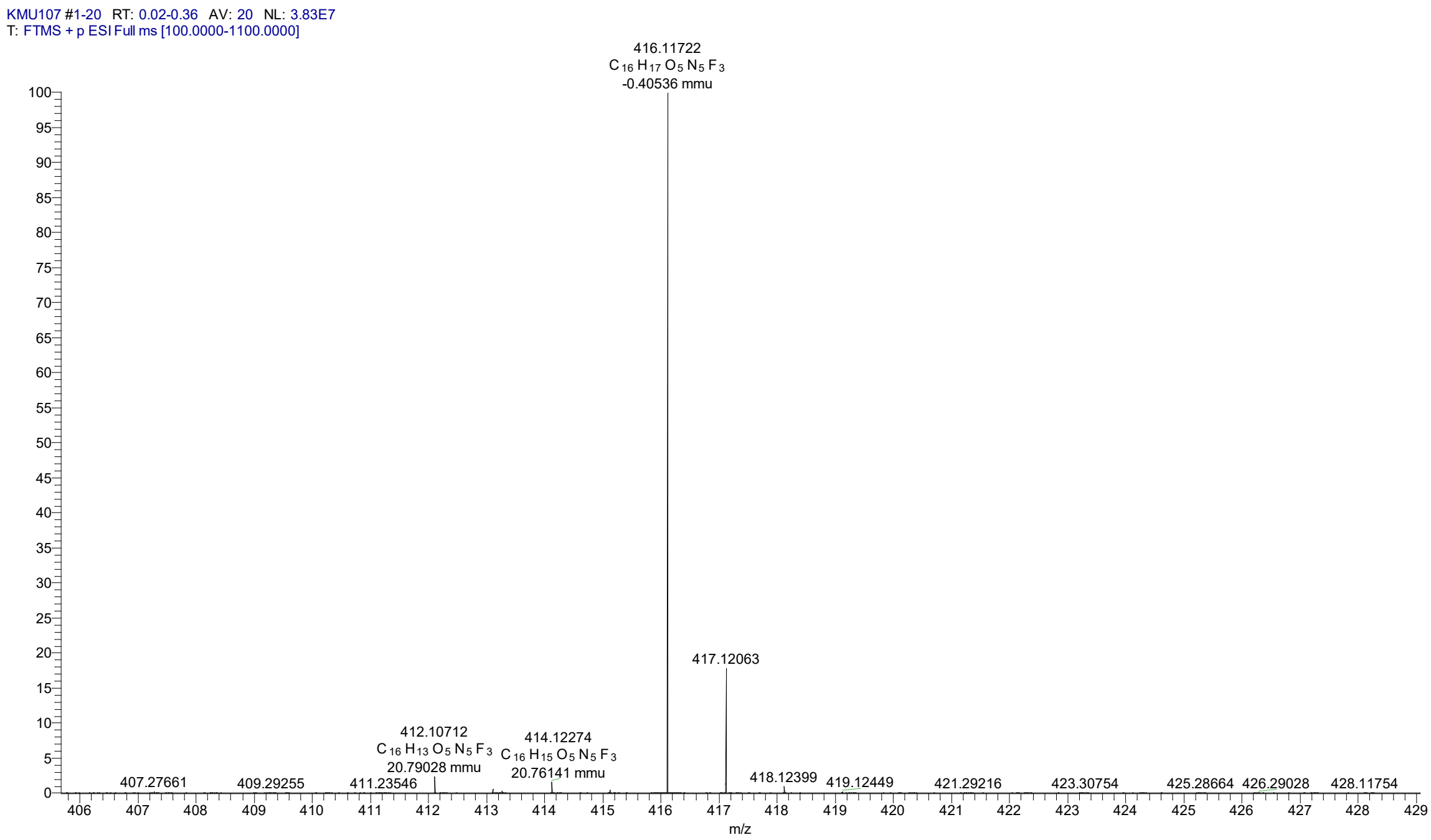

Figure S30. HR-MS analysis (ESI) of **22**.

Compound 18

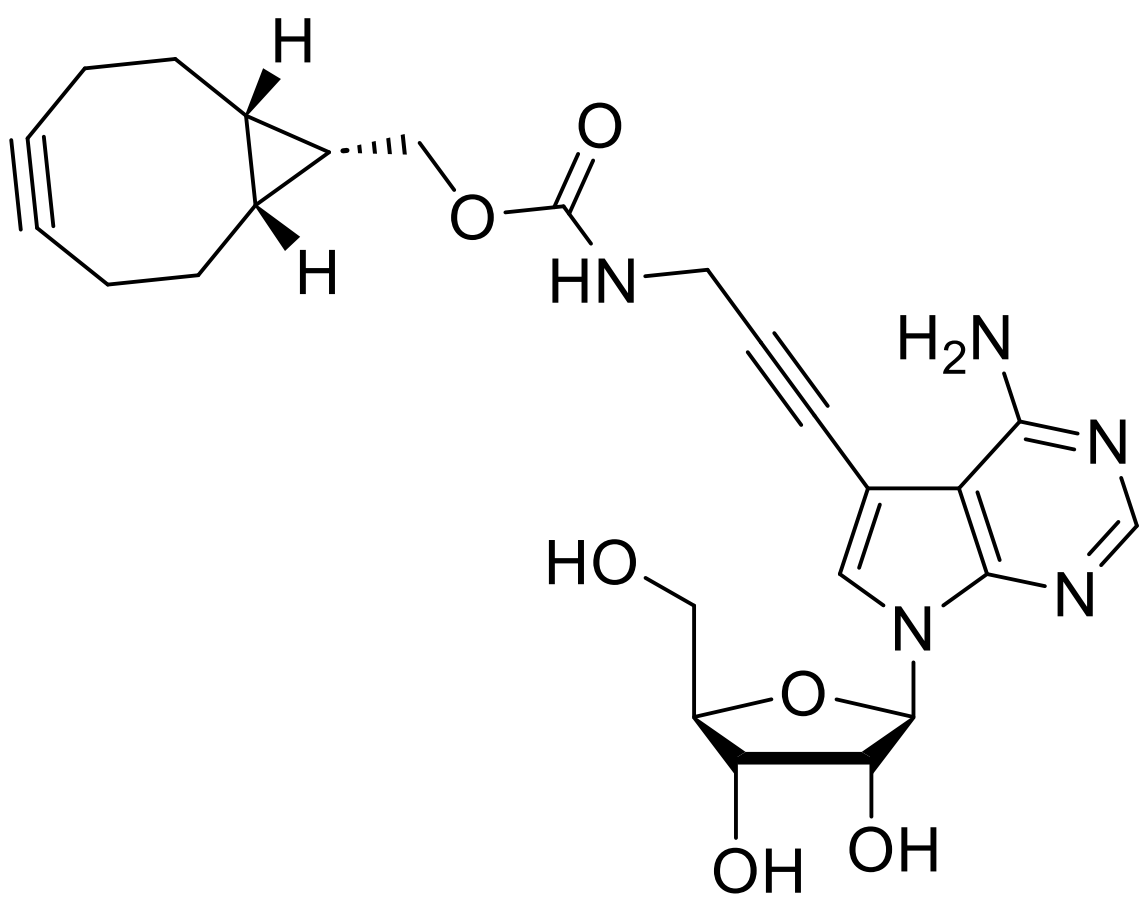

$C_{25}H_{29}N_5O_6$
495.54 g/mol

In a heated flask under argon atmosphere, 0.070 g **22** (0.25 mmol, 1.00 eq.) was dissolved in 3 mL of concentrate q. NH_4OH solution and stirred overnight at room temperature. The solvent was removed under reduced pressure and the residue was dried in a high vacuum. The product was obtained as a brown solid with a quantitative yield. In a heated round bottom flask under argon atmosphere, 0.046 g of the residue (0.14 mmol, 1.00 eq.), was dissolved in 5 mL dry DMF. Then 0.08 mL triethylamine (0.058 g, 0.58 mmol, 4.00 eq.) and 0.063 g BCN-NHS-ester (0.22 mmol, 1.50 eq.) were added, and the reaction mixture was stirred for 16 h at room temperature. The solvent was then removed under reduced pressure. The residue was dissolved in 25 mL of methanol, approx. 50 mg of Amberlite IRA 402 bicarbonate was added and stirred for 30 min. The solid was filtered off and the solvent was removed under reduced pressure. The crude

product was purified by column chromatography (SiO_2, CH_2Cl_2:MeOH 0 – 15 %). The product was obtained as a colorless solid in a yield of 0.056 g (0.11 mmol, 80 %).

^{1}H-NMR (400 MHz, DMSO-d_6): δ = 8.10 (s, 1H, H-2), 7.71 (m, 2H, H-8, N*H*), 6.00 (d, *J* = 6.0 Hz, 1H, H-1'), 5.32 (d, *J* = 6.3 Hz, 1H, 3'-OH), 5.20 (t, *J* = 5.5 Hz, 1H, 5'-OH), 5.11 (d, *J* = 4.8 Hz, 1H, 2'-OH), 4.35 (q, *J* = 6.0 Hz, 1H, H-3'), 4.10 (d, *J* = 8.1 Hz, 2H, BCN), 4.09 – 4.05 (m, 1H, H-4'), 4.03 (d, *J* = 5.5 Hz, 2H, C*H₂*), 3.89 (q, *J* = 3.4 Hz, 1H, H-2'), 3.62 (dt, *J* = 11.7, 4.5 Hz, 1H, H-5_a'), 3.54 (dt, *J* = 5.7, 2.8 Hz, 1H, H-5_b')., 2.27 – 2.08 (m, 6H, BCN), 1.55 (q, *J* = 10.4, 9.5 Hz, 2H, BCN), 1.35 – 1.26 (m, 1H, BCN), 0.91 – 0.83 (m, 2H, BCN).

^{13}C-NMR (126 MHz, DMSO-d_6): δ (ppm) = 157.6, 152.7, 149.6, 126.3, 102.4, 99.0, 94.5, 89.3, 87.2, 85.2, 75.2, 74.0, 70.5, 61.5, 30.9, 28.6, 20.8, 19.6, 17.6.

HR-MS (ESI): m/z (calculated for $C_{25}H_{30}N_5O_6^+$): 496.2191 [M+H]$^+$; found: 496.2186 [M+H]$^+$.

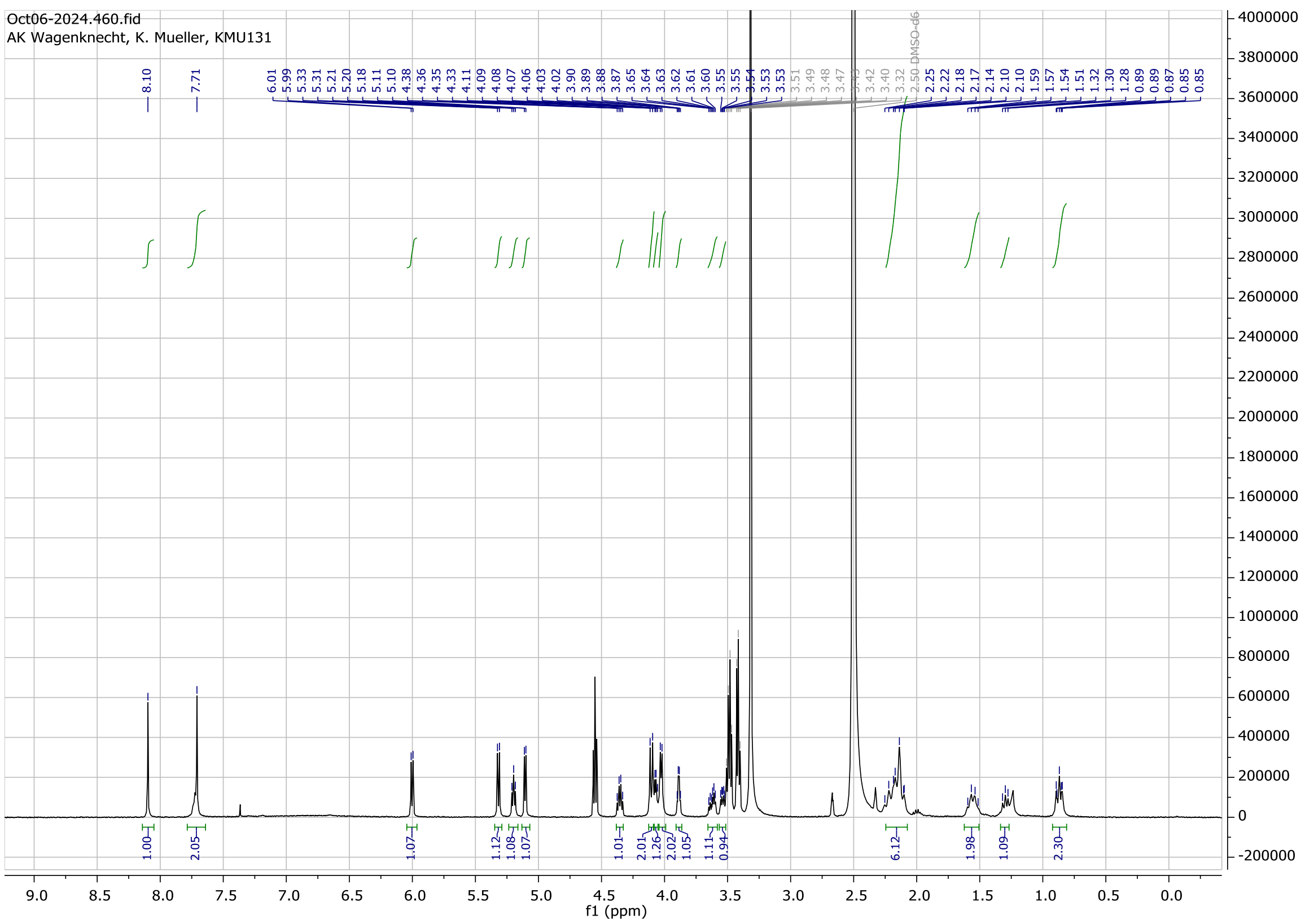

Figure S31. ^{1}H-NMR spectrum (400 MHz, DMSO-d_6) of **18**.

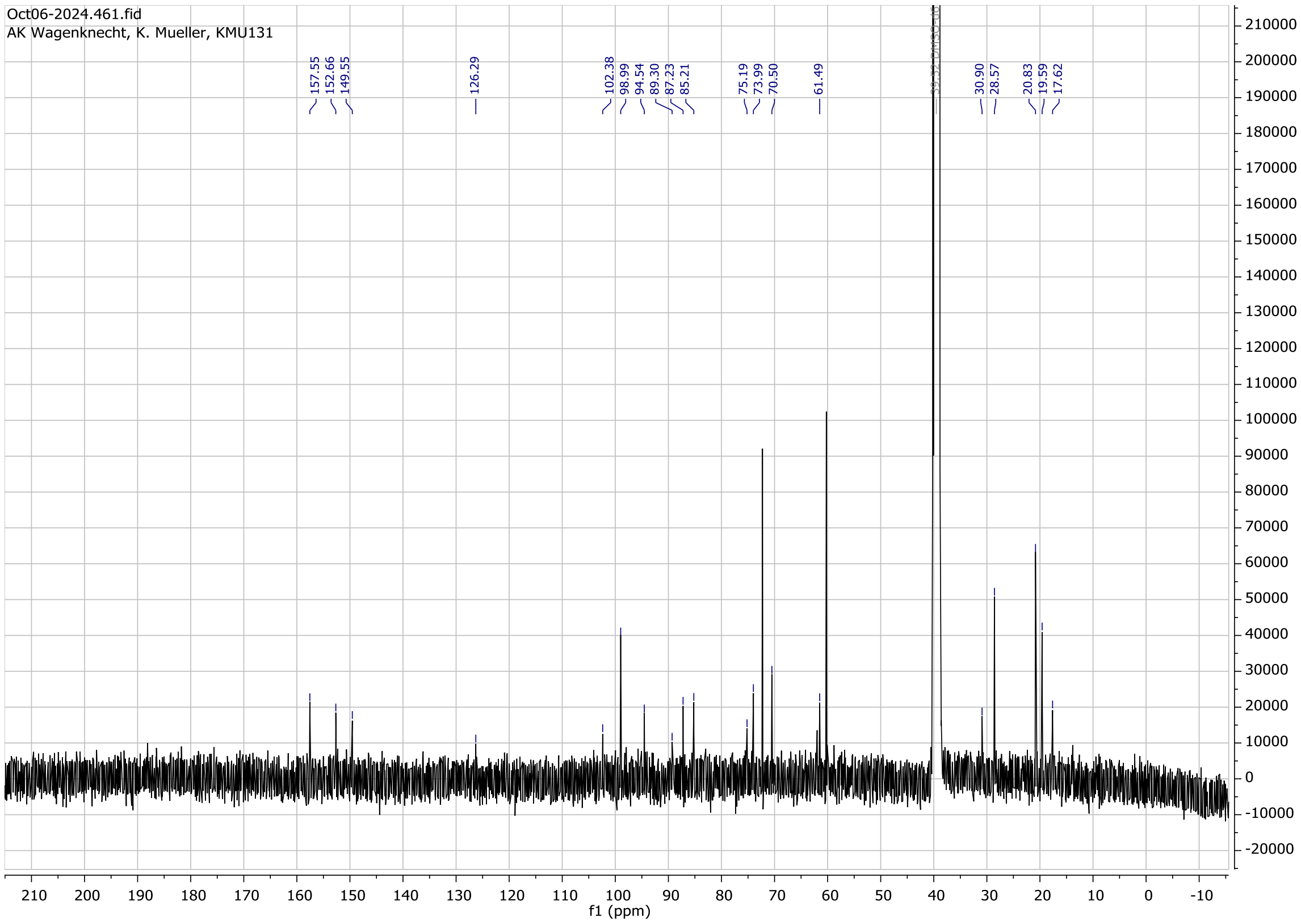

Figure S32. ^{13}C-NMR spectrum (126 MHz, DMSO-d_6) of **18**.

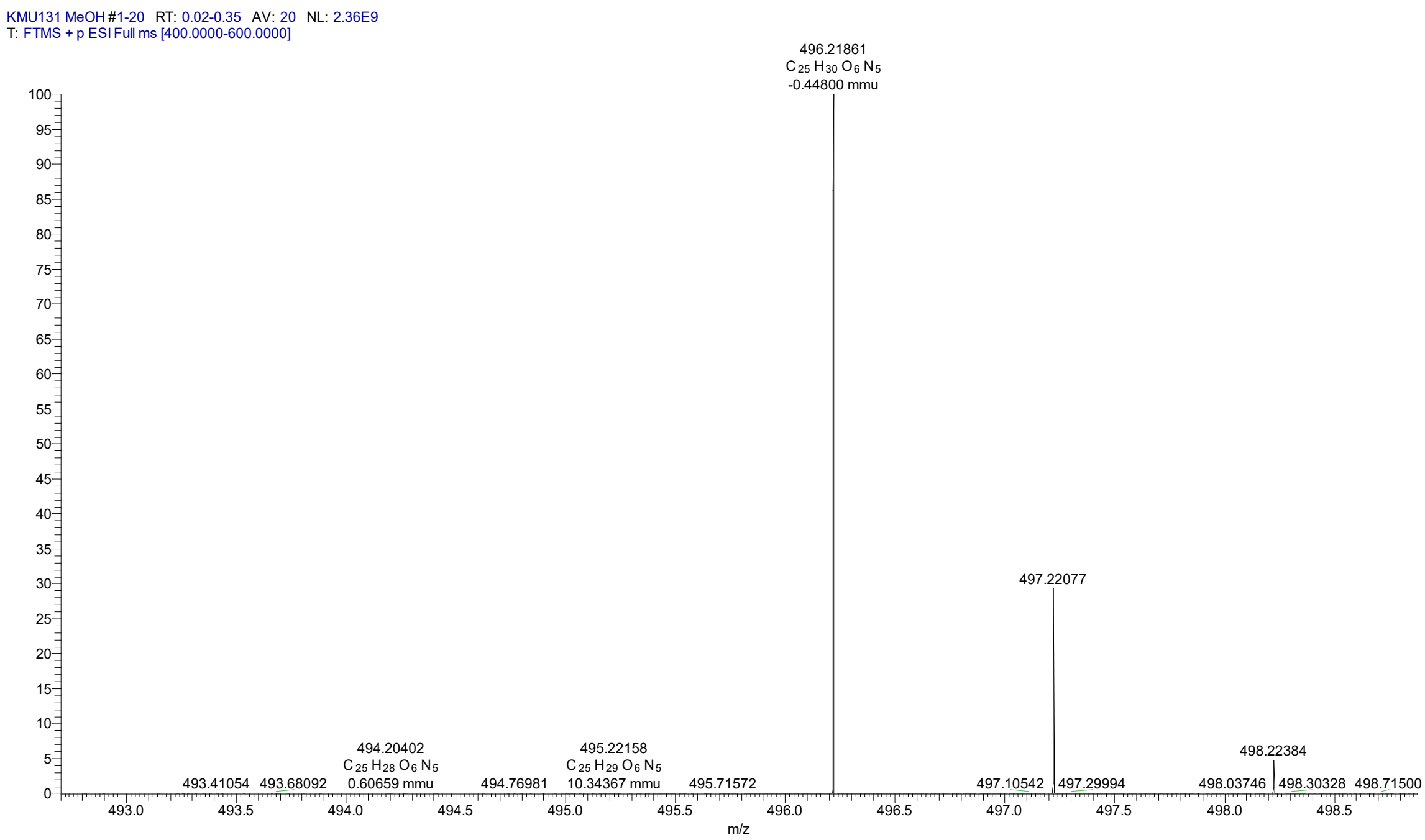

Figure S33. HR-MS analysis (ESI) of **18**.

3. DNA synthesis

DNA-BCN

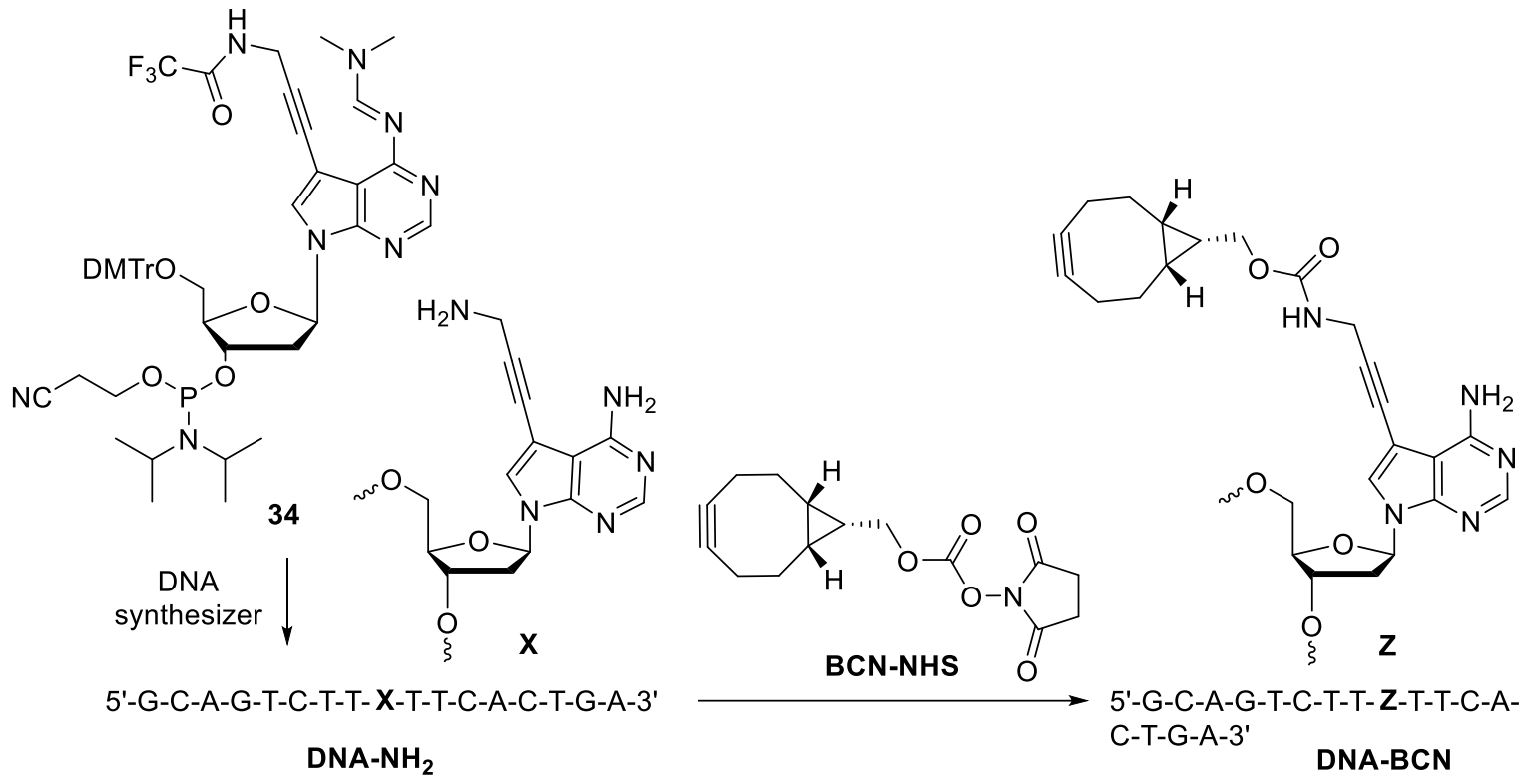

Figure S34. Synthesis of **DNA-BCN**.

The synthesis of the oligonucleotide precursor **DNA-NH₂** of **DNA-BCN** was performed using the K&A H-6 DNA/RNA synthesizer and was done in the 3' to 5' direction. *Controlled Pore Glass* (CPG) with an occupancy of 1 µmol (500 Å) was employed. The CPG columns as well as the reagents required for the synthesis were bought commercially from GLENRESEARCH, CHEMGENES, PROLIGO and SIGMA ALDRICH. The synthesis of **DNA-NH₂** was carried out under argon as an inert gas. The synthetic phosphoramitide **34**[31,34] was used as 0.1 M solution and the conventional phosphoramidites as 0.067 M solutions. A standard coupling protocol was used for the insertion of the conventional building blocks. For the synthetic building block **34**, the standard synthesis protocol was modified to achieve longer coupling times, which should ensure more effective insertion of the artificial building blocks. After the synthesis was completed, the CPG columns were dried under high vacuum for 1 h. The CPG column was opened and the column material transferred to a microcentrifuge tube. 700 µl aq. NH_4OH (> 25 %, *trace select*, FLUKA) was added to cleave **DNA-NH₂** from the solid phase and also to remove protective groups. The suspension was vortexed and was heated for 15 h at 55 °C in a heating block. After cooling the suspension, the ammonia was removed using a *CHRIST Alpha RVC* vacuum concentrator (35 min, 35 °C, 100 mbar). The supernatant solution was pipetted off and the residue was washed three times with 200 µl HPLC water The united centrifugates were lyophilized (25 °C, 0.1 mbar) after freezing in liquid nitrogen on a *CHRIST Alpha 1-2 LD Plus* lyophilization unit. **DNA-NH₂** was purified using DMT affinity columns from GLEN RESEARCH (Glen-pak™ DNA Purification Cartridges). The lyophilized crude **DNA-NH₂** was dissolved in 1 mL dd-H_2O. 1 mL NaCl-solution (100 mg/mL) was added to the solution to gain an end salt concentration of 50 mg/mL. The affinity column was first pretreated with 0.5 mL MeCN and 1 mL 2M Triethylammoniumacetat buffer (pH7). The oligonucleotide-salt solution was applied on to the column in 1 mL aliquots. After that, 1 mL of TFA solution (2 % in water) was added twice and the column was washed twice with 1 mL double distilled water. **DNA-NH₂** was eluted off the column with 1 ml 0.5 % NH_4OH in MeCN/water (1:1) and then evaporated to dryness under vacuum at 0.1 mbar and 25 °C overnight. Approximately 1 µmol of the oligonucleotide was dissolved in 300 µL dry DMSO. 2.00 mg BCN – NHS ester was dissolved in 300 µL dry DMSO and added to the oligonucleotide. The solution was then mixed with 5.00 µL Et_3N and shaken on a laboratory shaker at room temperature for 16 h. The solvent was then removed overnight under

vacuum at 0.1 mbar and 25 °C. A semi-preparative RP-HPLC was used to purify the postsynthetically modified **DNA-BCN**. The separation was carried out on a Dionex Ultimate3000 with autosampler, pump module, column oven, multi-diode array, RS fluorescence detector and fraction collector from THERMOFISHER SCIENTIFIC using Chromeleon 7 software. For semi-preparative separation, VDSphere OptiBio PUR 300 C18-SE column (250 x 10 mm, 5 µm) from VDS OPTILAB was used with a flow rate of 3.5 mL/min at a temperature of 40 °C. A gradient of 0 – 30 % MeCN and 0.1 M NH_4OAc over 30 minutes served as the mobile phase (0 min: 0% MeCN; 30 min: 20% MeCN; 40 min: 20% MeCN). **DNA-BCN** was dissolved in 300 µL dd water and was injected via the autosampler with an injection volume of 300 µL. The oligonucleotide was detected at a wavelength of 260 nm and 280 nm and collected in fractions. The purity of the collected fractions was verified by MALDI-TOF mass spectrometry. The identified pure fractions were combined and lyophilised. The concentration of **DNA-BCN** was calculated by using the *Lambert-Beer law*. For this purpose, the optical density at λ = 260 nm was measured on an *ND-1000 spectrophotometer* from NANODROP (in nucleic acid mode). The molar extinction coefficient ε_{260} of an oligonucleotide based only on natural bases at 260 nm in water is given by the following equation:

$$\varepsilon_{260} = (n_A \cdot \varepsilon_A + n_G \cdot \varepsilon_G + n_C \cdot \varepsilon_C + n_T \cdot \varepsilon_T) \cdot 0.9 + \varepsilon_{\mathbf{23}}$$

n_A, n_G, n_C, n_T: Number n of conventiobnal bases

$\varepsilon_{A,G,C,T}$: molar extinction coefficient 260 nm

ε_A=15,400 $M^{-1}cm^{-1}$; ε_G=11,700 $M^{-1}cm^{-1}$; ε_C=7,300 $M^{-1}cm^{-1}$; ε_T=8,800 $M^{-1}cm^{-1}$ ´; $\varepsilon_{\mathbf{23}}$=4,300 $M^{-1}cm^{-1}$[31]

0.9: correction factor for hypochromicity

DNA-BCN MS (MALDI-TOF): calcd: m/z=5386 $[M]^+$, exp: m/z=5389 $[M]^+$.

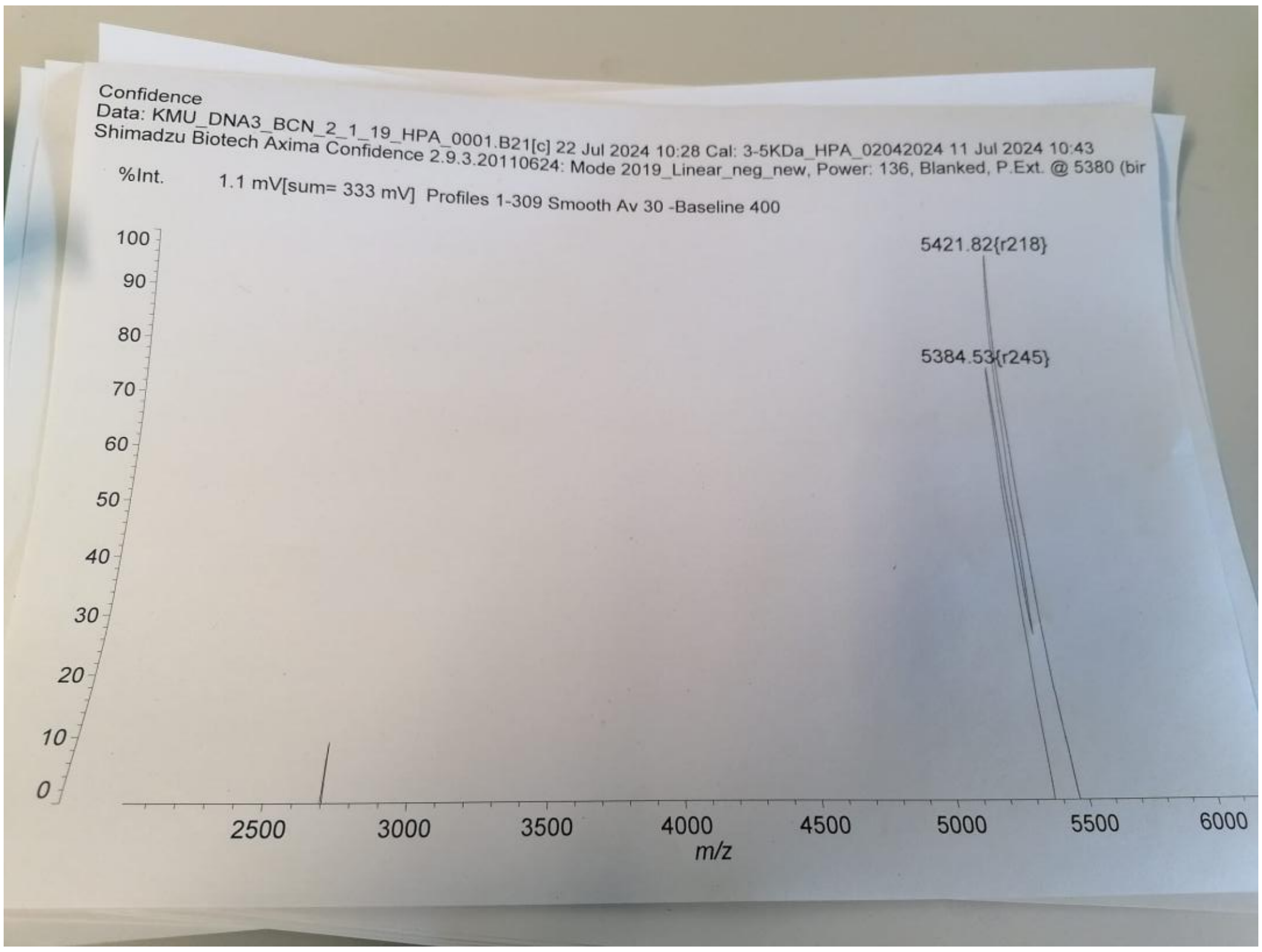

Figure S36. MS analysis (MALDI-TOF) of **DNA-BCN**.

4. SPSAC *in vitro*

SPSAC of 1-5 with 18 and 23

Quartz cuvettes with a light path of 10 mm and a probe volume of 0.50 mL were used. All reactants were prepared as stock solutions with 4 mM concentration for dyes **1**-**5** and 20 mM for **18** and **23**. In each case, 2.5 µL were diluted with double-distilled water to a total volume of 0.5 mL, resulting in a solvent composition of H_2O/DMSO (99:1 v/v%), concentration of 20 µM for dyes **1**-**5** of 100 µM for **18** and **23**, a ratio between dye:**18** and dye:**23** of 1:10 (Table S1). To monitor the progression of the reaction, the absorption was measured time-dependently, representatively for **1** and **2** with **23** (Figures S37 and S38). The fluorescence spectra were measured on a Fluoromax-4 spectrometer of the company HORIBA-SCIENTIFIC with a Peltier-element for temperature control. The measurements were corrected for Raman scattering of the respective solvent. Fluorescence spectra were recorded time-dependently to monitor the increase in fluorescence intensity (Figures S39-S49). The excitation wavelengths were adjusted to the dyes **1**-**5** (Table S2). A blank sample with H_2O/DMSO 99:1 was used to correct the starting fluorescence in addition to the dye (I_0). To determine the reaction rate constant k_2 by the increase in fluorescence, fluorescence spectra obtained were corrected, normalized and then integrated over the wavelengths λ_{exc} + 15 nm to 800 nm. The time-dependent fluorescence intensities were fitted using a monoexponential function.

The rate constant k_2 can be calculated from the curvature k_{obs} and is given in $M^{-1}s^{-1}$.

$$y = a + be^{-k_{obs}x}$$

The turn-on was determined by forming ratios of the fluorescence intensity at the end of the reaction and at the start of the reaction.

$$\text{tov} = \frac{area(I)}{area(I_0)}$$

tov *Turn-on value*

I integrated fluorescence intensity after time t

I_0 integrated fluorescence intensity at t = 0

Products **24**-**33** were identified by MS analysis (ESI, Table S3, Figure S50-S60).

Table S1. Sample preparation for determining the rate constants k_2 of dyes **1**-**5** with BCN-modified **18** and **23**.

	c_{1-5} [µM]	V_{stock} **1-5** [µL]	c_{18} or c_{23} [µM]	V_{stock} **18** or **23** [µL]	V_{H2O} [µL]	V_{DMSO} [µL]
**Control**	0	0	100	2.5	495	2.5
**SPSAC**	20	2.5	100	2.5	495	0

Table S2. Excitation wavelengths λ_{exc} for fluorescence spectroscopy and extinction coefficients ε of **1**-**5** in DMSO.

Dye	λ_{exc} [nm]	E [M^{-1} cm^{-1}]
**1**	437	33.000
**2**	424	10.300
**3**	448	33.000
**4**	424	17.000
**5**	372	16.200

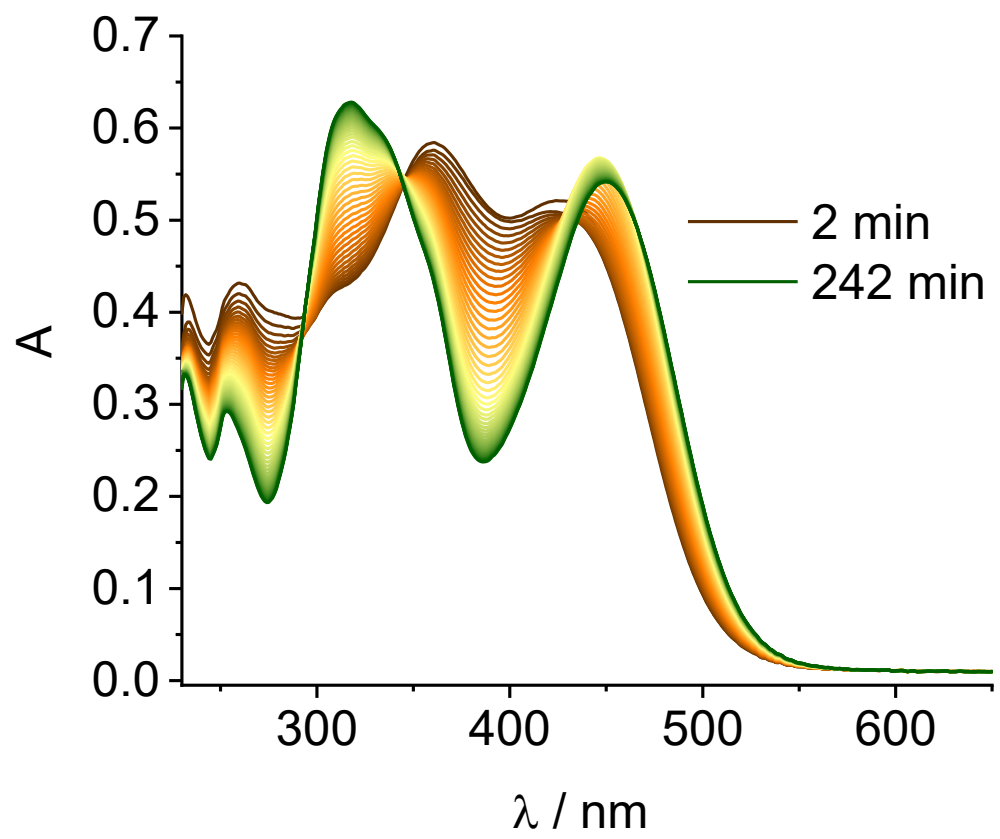

Figure S37. Time-dependent UV Vis absorption of SPSAC of **1** with **23**.

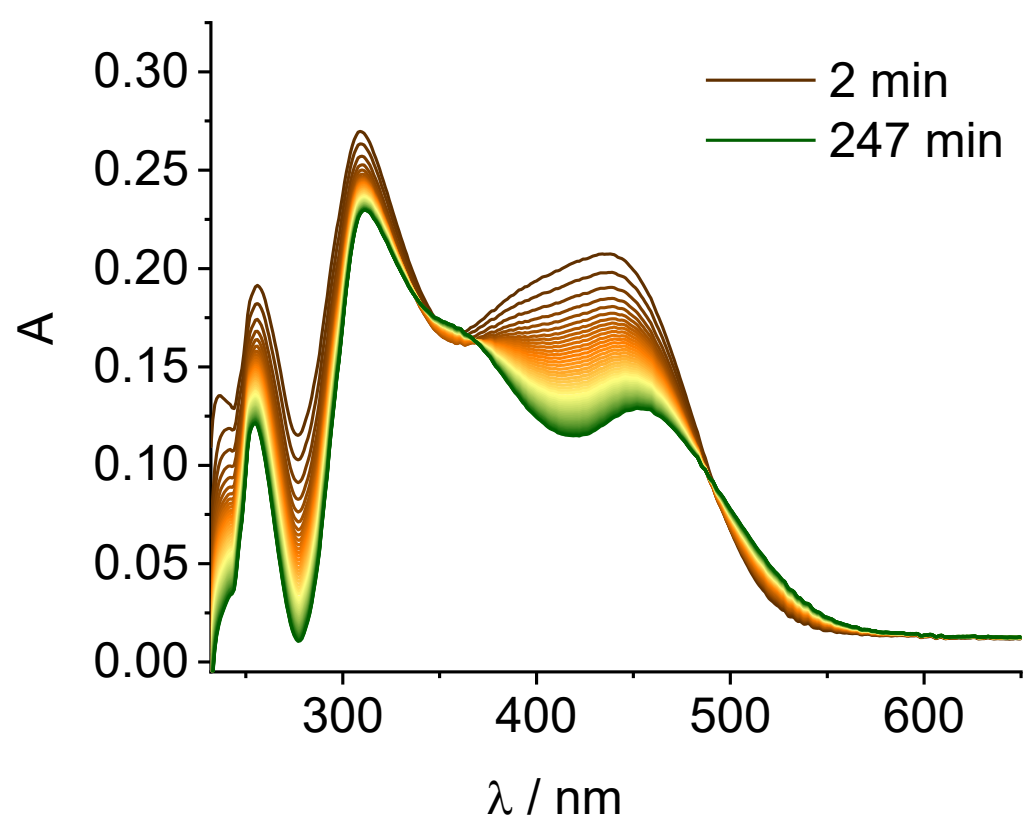

Figure S38. Time-dependent UV Vis absorption of SPSAC of **2** with **23**.

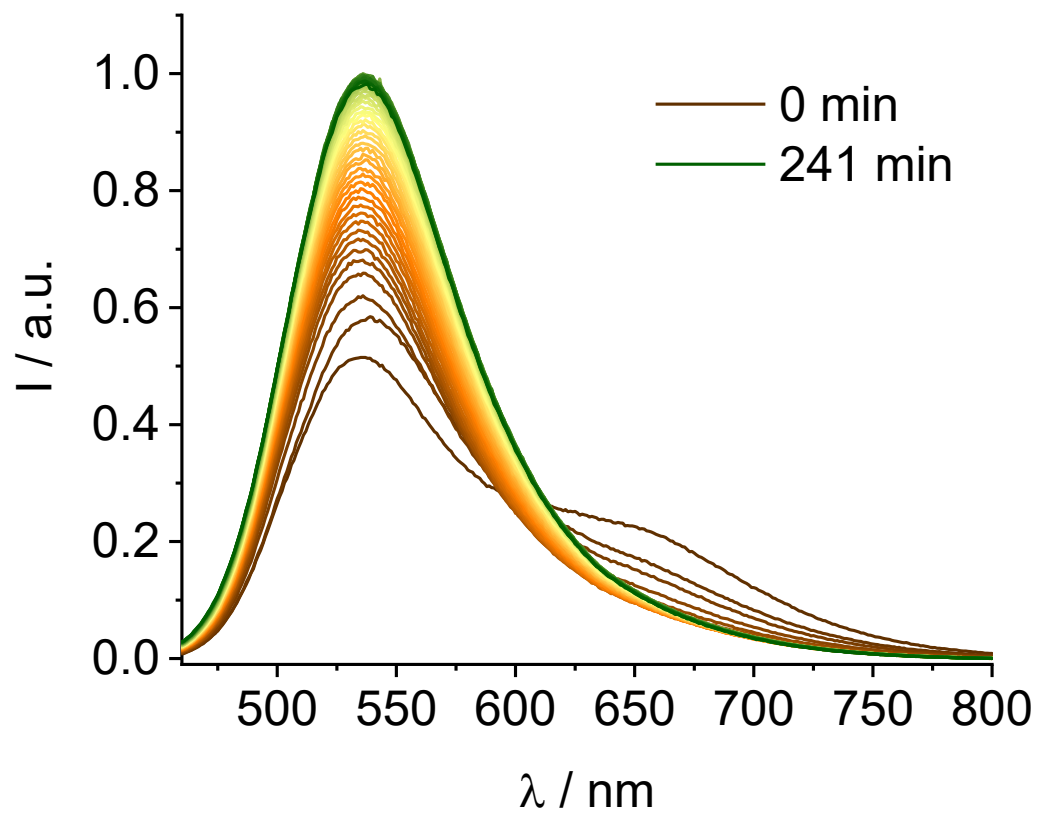

Figure S39. Time-dependent fluorescence of SPSAC of **1** with **18**.

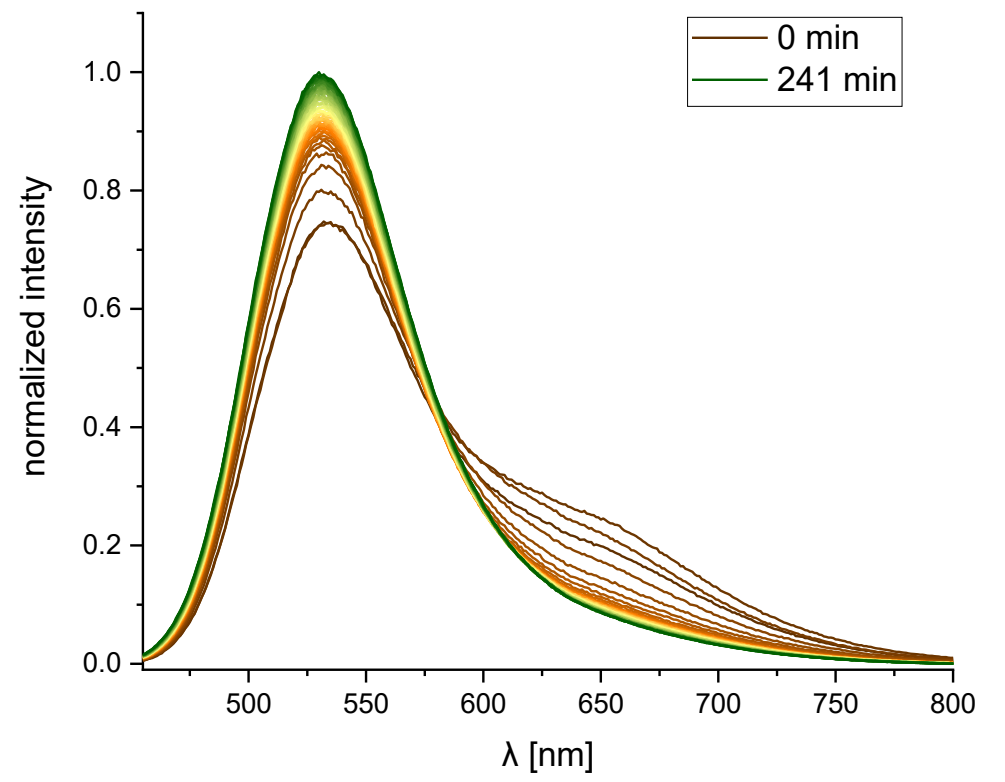

Figure S40. Time-dependent fluorescence of SPSAC of **1** with **23**.

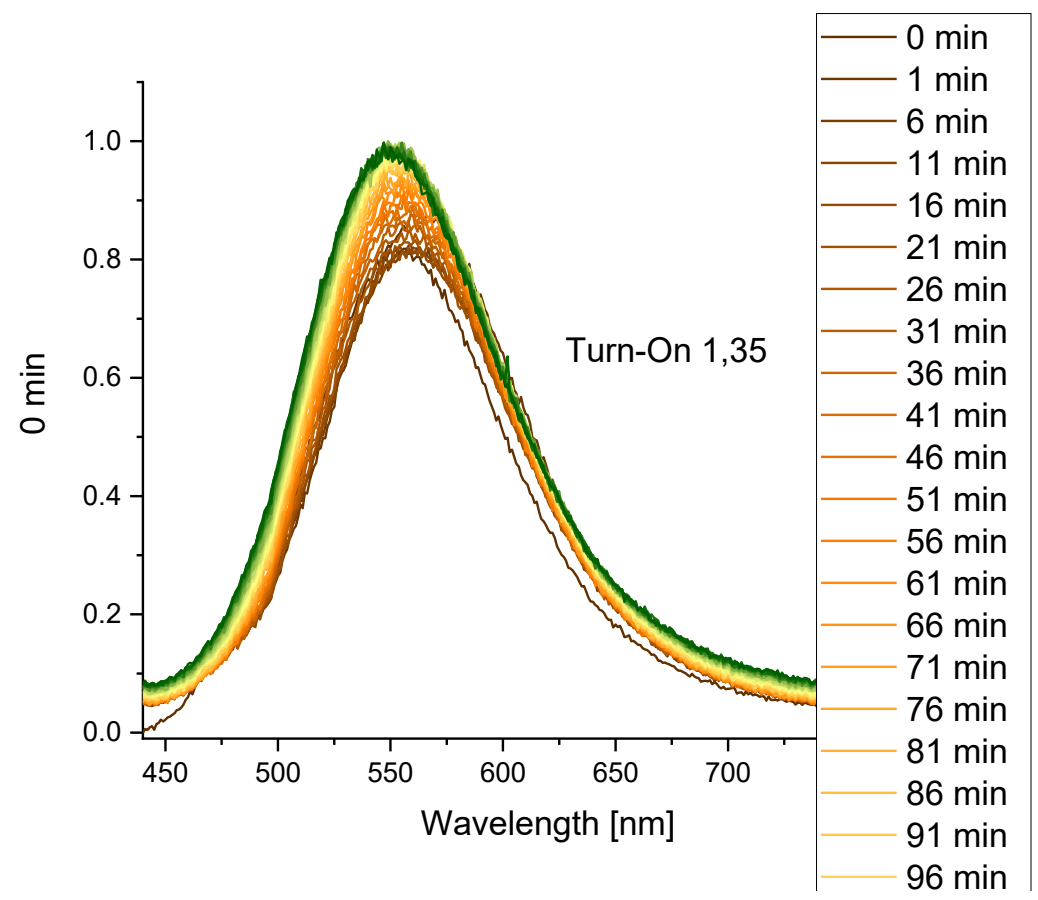

Figure S41. Time-dependent fluorescence of SPSAC of **2** with **18**.

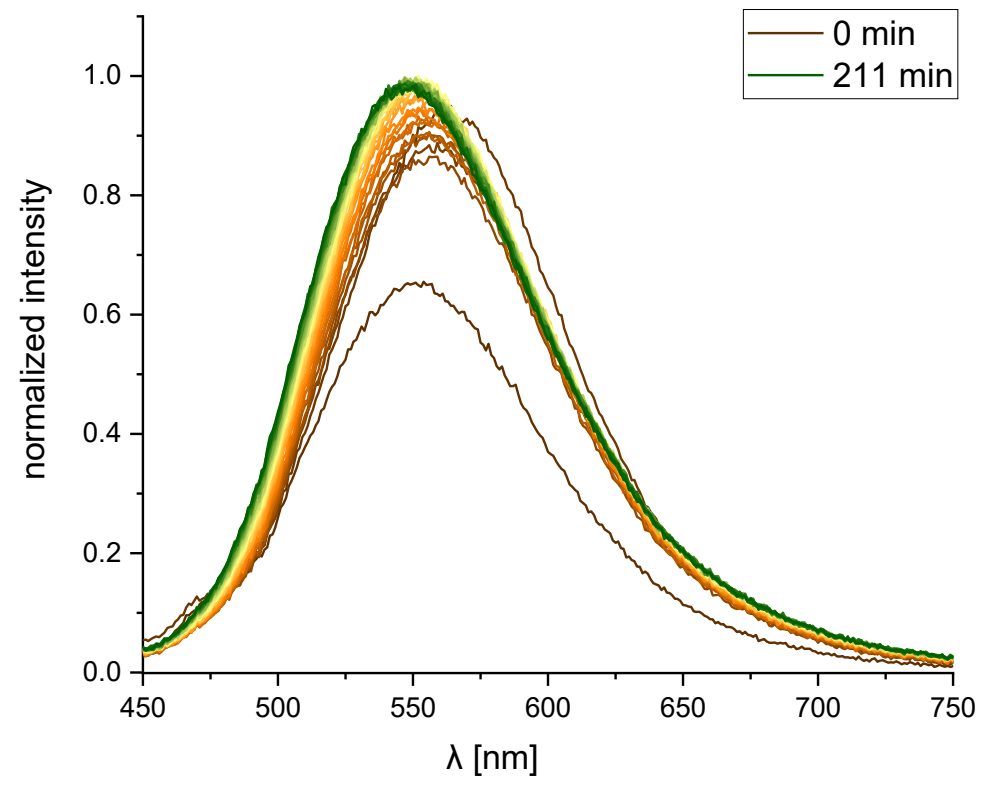

Figure S42. Time-dependent fluorescence of SPSAC of **2** with **23**.

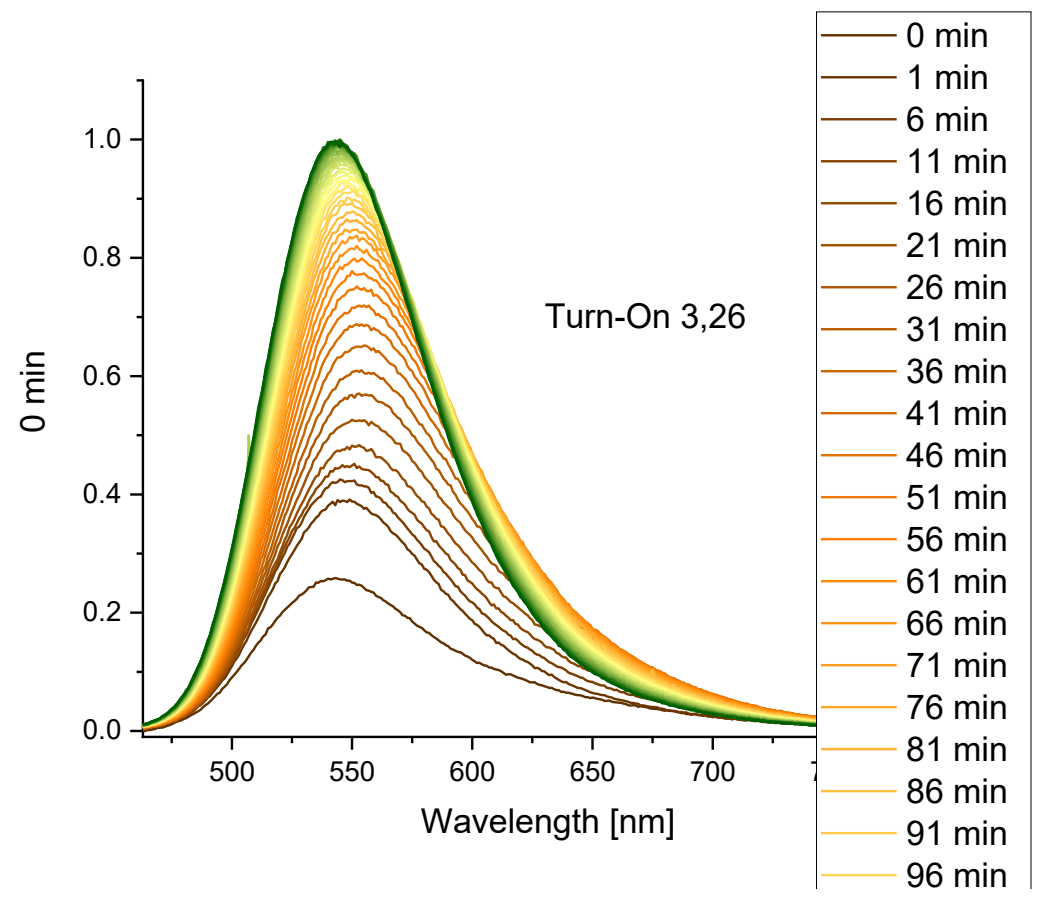

Figure S43. Time-dependent fluorescence of SPSAC of **3** with **18**.

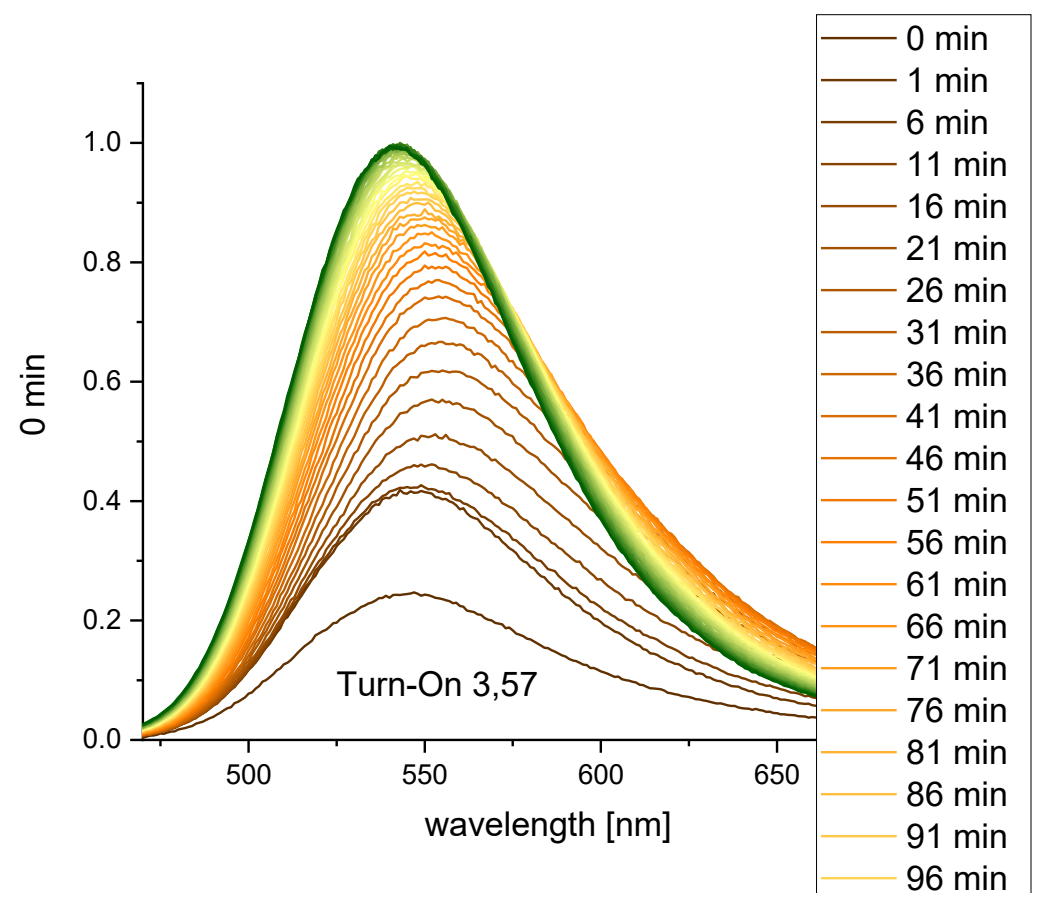

Figure S44. Time-dependent fluorescence of SPSAC of **3** with **23**.

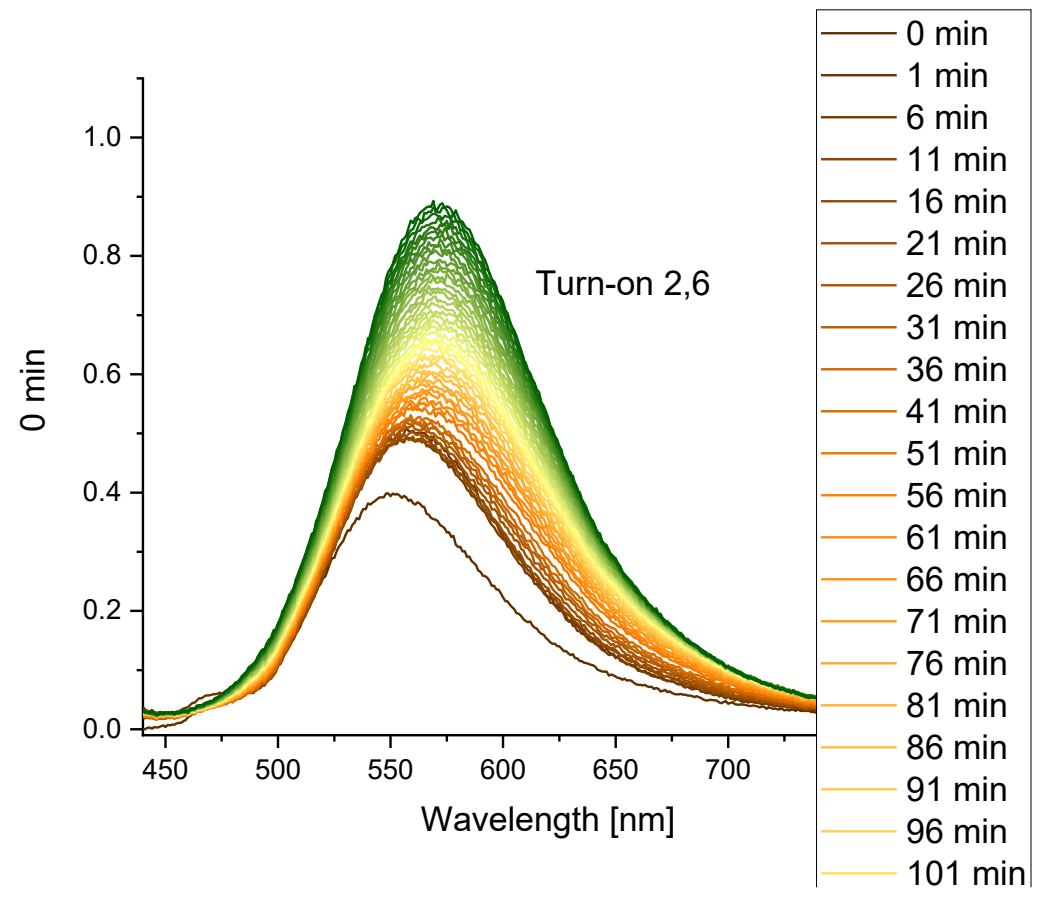

Figure S45. Time-dependent fluorescence of SPSAC of **4** with **18**.

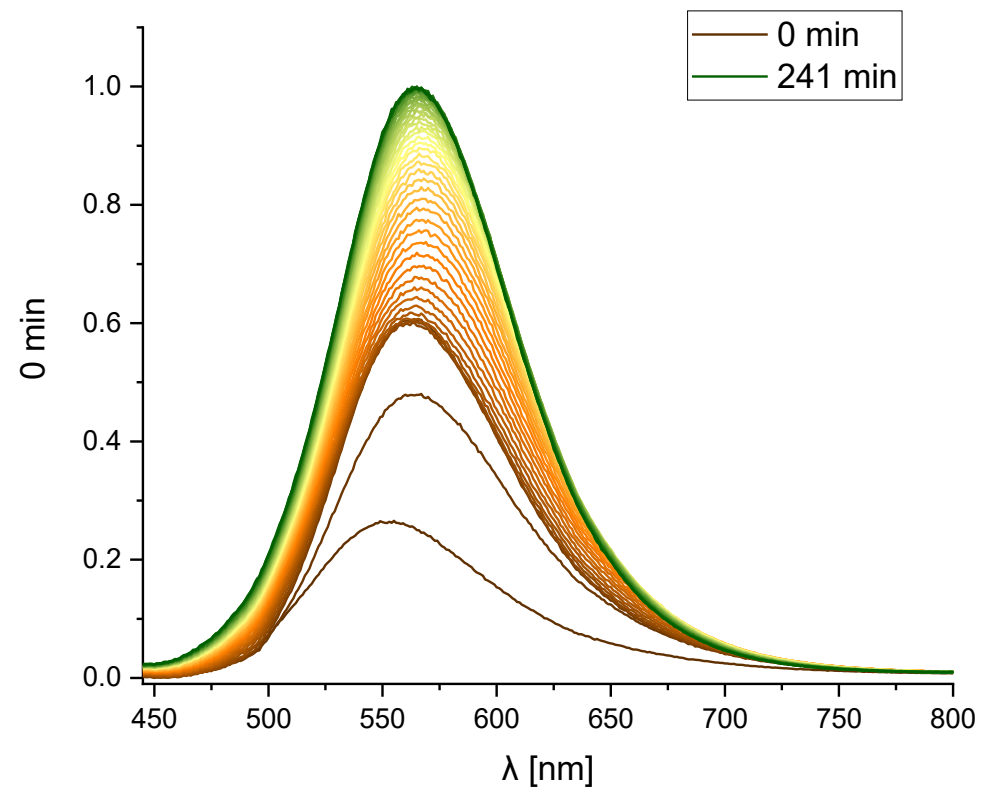

Figure S46. Time-dependent fluorescence of SPSAC of **4** with **23**.

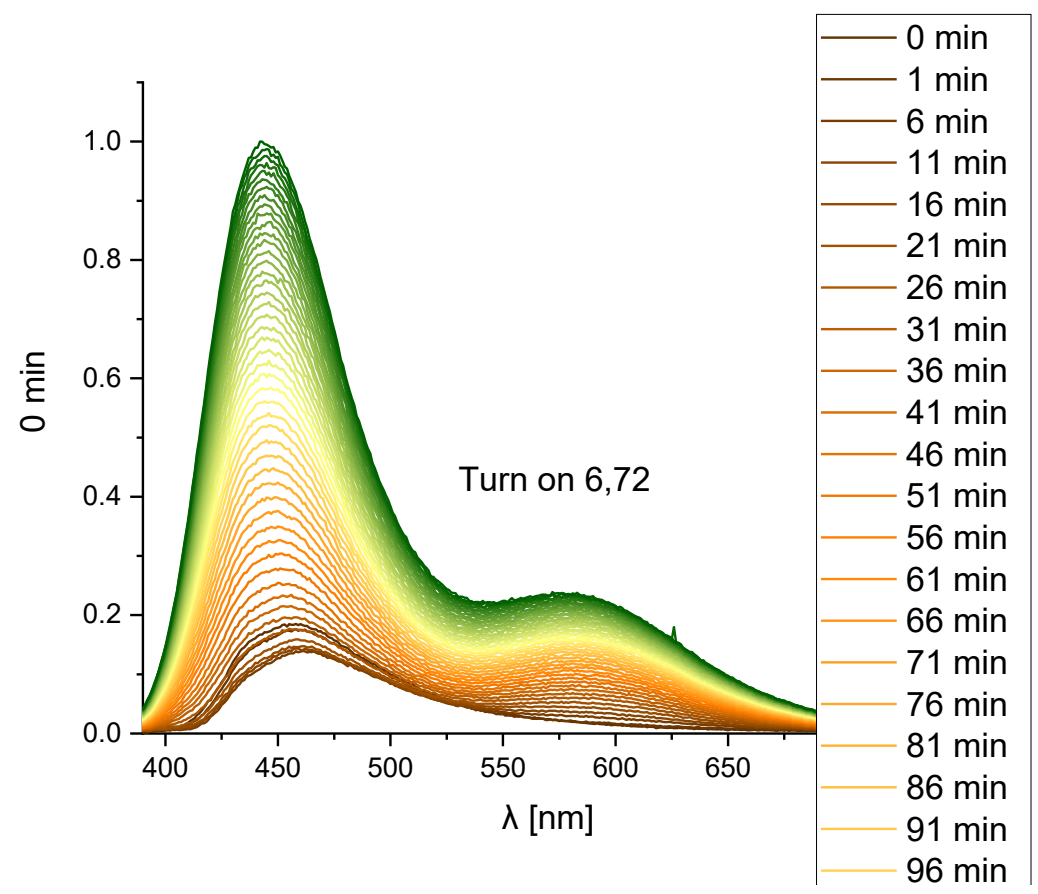

Figure S47. Time-dependent fluorescence of SPSAC of **5** with **18**.

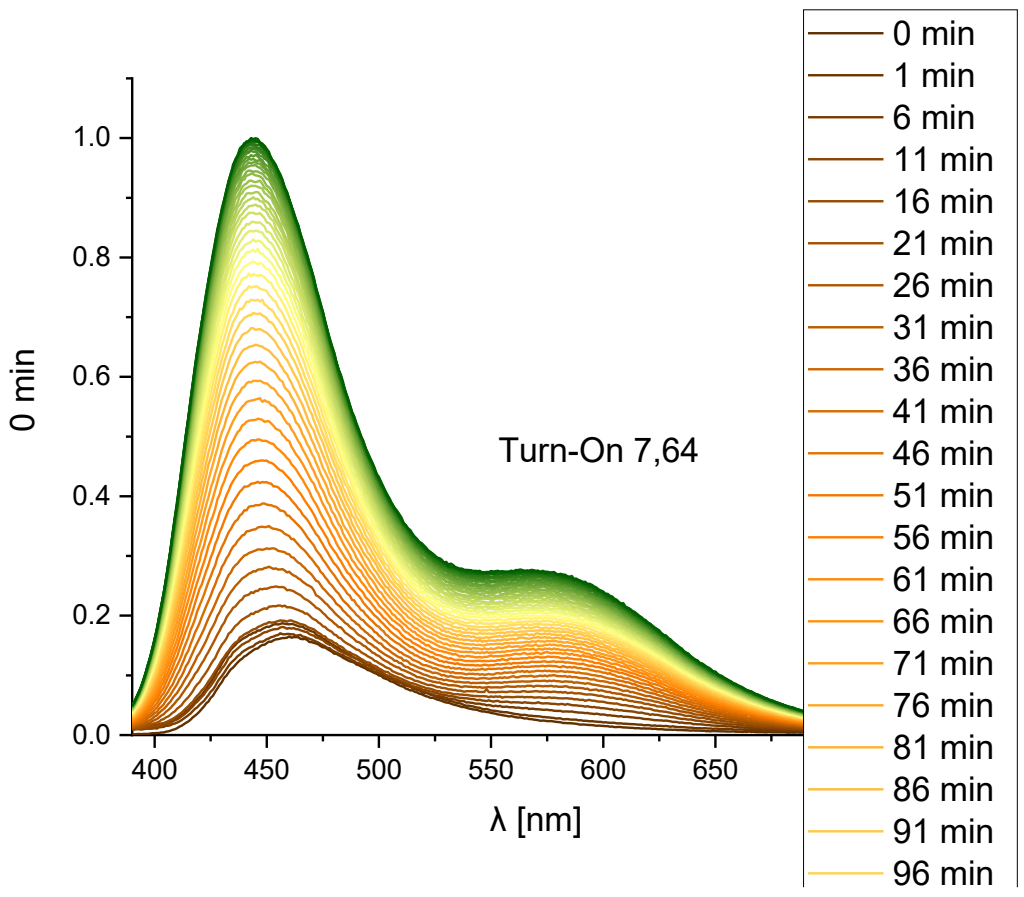

Figure S48. Time-dependent fluorescence of SPSAC of **5** with **23**.

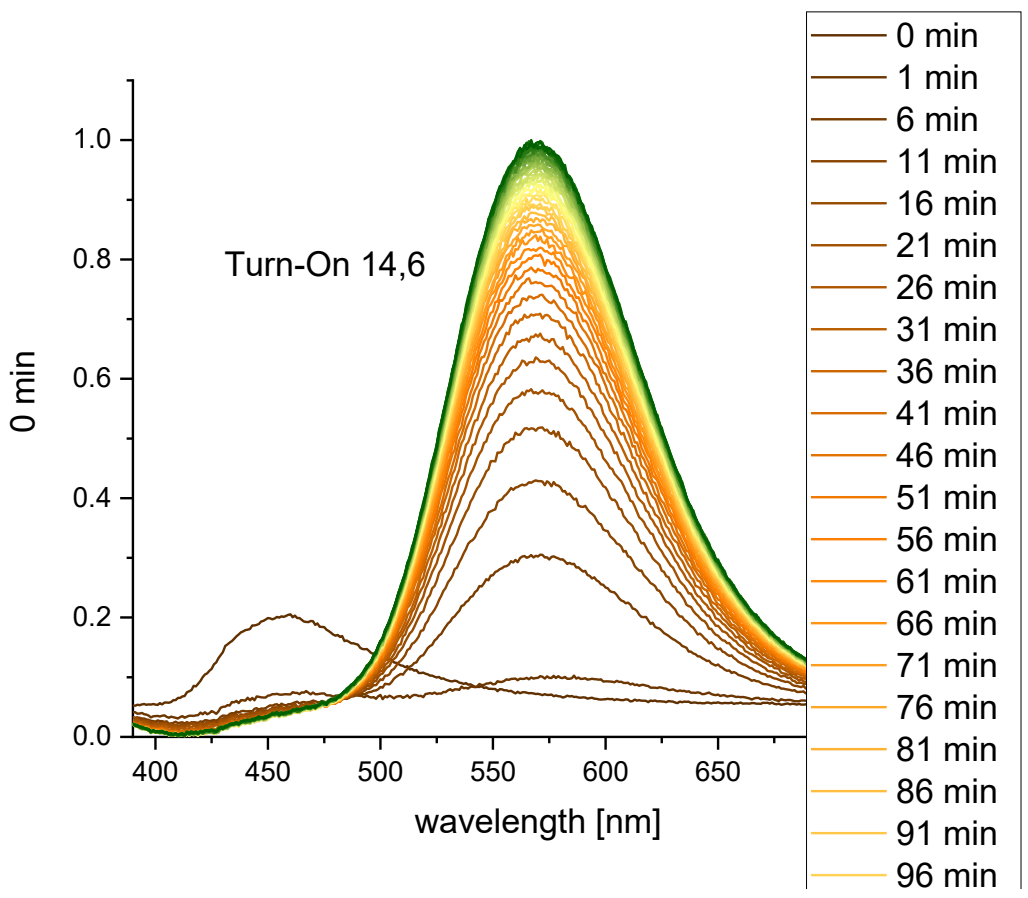

Figure S49. Time-dependent fluorescence of SPSAC of **5** with **DNA-BCN**.

Table S3. MS analysis (ESI) of products **24**-**33**.

product	calculated mass [m/z]	measured mass [m/z]
**24**	886.4035	886.4007
**25**	870.4086	870.4080
**26**	886.4035	886.4007
**27**	870.4086	870.4057
**28**	936.4192	936.4161
**29**	920.4242	920.4204
**30**	936.4192	936.4161
**31**	920.4242	920.4214
**32**	781.3457	781.3426
**33**	765.3507	765.3477

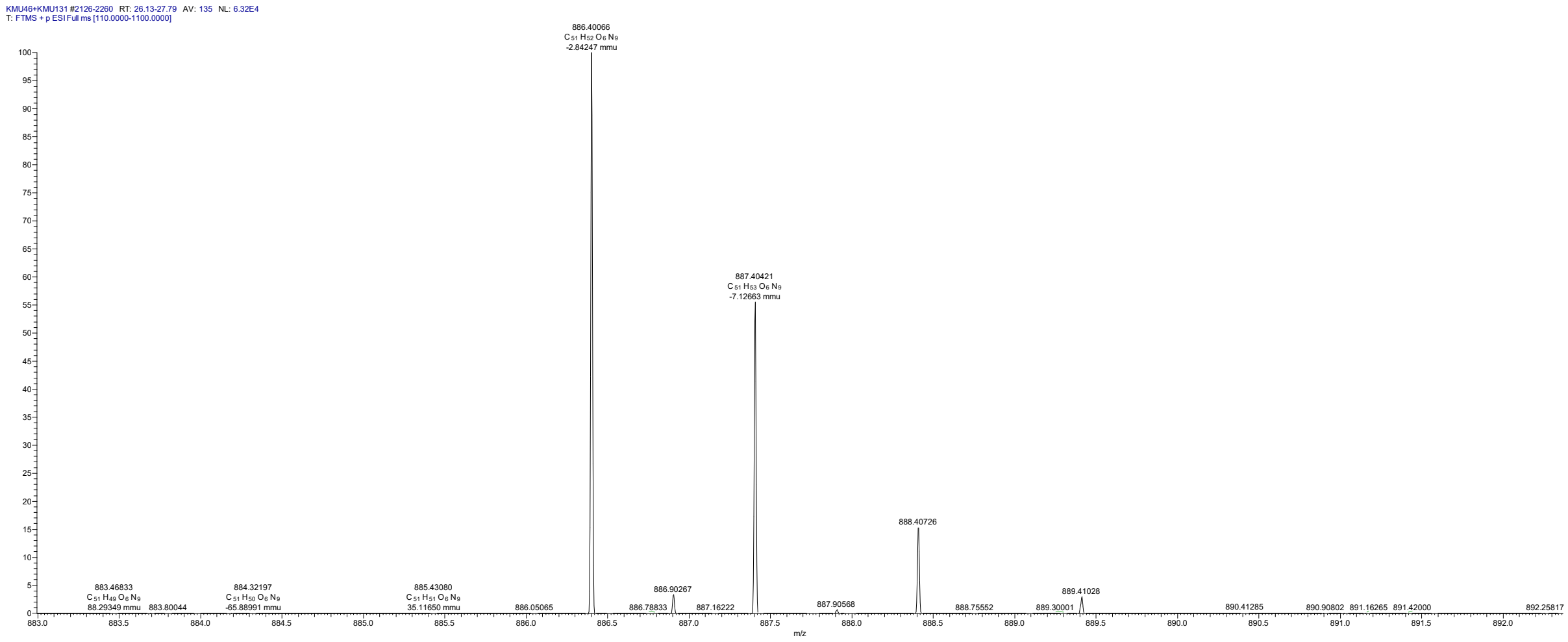

Figure S50. MS analysis (ESI) of product **24**.

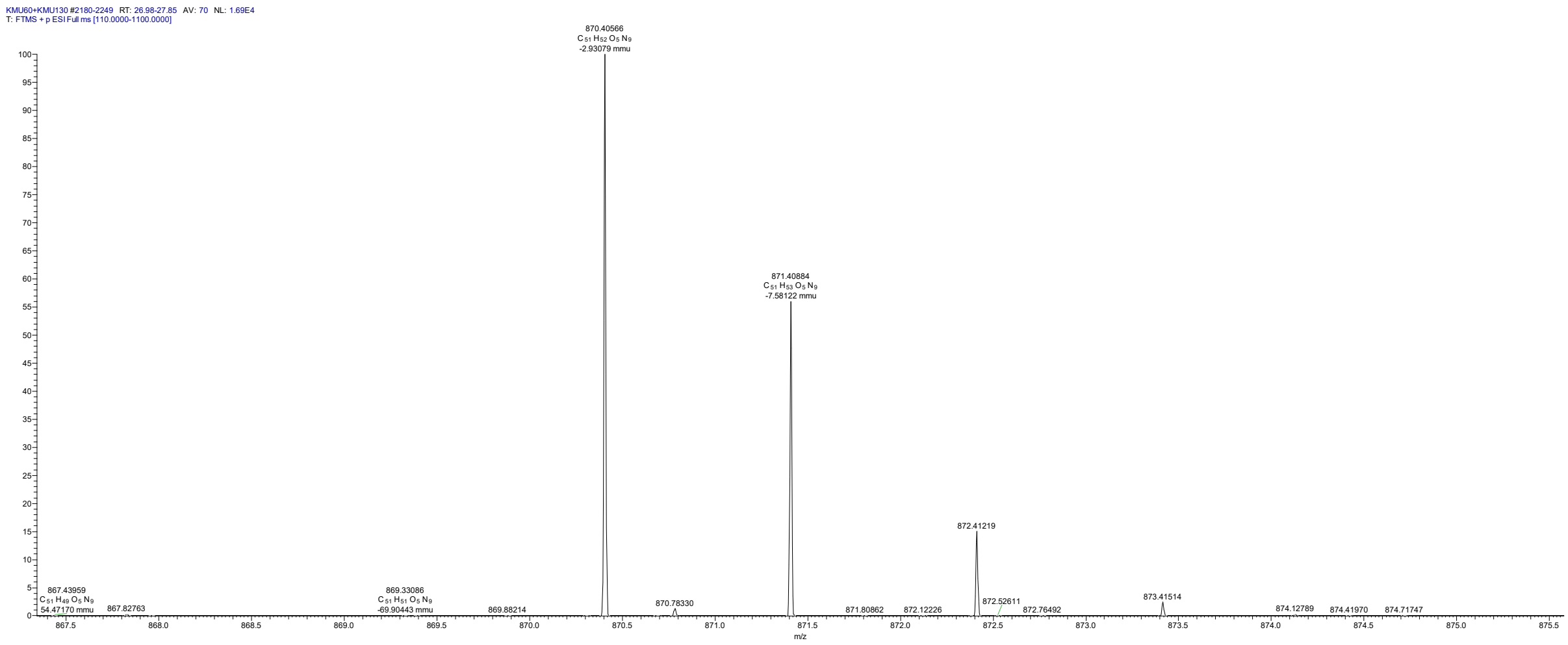

Figure S51. MS analysis (ESI) of product **25**.

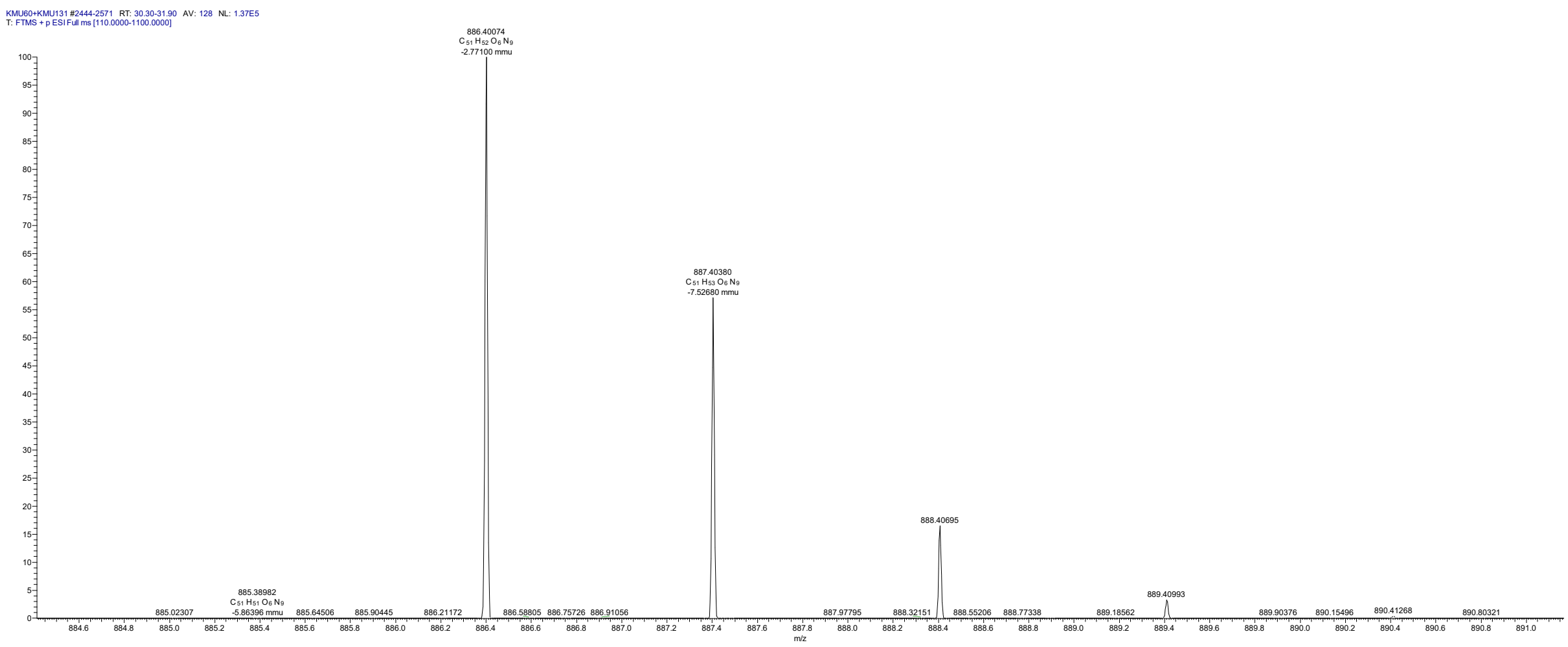

Figure S52. MS analysis (ESI) of product **26**.

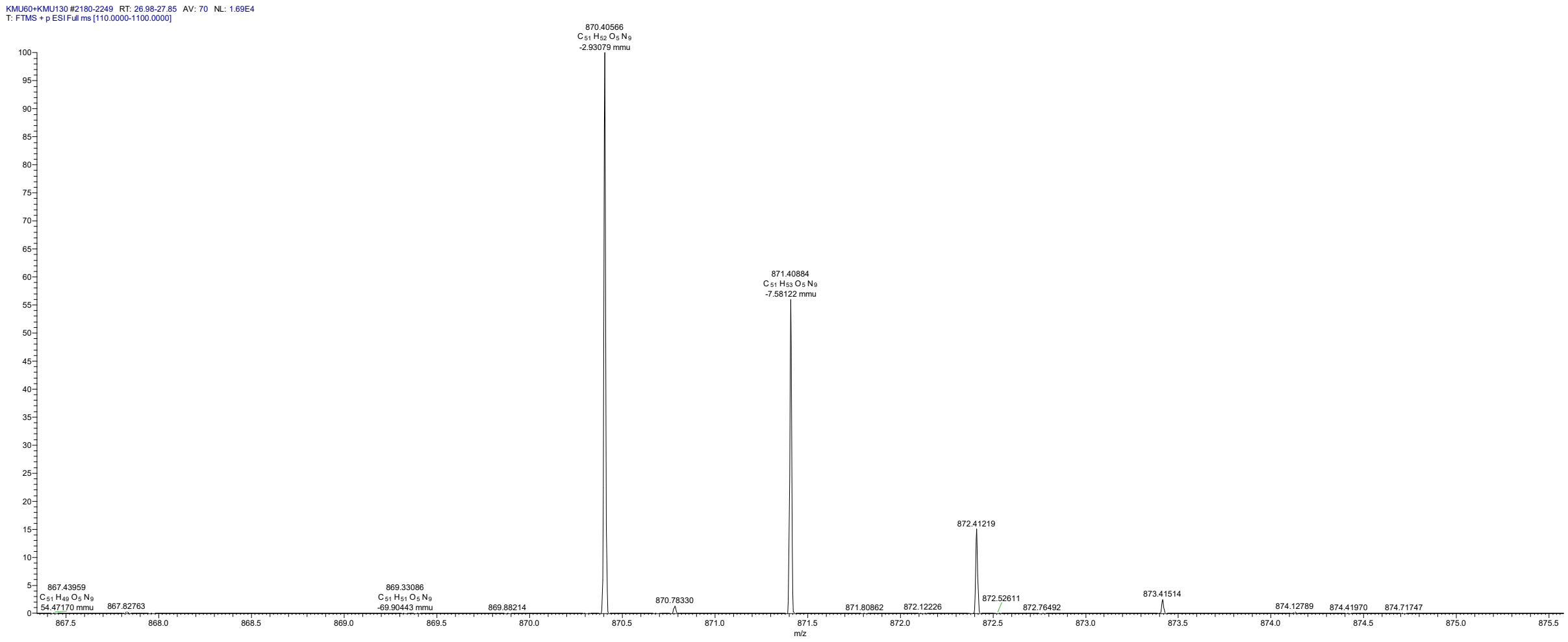

Figure S53. MS analysis (ESI) of product **27**.

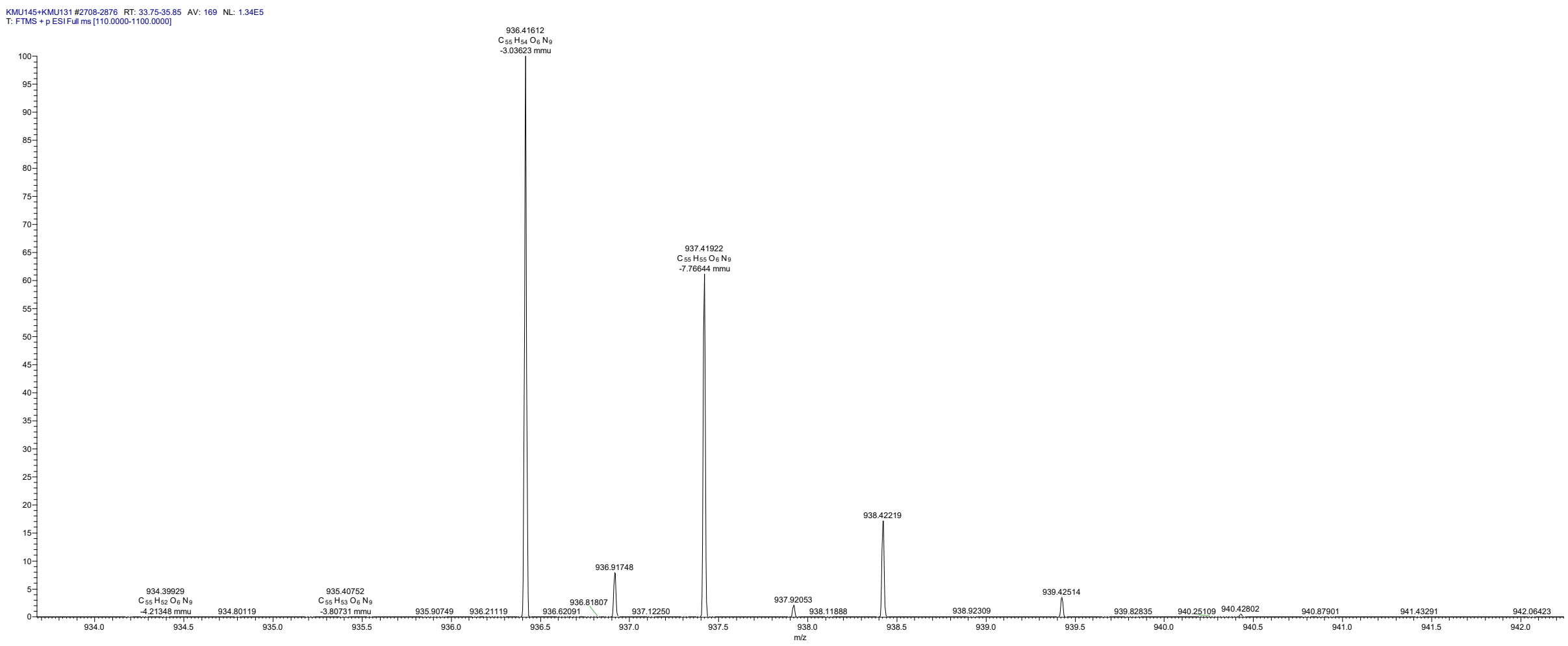

Figure S54. MS analysis (ESI) of product **28**.

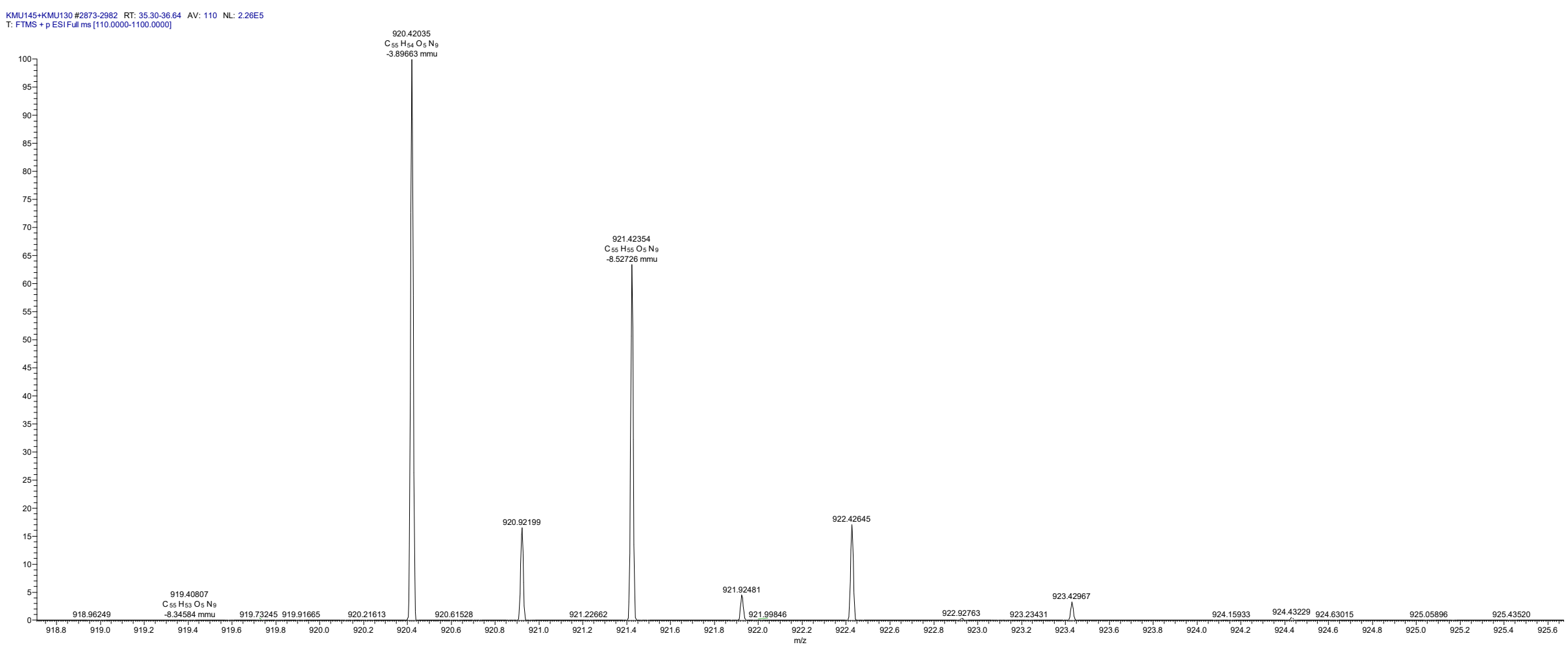

Figure S55. MS analysis (ESI) of product **29**.

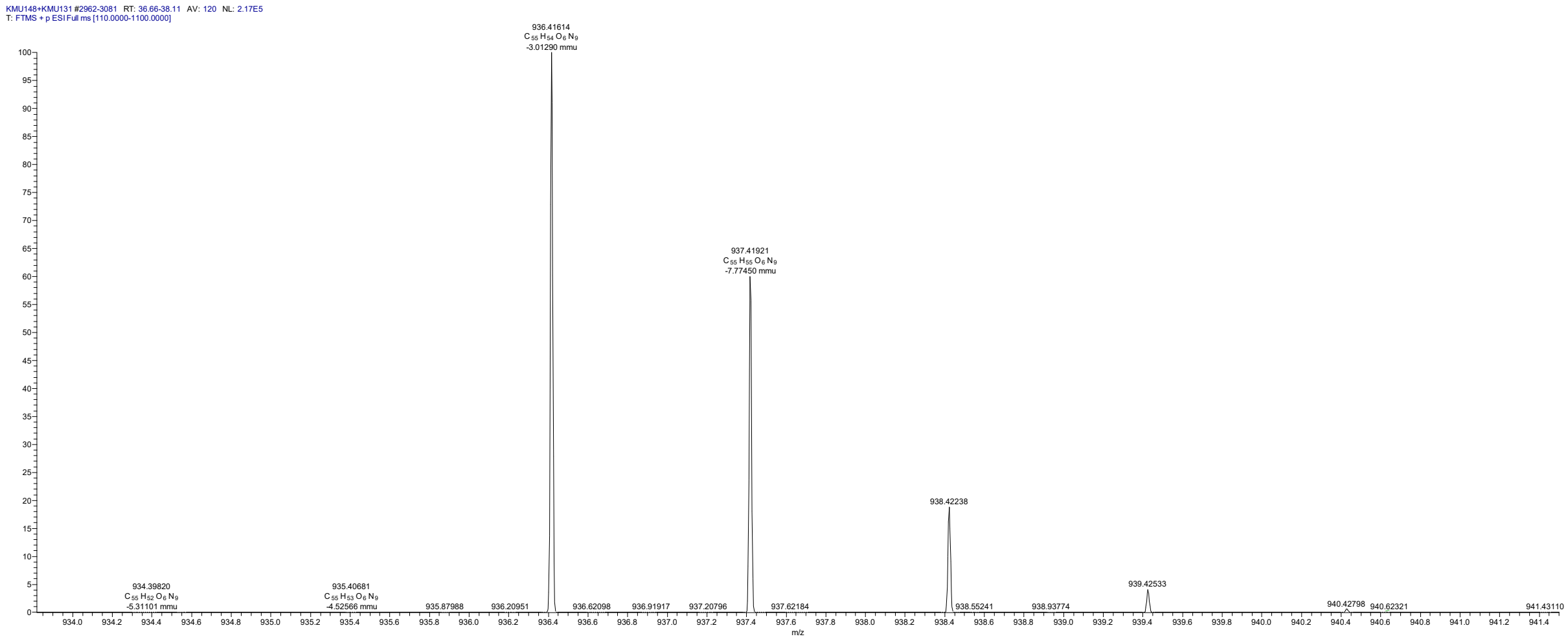

Figure S56. MS analysis (ESI) of product **30**.

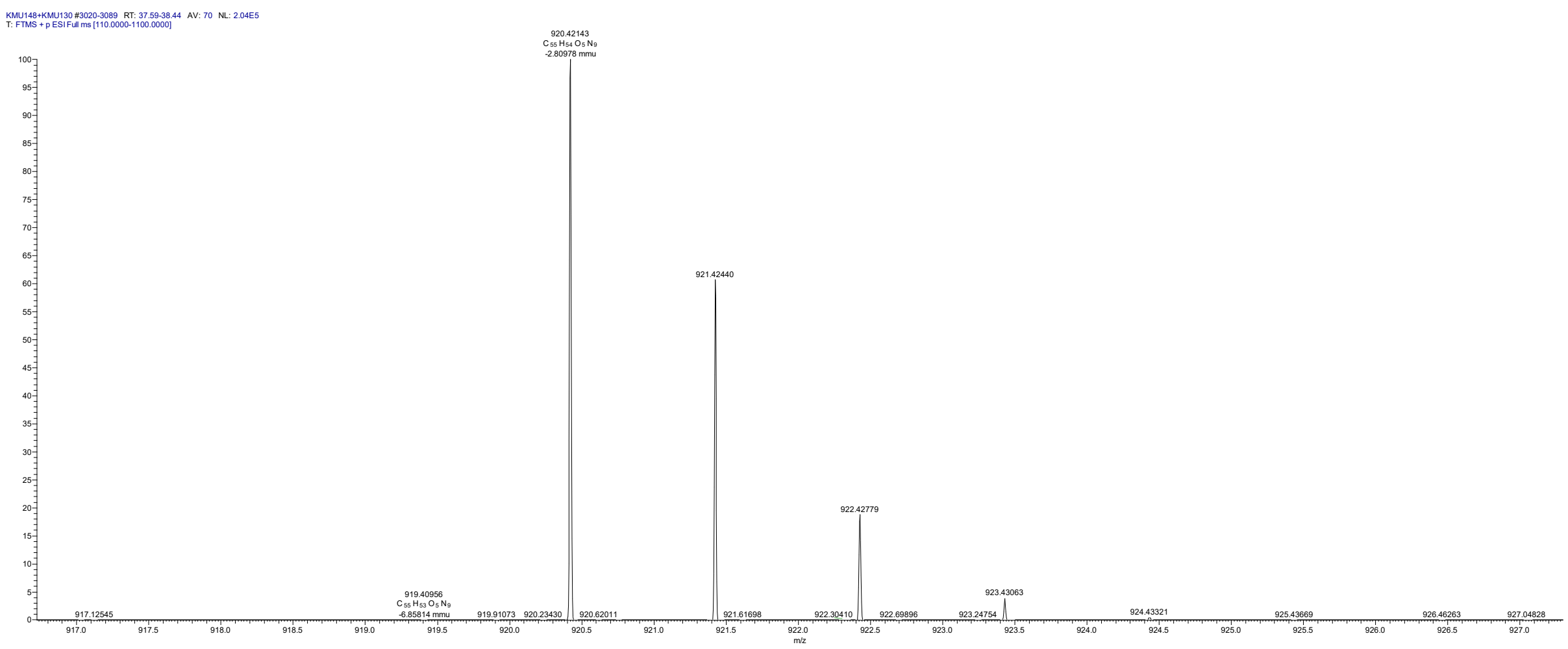

Figure S57. MS analysis (ESI) of product **31**.

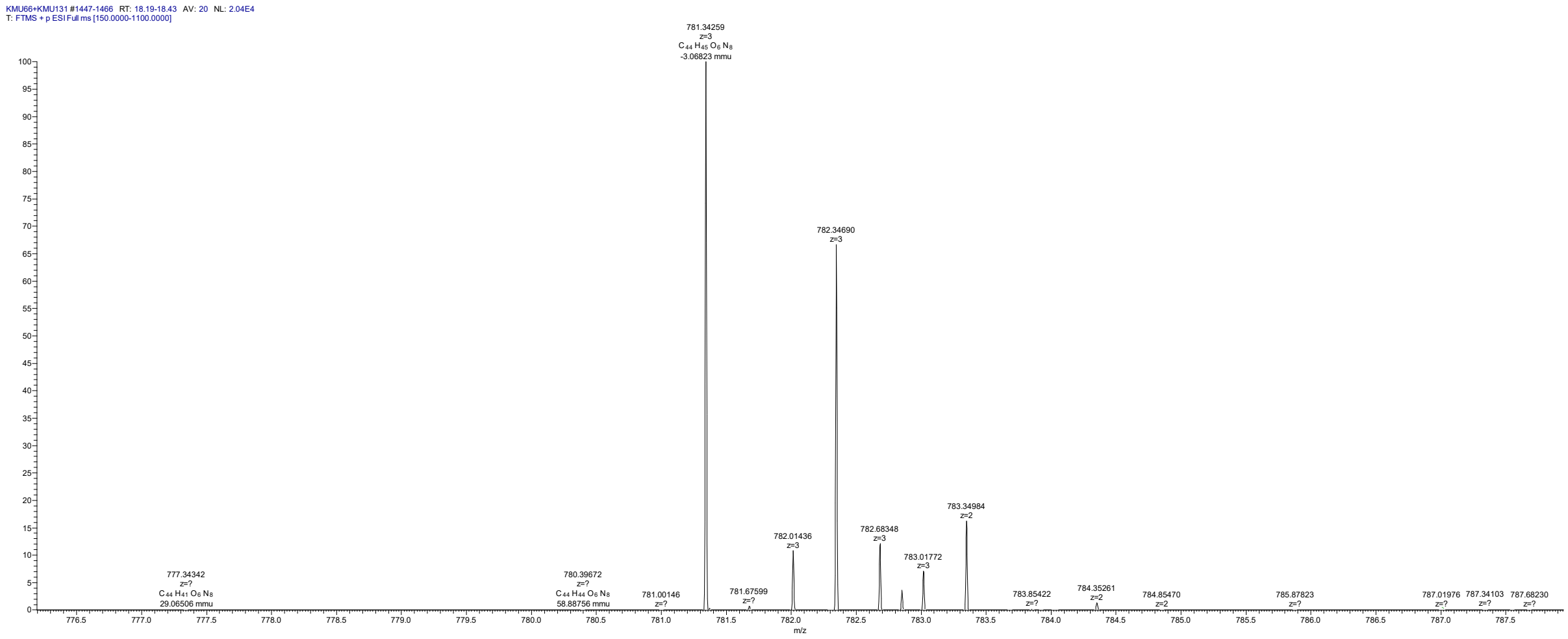

Figure S58. MS analysis (ESI) of product **32**.

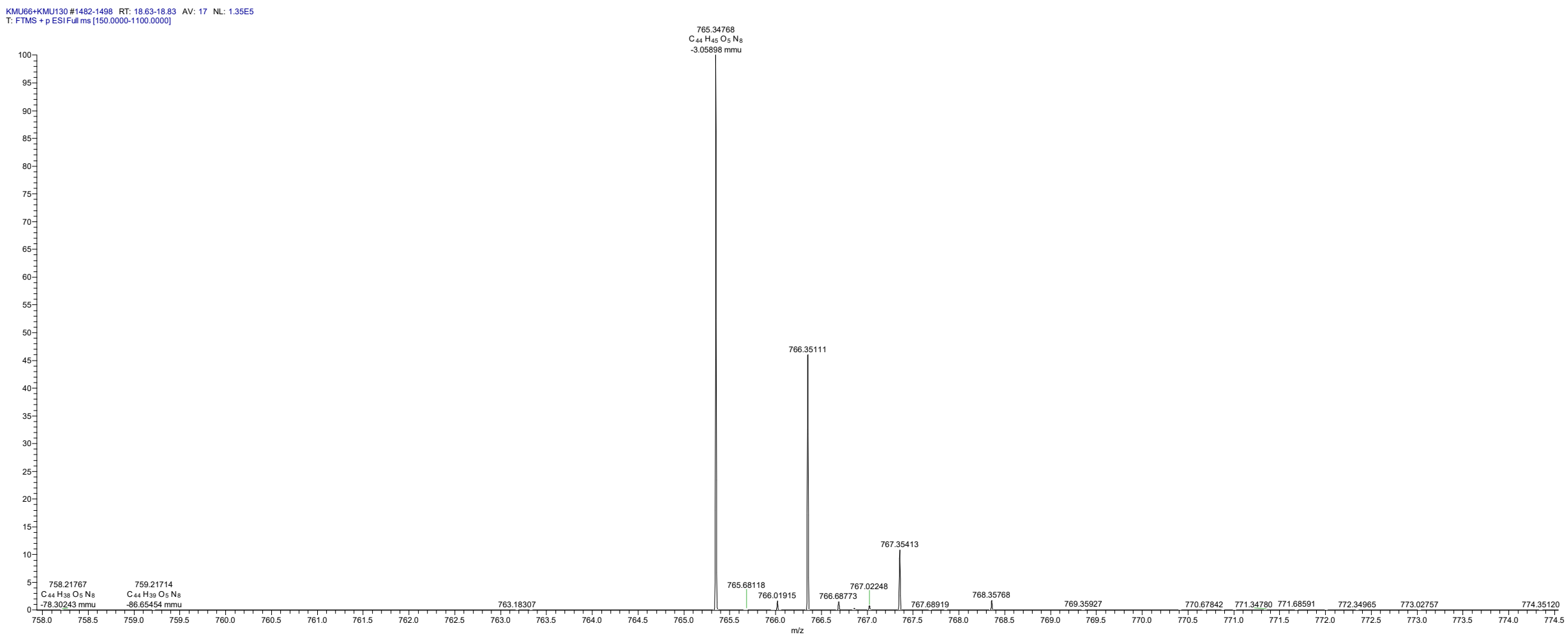

Figure S59. MS analysis (ESI) of product **33**.

SPSAC of 1-5 with DNA-BCN

Quartz cuvettes with a light path of 10 mm and a probe volume of 0.50 mL were used. The stock solution of **DNA-BCN** was prepared in water and the stock solutions of dyes **1**-**5** were diluted 1:10 with DMSO to obtain a concentration of 400 µM. This resulted in an end concentration of 2.0 µM dye **1**-**5** and 10 µM **DNA-BCN** in water/DMSO 99:1 (**table 17**). The fluorescence spectra were measured on a Fluoromax-4 spectrometer of the company HORIBA-SCIENTIFIC with a Peltier-element for temperature control. The measurements were corrected for Raman scattering of the respective solvent. Fluorescence spectra were recorded time-dependently to monitor the increase in fluorescence intensity (Figure 3) and were fitted using a monoexponential function as described above. **DNA-3, DNA-4** and **DNA-5** were identified by ESI-MS analysis (Table S5 and Figures S60-S61).

Table S4. Sample preparation for determining rate constants for SPSAC of the **1-5** with **DNA-BCN**.

	$c_{1\text{-}5}$ [μM]	V_{stock} **1-5** [μL]	$c_{\text{DNA-BCN}}$ [μM]	V_{stock} **DNA-BCN** [μL]	V_{water} [μL]	V_{DMSO} [μL]
Blank	0	0	0	0	495	5
Blank SPSAC	0	0	10	10	485	5
t_0	2	2.5	0	0	495	2.5
t	2	2.5	10	10	485	2.5

Table S5. MS analysis (ESI) of products **DNA-3**, **DNA-4** and **DNA-5**.

product	calculated mass [m/z]	measured mass [m/z]
**DNA-3**	1456.8 (z=4)	1456.05 (z=4)
**DNA-4**	1456.8 (z=4)	1456.05 (z=4)
**DNA-5**	1418.05 (z=4)	1417.28 (z=4)

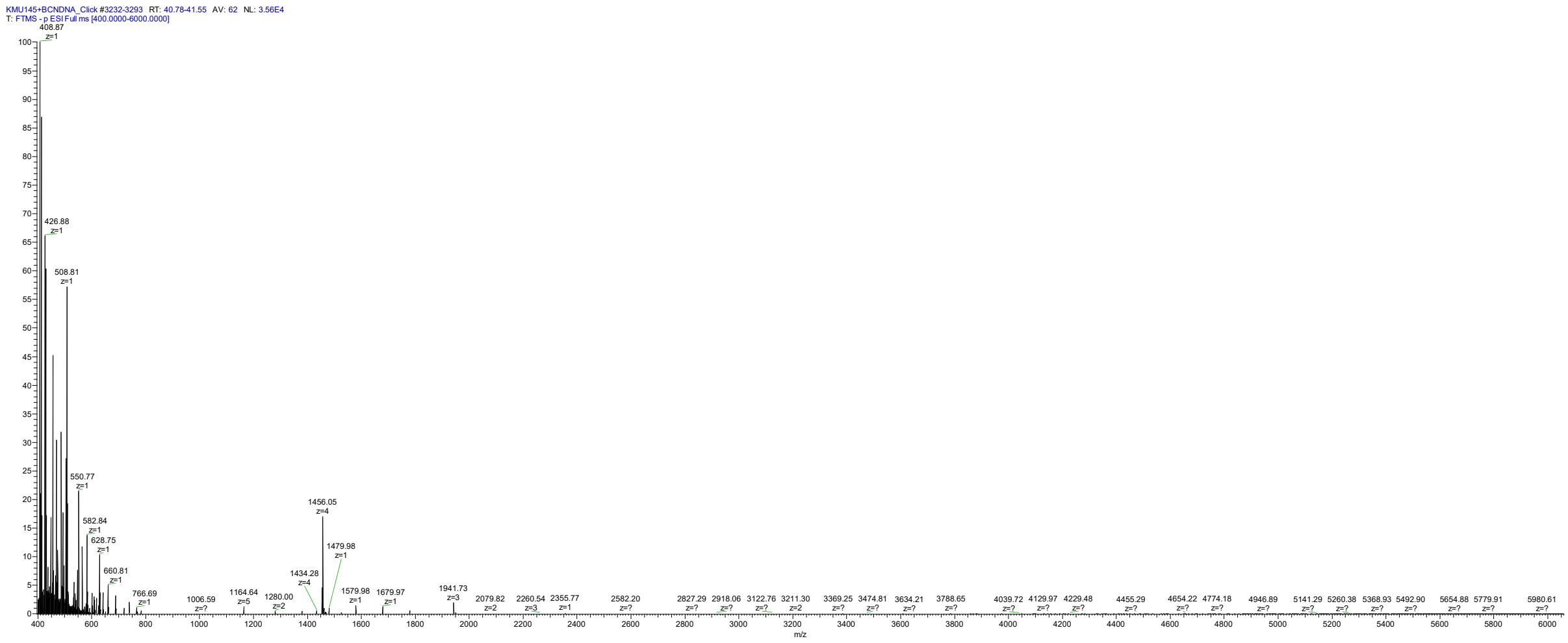

Figure S60. MS analysis (ESI) of product **DNA-3**.

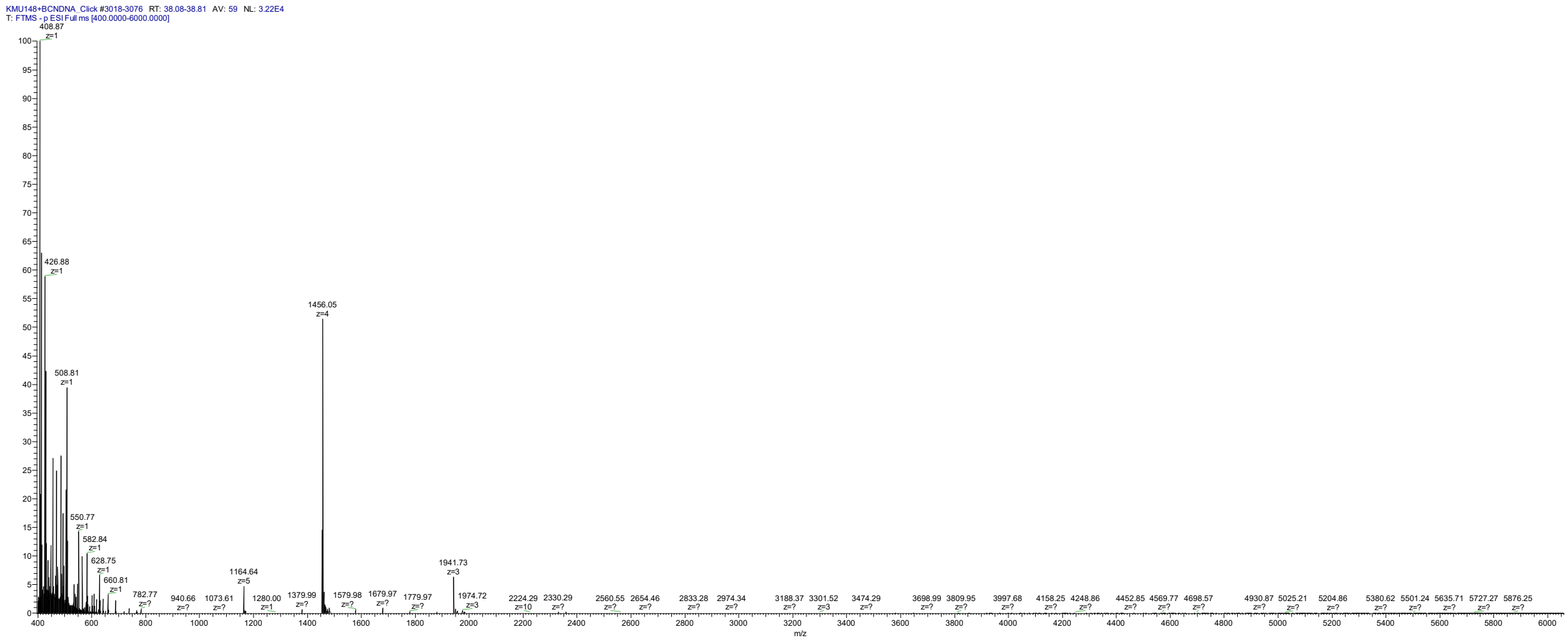

Figure S61. MS analysis (ESI) of product **DNA-4**.

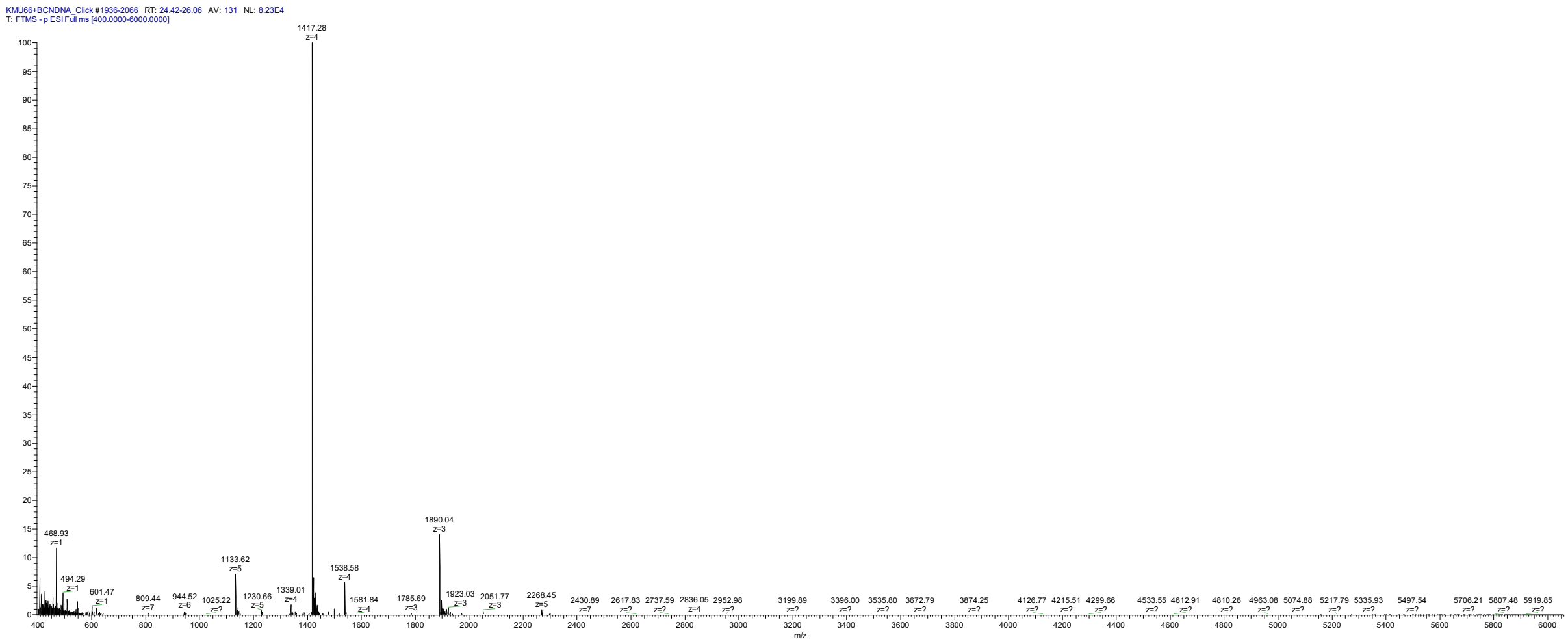

Figure S62. MS analysis (ESI) of product **DNA-5**.

5. Cell experiments

All cell experiments were carried out under sterile conditions. The materials and equipment used, as well as protective gloves, were disinfected with an 80 % ethanol solution before use. Consumables that were not sterile-packaged were first autoclaved before use.

For transfection experiments, human cervical carcinoma cells (HeLa) from the company ATCC, were cultured under sterile conditions in Dulbecco's modified Eagle medium ([+] 4.5 g/L D-glucose, L-glutamine, [+] pyruvate, DMEM, Gibco™), supplemented with 10 % fetal calf serum (HI FBS, Gibco™) and 1 U/mL penicillin-streptomycin (Gibco™). The cells were incubated in a BINDER incubator at 37 °C, 5 % CO_2 and humidity of 90 %. The cells were passaged at a confluence of approximately 80 %. First the medium was removed and the cells washed with 5 mL Dulbecco's Phosphate Buffered Saline (DPBS, Gibco™). To detach the cells from the bottom of the cell culture flask, 1 mL of a 0.25 % trypsin/EDTA solution were put on the cells and the cells were incubated for 3 min at 37 °C. Then 9 mL DMEM was put on the cells to stop the trypsination and the cell suspension was transferred to a new cell culture flask in the appropriate dilution with fresh medium. For subsequent cell experiments, the cell number was determined using a Neubauer counting chamber. In a next step, the cells were diluted to the desired cell number and transferred to µ-slides 8-well (IBIDI).

For the transfection experiments $2 \cdot 10^4$ cells per well were seeded in a volume of 200 µL in an IBIDI (µ-slide 8 well ibiTreat, IBIDI) plate. Simultaneously, the oligonucleotide was transfected using Lipofectamine (Lipofectamine™ 2000, Invitrogen). For this purpose, 0.15 µL Lipofectamine™ stock solution was mixed with 4.85 µL Opti-MEM™ (serum-reduced medium, Gibco™) per well with 150 nM of **DNA-BCN** or the in vitro clicked **DNA-1**, **DNA-2**, **DNA-3** (positive control) and incubated for 20 min at room temperature. The formed lipoplex was pipetted into the cell suspension in the IBIDI well and 2 µL Endoporter (GENE TOOL) per well was added, and the IBIDI was put on the laboratory shaker for 1 min. After the cells were incubated for 24 h the cell culture medium was removed and the cells were washed with DPBS. 4.00 % paraformaldehyde solution was put on the cells and for 1 min incubated on the laboratory shaker. The fixation was stopped by adding 50.0 mM glycine and 50.0 mM NH_4Cl in DPBS for 5 minutes. After washing the cells two times with DPBS 150 nM (**3**), 300 nM (**4**) and 600 nM (**5**) (stock solution in DMSO, diluted in DMEM) was added. 2 h later the cells were washed twice with DPBS (2 x 15 min). Draq5 was added to stain DNA and the cells were examined using a confocal fluorescence microscope (Leica DMI8, TCS SP8 inverted microscope) with a HC PL APO 63x/1.40 OIL CS2 objective. The fluorophore conjugates were excited with a 488 nm laser (10 % intensity) (**3**/**DNA-3** and **4**/**DNA-4**) and 405 nm (10 % intensity) (**5**/**DNA-5**) and the emission was measured in the range of 520 – 650 nm.

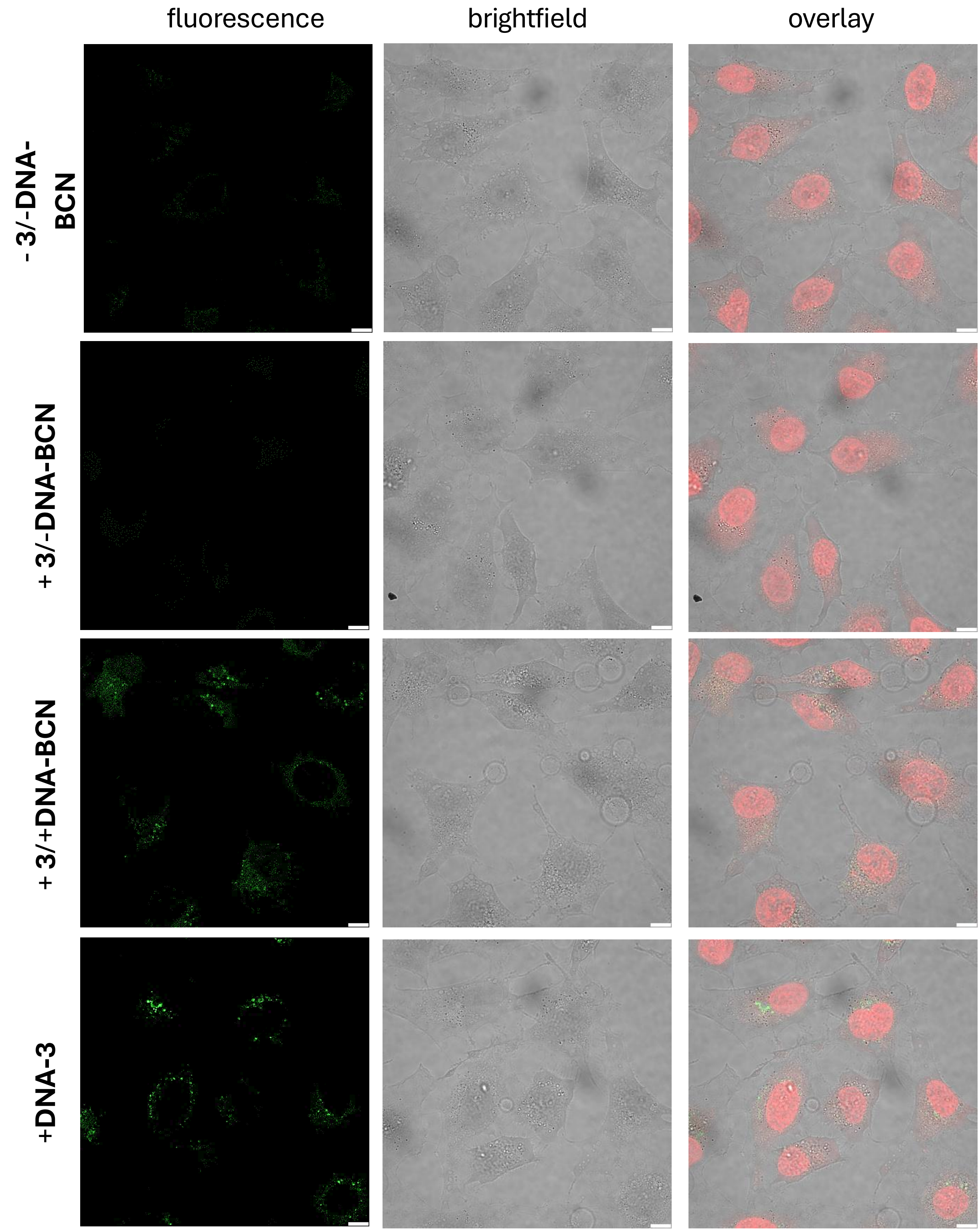

Figure S63. Fluorescence microscopic images of fixed HeLa cells treated for 2 h with **3**. Negative control: untreated HeLa cells and **3** without transfected DNA. Positive control: DNA-3. λ_{exc} = 488 nm (10 % intensity), $\lambda_{em.}$ = 500-600 nm, Scale bar: 10 µm. Draq5 was added to stain DNA

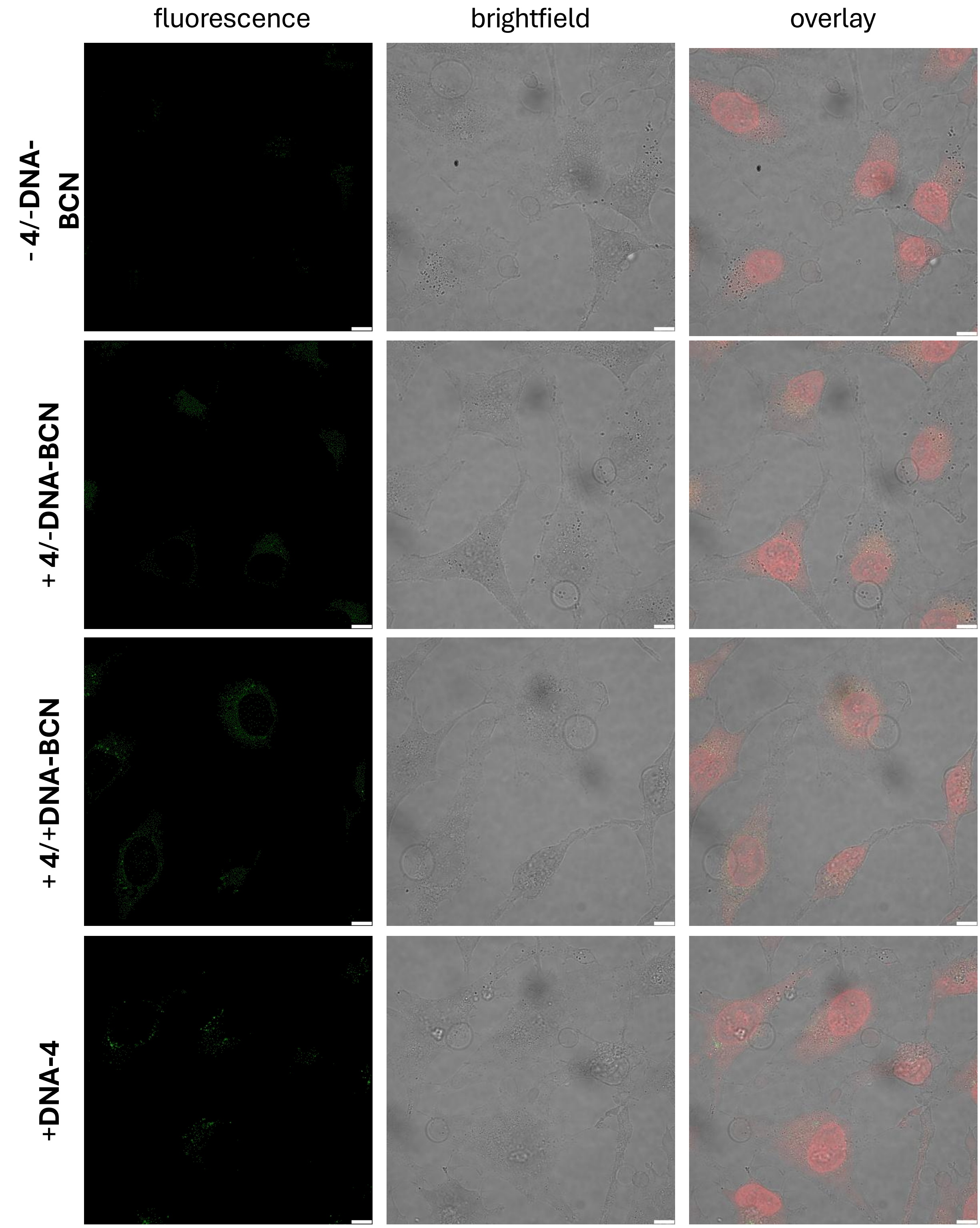

Figure S64. Fluorescence microscopic images of fixed HeLa cells treated for 2 h with **4**. Negative control: untreated HeLa cells and **4** without transfected DNA. Positive control: **DNA-4**. λ_{exc} = 488 nm (10 % intensity), $\lambda_{em.}$ = 500-600 nm, Scale bar: 10 µm. Draq5 was added to stain DNA.

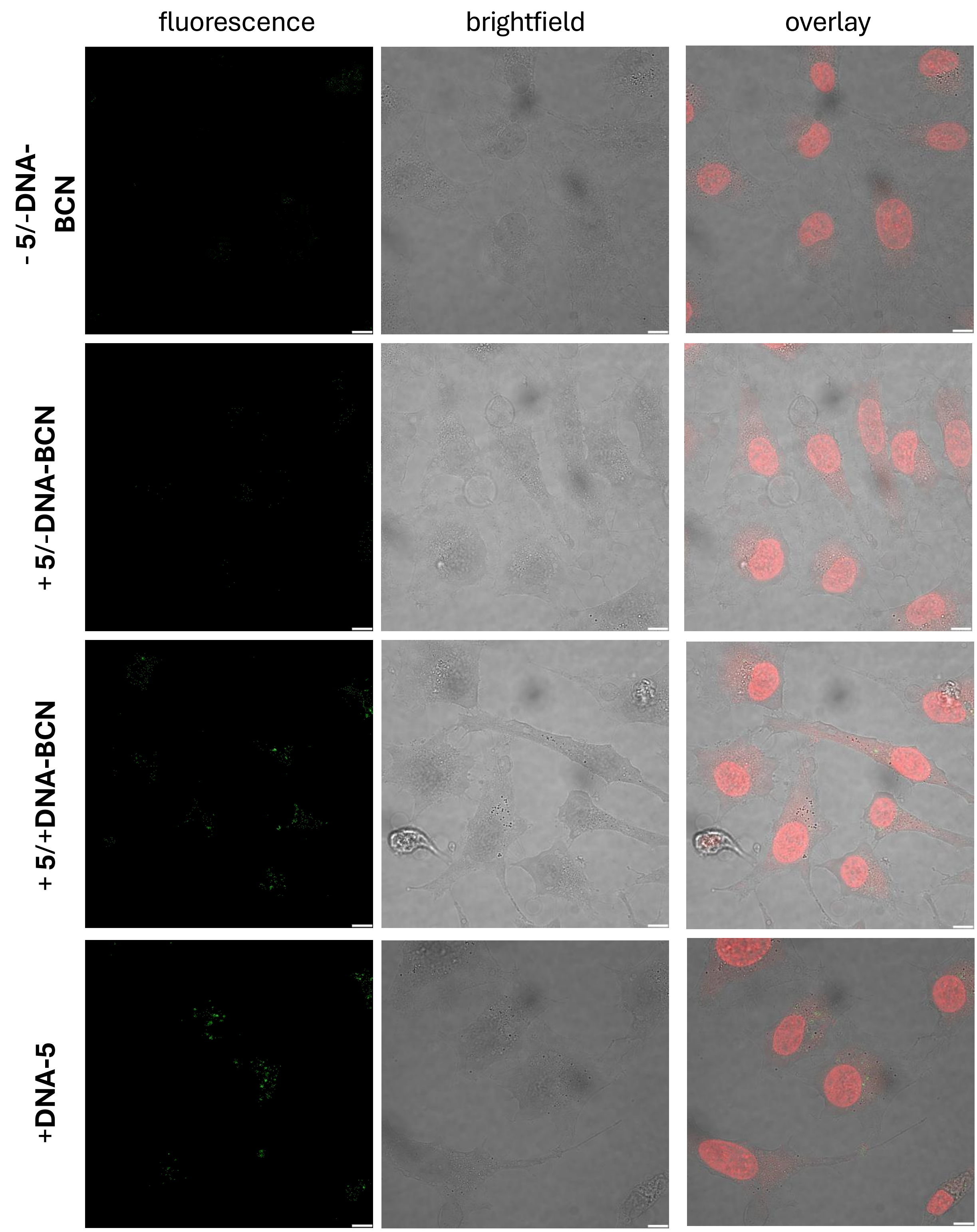

Figure S65. Fluorescence microscopic images of fixed HeLa cells treated for 2 h with **5**. Negative control: untreated HeLa cells and **5** without transfected DNA. Positive control: **DNA-5**. λ_{exc} = 405 nm (10 % intensity), $\lambda_{em.}$ = 500-600 nm, Scale bar: 10 µm. Draq5 was added to stain DNA.
